# Steric Control over the Threading of Pyrophosphonates with One or Two Cyanostar Macrocycles during Pseudorotaxane Formation

**DOI:** 10.1002/chem.202300899

**Published:** 2023-06-09

**Authors:** Julian Vogel, Yusheng Chen, Rachel E. Fadler, Amar H. Flood, Max von Delius

**Affiliations:** a Institute of Organic Chemistry, Ulm University, Albert-Einstein-Allee 11, 89081 Ulm (Germany); b Department of Chemistry, Indiana University, 800 E. Kirkwood Avenue, Bloomington, IN 47405 (USA)

**Keywords:** anion binding, host-guest chemistry, macrocycles, phosphorus chemistry, rotaxanes

## Abstract

The supramolecular recognition of anions is increasingly harnessed to achieve the self-assembly of supramolecular architectures, ranging from cages and polymers to (pseudo)rotaxanes. The cyanostar (**CS**) macrocycle has previously been shown to form 2:1 complexes with organophosphate anions that can be turned into [3]rotaxanes by stoppering. Here we achieved steric control over the assembly of pseudorotaxanes comprising the cyanostar macrocycle and a thread that is based, for the first time, on organo-pyrophosphonates. Subtle differences in steric bulk on the threads allowed formation of either [3]pseudorotaxanes or [2]pseudorotaxanes. We demonstrate that the threading kinetics are governed by the steric demand of the organo-pyrophosphonates and in one case, slows down to the timescale of minutes. Calculations show that the dianions are sterically offset inside the macrocycles. Our findings broaden the scope of cyanostar-anion assemblies and may have relevance for the design of molecular machines whose directionality is a result of relatively slow slipping.

## Graphical Abstract

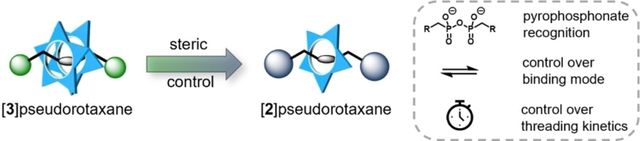

## Introduction

**Figure 1.**
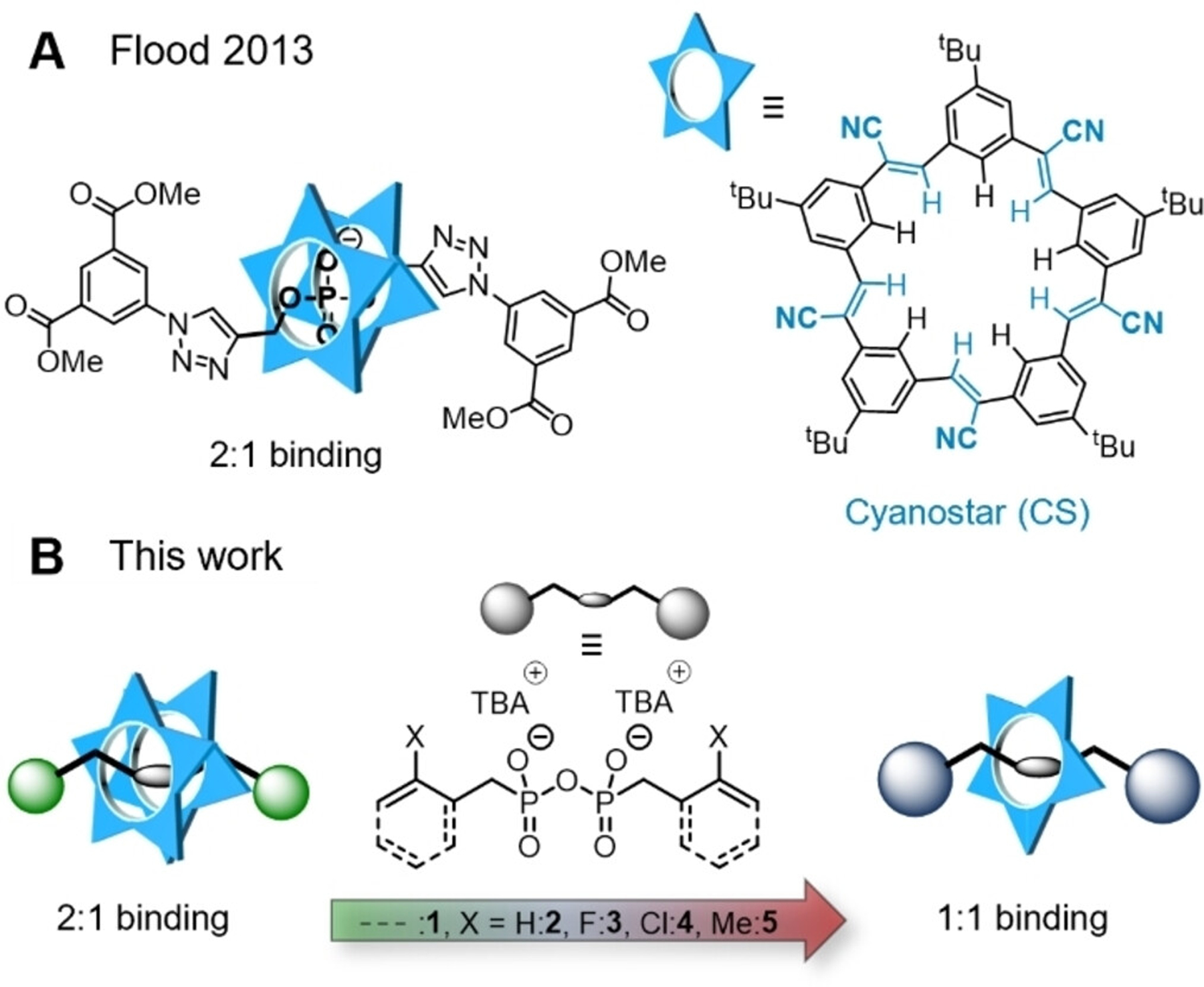
Overview on (A) previous work and (B) the scope of this study.

Since the first reports half a century ago,^[1–4]^ the field of supramolecular anion recognition^[5,6]^ and anion receptor chemistry^[7–13]^ has made significant advances. Notable applications^[14]^ of anion recognition include sensing,^[15–17]^ membrane transport,^[18,19]^ catalysis^[20–24]^ and increasingly the self-assembly of supramolecular architectures.^[25–34]^ Anion recognition has also been used to synthesize mechanically interlocked molecules.^[35–38]^ In most of these interlocked examples, anions play the role of a template, such that the anion is not part of either the thread or the macrocycle and can be removed after synthesis.^[39–42]^ Following one pioneering example based on a phenolate in the thread,^[43]^ dialkyl phosphates have been pursued as promising binding sites to furnish anion-containing rotaxanes. While this approach comes with its challenges,^[44]^ Flood and coworkers reported a strategy for the synthesis of [3]rotaxanes that are based on dialkyl phosphates and large anion-binding macrocycles (Figure 1A).^[45,46]^

The recognition of phosphorylated molecules,^[47,48]^ including phosphates^[49–52]^ and inorganic pyrophosphate^[53–57]^ has been investigated extensively. By contrast, the supramolecular chemistry of organic pyrophosphates is underexplored^[48,58,59]^ and, to the best of our knowledge, the recognition of organic pyrophosphonates is unexplored. As dianions with tunable organic substituents, these compounds would extend the scope of anion recognition towards designer guest molecules,^[60–62]^ which could play a role in carbodiimide-driven chemical reaction cycles.^[63–69]^

A promising candidate for the binding of such large anions is the cyanostar (**CS**) macrocycle (Figure 1A)^[45]^ that has a positively polarized central cavity in which it can bind anions through multiple CH hydrogen bonds. Cyanostar has been reported to bind organophosphates and organophosphonates in co-assemblies, where two (or more) macrocycles π-stack in a dimeric fashion with multiple equilibria.^[61,70–73]^ This relatively strong interaction has enabled supramolecular polymerization^[72]^ and reversible adhesion.^[62]^ The 1:1 binding of only one cyanostar to an anion has only been observed once for a bulky derivative (i. e., cyanosolo),^[74]^ but never for the parent macrocycle.

Herein, we report that control over the stoichiometry and kinetics of **CS**:anion binding can be achieved by modifying the steric bulk on organo-pyrophosphonate threads. A series of five pyrophosphonates was synthesized (Figure 1B) and their thermodynamics and kinetics of binding with cyanostar were studied. We found that binding can be switched between the 2:1 and the 1:1 stoichiometry, which represents the selective formation of [3]- and [2]pseudorotaxanes, respectively.

## Results and Discussion

To study their interaction with the cyanostar macrocycle, we synthesized five different pyrophosphonates (**PP**) as *bis-*tetrabutylammonium (TBA) salts by condensation of the corresponding phosphonate with diisopropylcarbodiimide (DIC). Full synthesis procedures and characterization by NMR spectroscopy (including ^31^P NMR) and high-resolution mass spectrometry (HRMS) is provided in the Supporting Information. The pyrophosphonates differ in their steric bulk, ranging from the least sterically demanding homoallyl-PP **1** to benzyl-PPs with different substituents in the *ortho*-position. Substitution with –H (**2**), –F (**3**) and –Cl (**4**) atoms or the methyl group (–Me, **5**) provides a subtle stepwise increase in steric demand. The dianionic pyrophosphonate in the center of the dumbbell-like structure can act as a binding site for the cyanostar macrocycle. The structures are depicted in Figure 1B. The cyanostar was synthesized as reported previously^[45]^ (Figure S45–46).

### Thermodynamics of binding

**Figure 2.**
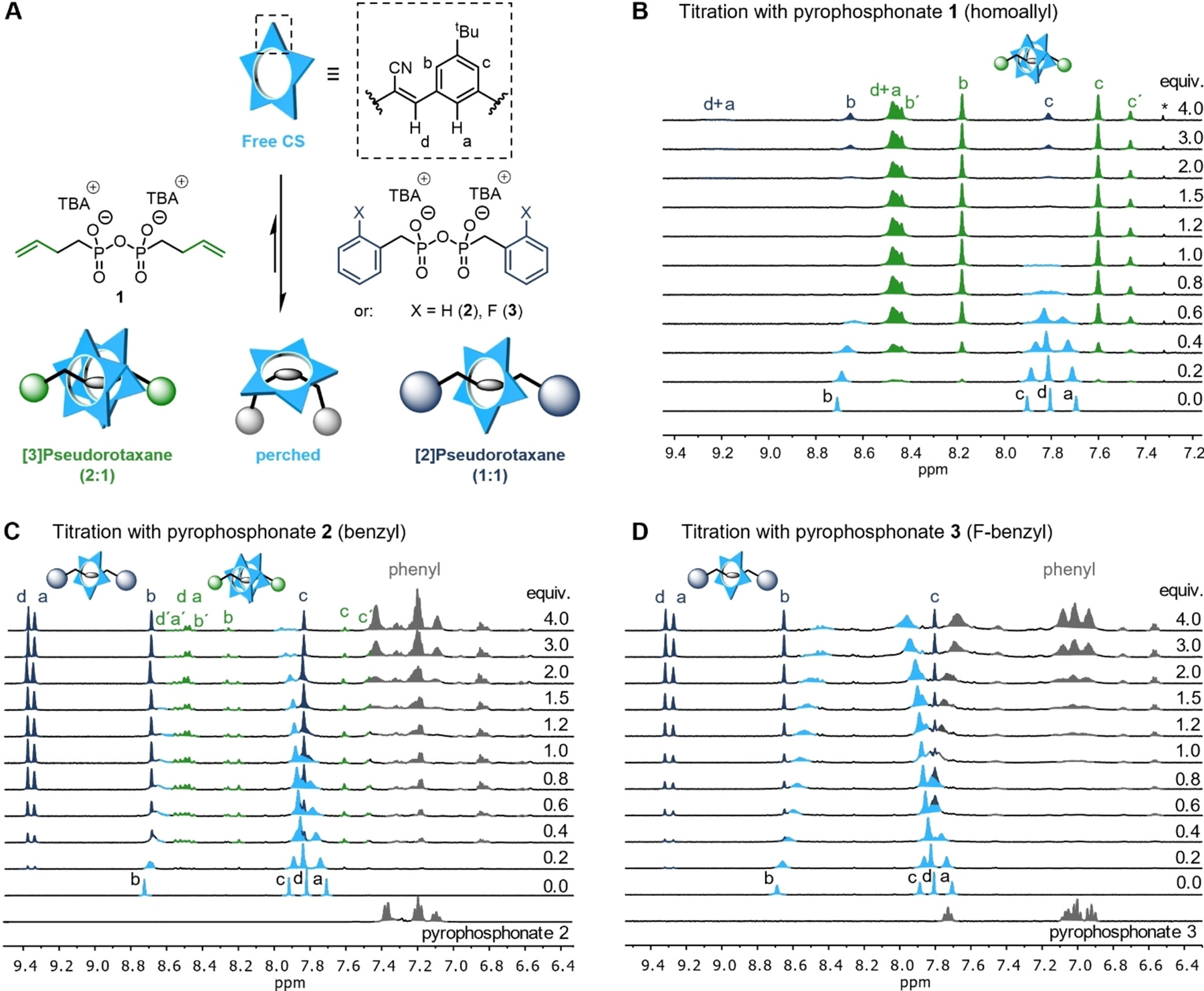
^1^H NMR titrations. (A) Schematic illustration of experiments leading to either [*n*]pseudorotaxanes (*n* = 2, 3) or the perched complex. (B) Titration of homoallyl-PP **1** into a solution of **CS** leads to the formation of [3]pseudorotaxane (2:1 complex). (C) Titration of benzyl-PP **2** to **CS** leads to a mixture of 1:1 and 2:1 complexes. (D) Titration of F-benzyl-PP **3** to **CS** leads exclusively to the formation of [2]pseudorotaxane (1:1 complex). The corresponding signals are color-coded as follows: free and perched **CS** generate a single fast-exchange signature colored cyan, the 2:1 complex is green, and the 1:1 complex is blue, while the phenyl-protons (free and bound) are grey. ^1^H NMR titrations (600 MHz) were performed with 0.5 mM **CS**, at 298 K in DCM-d_2_.

To investigate complexation, we performed ^1^H NMR titrations by adding the pyrophosphonate threads to the cyanostar (0.5 mM, DCM-d_2_, Figure 2A). As depicted by the cartoons in Figure 2A, there are three different binding modes possible: Either a threaded 2:1 complex (green, [3]pseudorotaxane), a threaded 1:1 complex (blue, [2]pseudorotaxane) or a perched 1:1 complex (cyan), in which the cyanostar is associated with the pyrophosphonate binding site but not threaded with it. In the case of the least sterically demanding homoallyl-PP thread **1** (Figure 2B), we observed the expected formation of the characteristic peaks for the cyanostar dimer (green) that are in slow exchange on the NMR timescale as reported previously.^[70,72]^ Because the macrocycle dimer can arrange either as a *chiral* (‘) or as a *meso* isomer, two sets of diastereomeric peaks can be observed (structures shown in Figure S69). Due to its larger pseudo-stopper, addition of the benzyl-PP **2** to cyanostar (Figure 2C) yielded the [3]pseudorotaxane (2:1 binding) only in minor amounts. Instead, we observed a new set of ^1^H NMR signals (blue) that belongs to the 1:1 binding mode corresponding to a [2]pseudorotaxane. In both complexes we observed a strong downfield shift for protons H_*a*_ and H_*d*_ that point inside the binding cavity and is consistent with CH hydrogen bonding to the pyrophosphonate anions. Interestingly, the [3]pseudorotaxane displays shifts in H_*a*_ and H_*d*_ that more closely resemble the shifts seen upon complexation of dianions^[70]^ rather than monoanions.^[45]^ The minor shift seen for protons H_*b*_ and H_*c*_ are consistent with their locations on the outside of the macrocycles. Likewise, we exclusively observed the formation of [2]pseudorotaxane when performing the analogous titration with F-benzyl-PP **3** (Figure 2D), where the steric demand is further increased. Binding also leads to a new set of signals for the pyrophosphonates phenyl protons (grey, shifted upfield under the influence of cyanostar’s π-system). When increasing the equivalents of the pyrophosphonate up to 50, we could observe that the equilibrium is shifted towards the [2]pseudorotaxane even in the case of homoallyl-PP **1** (Figure S64 and Figure S70), because the cyanostar availability is decreased.

For all investigated threads, the signals of the unthreaded cyanostar (cyan) broaden upon addition of excess pyrophosphonate as a result of forming a perched complex that is in fast exchange with the free cyanostar on the NMR timescale.The sharp signals for both the 2:1 and the 1:1 pseudorotaxanes indicate slow exchange on the NMR timescale. For the two most sterically demanding pyrophosphonates **4** and **5** (–Cl, –Me), no evidence of threading was observed. Even at increased temperatures up to 403 K (VT NMR, Figure S67), ^1^H NMR spectroscopy did not indicate any rotaxane formation in C_2_D_2_Cl_4_ (upon cooling to ambient temperature), from which we conclude that even the Cl-substituted stopper is already too large to allow slipping of cyanostar. Further fine-tuning of the stoppers (e. g., five-membered rings) could potentially facilitate a convenient synthesis of cyanostar rotaxanes by a slipping approach (heating followed by cooling).^[75,76]^

To complete the characterization of the complexes, we measured high resolution mass spectra (HRMS) of the pseudorotaxanes and found corresponding signals to be in good agreement with the calculated isotopic patterns (Figures S74–75). When applying a collision voltage to the pseudorotaxane in the gas-phase, the signal decreased in favor of the free pyrophosphonates indicating dethreading (Figure S76). The binding of the cyanostar to the pyrophosphonates in solution was further corroborated by ^1^H–^1^H NOE NMR spectroscopy, where a cross-peak between the inner protons of the cyanostar (H_*a*_ and H_*d*_) and the benzyl protons of the pyrophosphonate was observed (Figure S73).

The binding was investigated further by ^31^P NMR spectroscopy. In a titration experiment, we added cyanostar to benzyl-PP **2** and F-benzyl-PP **3** (2.5 mM, DCM-d_2_ or CDCl_3_) and monitored the decreasing peak of the free pyrophosphonate and the increasing peak of [2]pseudorotaxanes. As the species are in slow exchange, we were able to integrate the two peaks and determine binding constants by applying the mass action law (Figures S77–S80). All binding constants are moderate and on the order of 10^3^ M^−1^ (see Table 1). Unfavorable steric interactions could account for the weaker binding compared to previously studied anions.^[45,74]^

### Kinetics of binding

**Figure 3.**
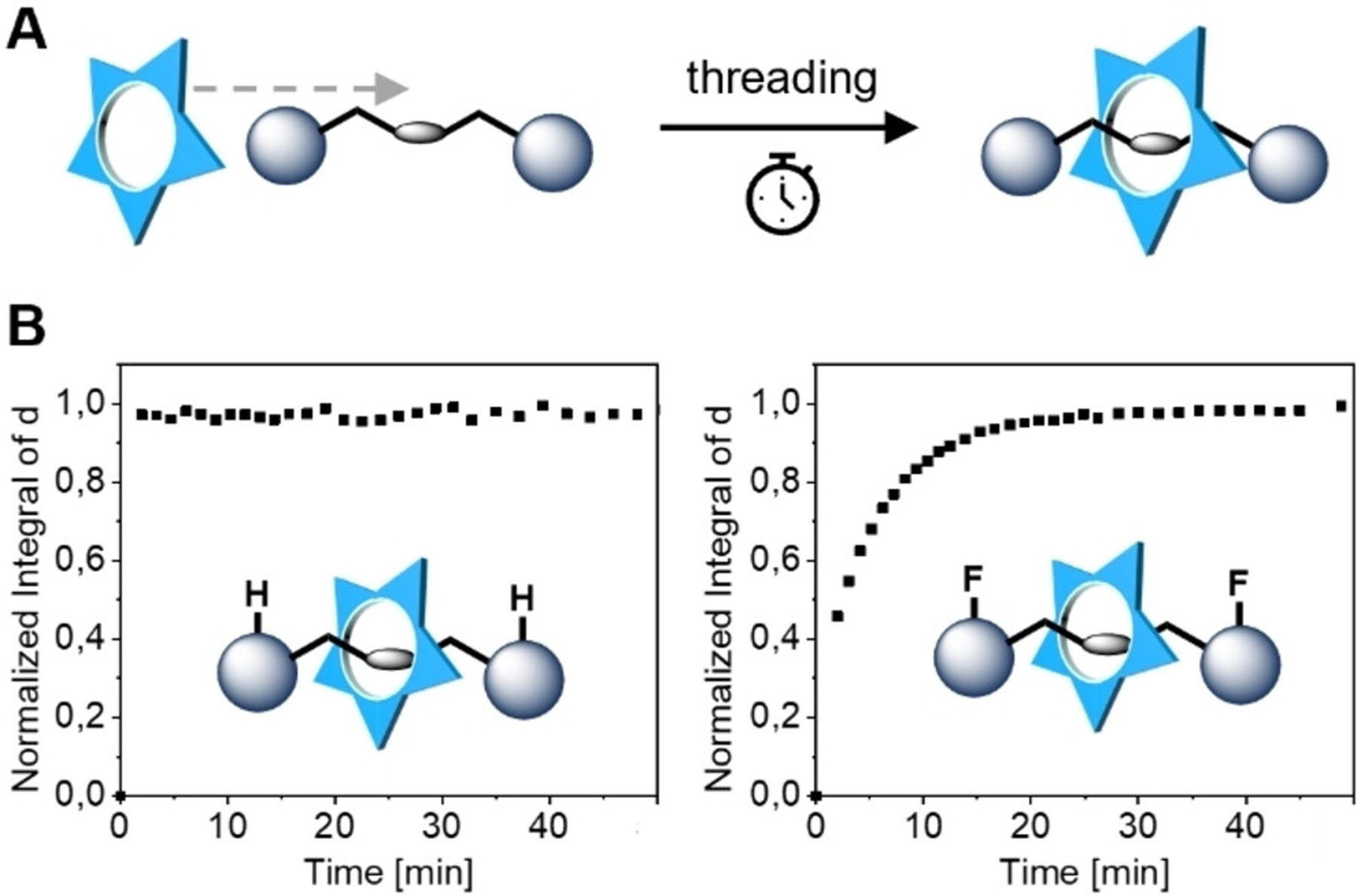
Binding kinetics. (A) Illustration of the experiment: The threading kinetics of cyanostar on the pyrophosphonates is monitored. (B) Formation of the [2]pseudorotaxane (1:1 binding) over time (as indicated by the integral of the characteristic H_*d*_ proton) with pyrophosphonates **2** and **3**, respectively. All kinetic experiments were performed at 298 K, 1 mM in DCM-d_2_.

We also explored the kinetics of threading cyanostar macrocycles onto the pyrophosphonates. As illustrated in Figure 3A, we added the pyrophosphonates (2 mM) to a solution of cyanostar in DCM-d_2_ (1 mM) and monitored the formation of the corresponding pseudorotaxanes by ^1^H NMR spectroscopy. For homoallyl-PP **1** (Figure S58) and benzyl-PP **2** (Figure 3B and S59) the threading is too fast to be observed in these experiments. Taking the delay between addition of the pyrophosphonate and the first measurement into account, the time until 50 % of the final equilibrium is reached (*t*_50_) is therefore below 20 s. When we studied the de-threading kinetics of the 1:1 complex between cyanostar and benzyl-PP **2**, we found that it made a difference whether we initiated de-threading by addition of PF_6_^−^ (relatively slow de-threading with t_50_≈120 s; Figure S61) or water (fast de-threading with t_50_<20 s, Figure S62). The steric demand of F-benzyl-PP **3** (Figure 3B and Figure S60) slowed down the threading significantly and we determined *t*_50_≈3.5 min. Equilibrium is reached after approximately 15 min (Figures S48–49), so this compound can be considered a borderline case between pseudorotaxane and rotaxane.^[77]^ Such relatively slow threading kinetics could be interesting for the design of directional molecular machines,^[78–82]^ in which a ratcheting step relies on a molecular “valve”.^[83–86]^

Variable temperature (VT) NMR spectroscopy was performed (Figures S50–S53) to gain insights into the kinetics associated with formation of the perched interaction from the free cyanostar. As mentioned above, formation of this perched structure is fast on the NMR time scale at 298 K. Decreasing the temperature to 233 K slows down this process significantly, as indicated by peak splitting of the cyanostar signals into those that can be assigned to the free and perched structures (Figure S65). At 333 K, on the other hand, sharp peaks are observed for free cyanostar indicating fast kinetics (in CDCl_3_).

The results of both the thermodynamic and the kinetic studies are summarized in Table 1. We found that increasing the steric bulk of the pyrophosphonates - as indicated here by the A values^[89–91]^ - decreases the threading kinetics over (at least) one order of magnitude. The binding thermodynamics (2:1 vs. 1:1 vs. no binding) is strongly dependent on the size of the pseudo-stoppers, with the *K*_A_ value of the more sterically demanding F-benzyl-PP **3** being slightly lower than benzyl-PP **2** (in CDCl_3_).

### Molecular modeling

**Figure 4.**
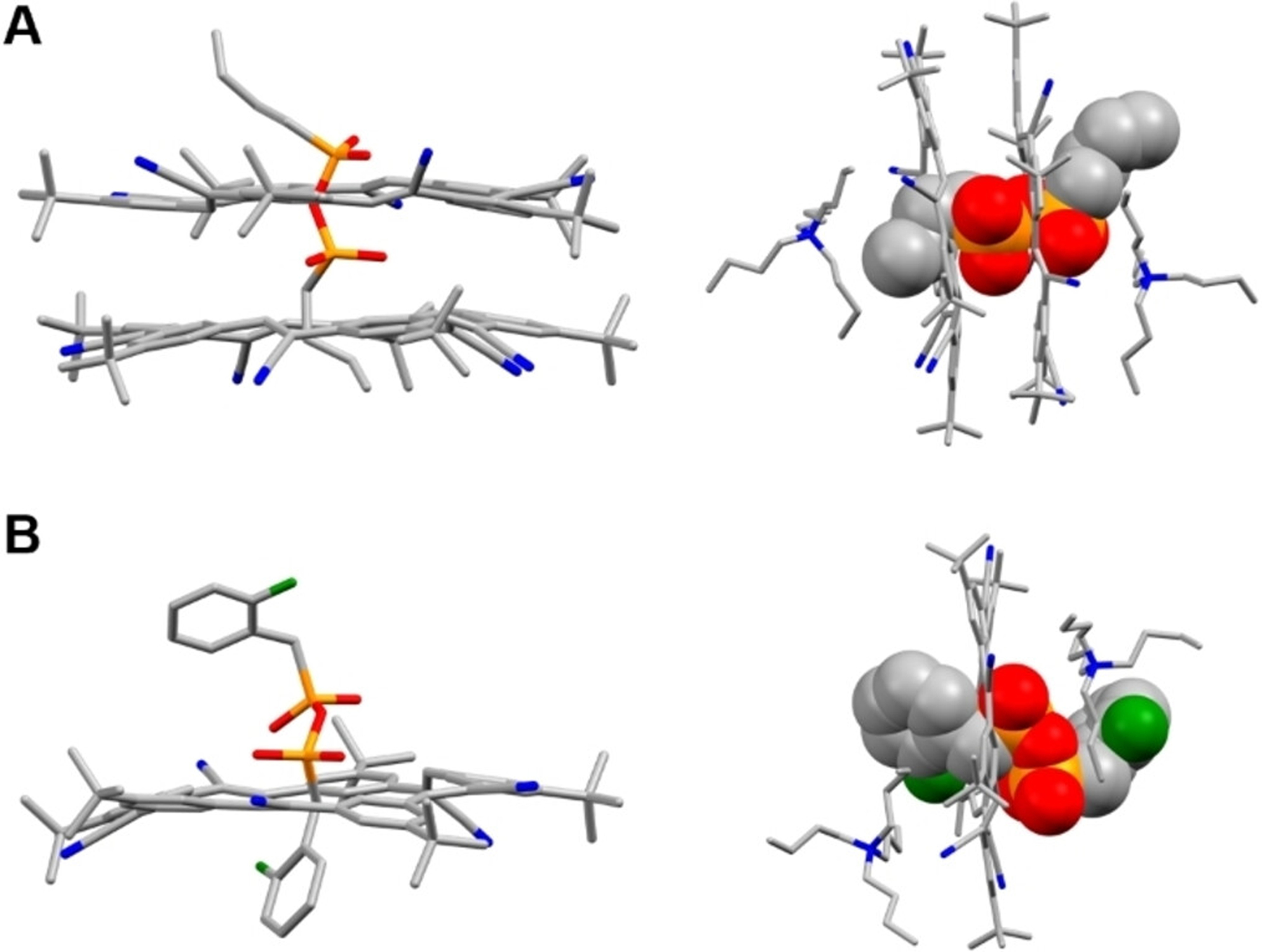
Equilibrium geometries of (A) the [3]pseudorotaxane comprised of two CS macrocycles and homoallyl-PP **1** and (B) the [2]pseudorotaxane comprised of one CS and F-benzyl-PP **3** shown as stick (left) and space-filling models (right). Equilibrium geometries were optimized using density functional theory (RB3LYP/6–31G(D)). Hydrogen atoms and TBA counter ions are omitted from the images on the left for clarity. Color code: C (grey), N (blue), O (red), F (green), P (orange).

In order to investigate how the steric demand of the pyrophosphonate dianions contribute to differences in the 1:1 and 2:1 binding modes, we performed density functional theory (DFT) calculations on the [3]pseudorotaxane with homoallyl-PP **1** and the [2]pseudorotaxane with F-benzyl-PP **3** (RB3LYP/6–31G(D), gas phase). Optimized geometries (Figure 4) were generated with the TBA^+^ cation but it was omitted in parts of the figure for the purpose of clarity (see Figures S81–85). Consistent with reported (pseudo)rotaxanes formed between phosphate threads and cyanostar,^[45,46,61]^ both structures show C–H···O hydrogen bonds between the polar cyanostar CH donors and the oxygen atoms on the pyrophosphonate threads. The [2]pseudorotaxane with F-benzyl-PP **3** shows an average hydrogen bond distance of 2.4 Å while the [3]pseudorotaxane with homoallyl PP **1** has an average hydrogen bond distance of 2.5 Å. In the case of the [3]pseudorotaxane with homoallyl PP **1**, we also observe a very short interaction (*d*_H···O_ = 2.1 Å) between α-proton of one TBA^+^ cation and an oxygen from the pyrophosphonate (Figure S83). This contact is consistent with reported crystal data of cyanostar and TBA-bisulfate^[92]^ or TBA-organophosphate^[70]^ complexes.

Interestingly, we observe that the π-stacked cyanostar dimer is not centered on the homoallyl PP **1** thread but shifted non-symmetrically to one side. To investigate the origin of this off-center position, we modelled binding of the simplest dianionic pyrophosphonate, H_2_P_2_O_5_^2−^, with the cyanostar dimer (Figure S84). Here we also observe the non-symmetric binding mode even without substituents, indicating the steric profile of pyrophosphonate itself does not allow centralization inside the cyanostar dimer. This steric outcome naturally leads to a more limited stabilization of the dianionic pyrophosphonate by the cyanostar(s) with the available external oxygen atom shown to form the short contact with the TBA^+^ cation. When the steric profile of the anionic thread becomes even more demanding, as with F-benzyl-PP **3** (Figure 4B and Figure S85), only one cyanostar can bind to the pyrophosphonate. This preference for one macrocycle emerges from the combination of the pyrophosphonate’s steric profile and the steric proximity of the fluorophenyl.

## Conclusions

**Figure 5.**
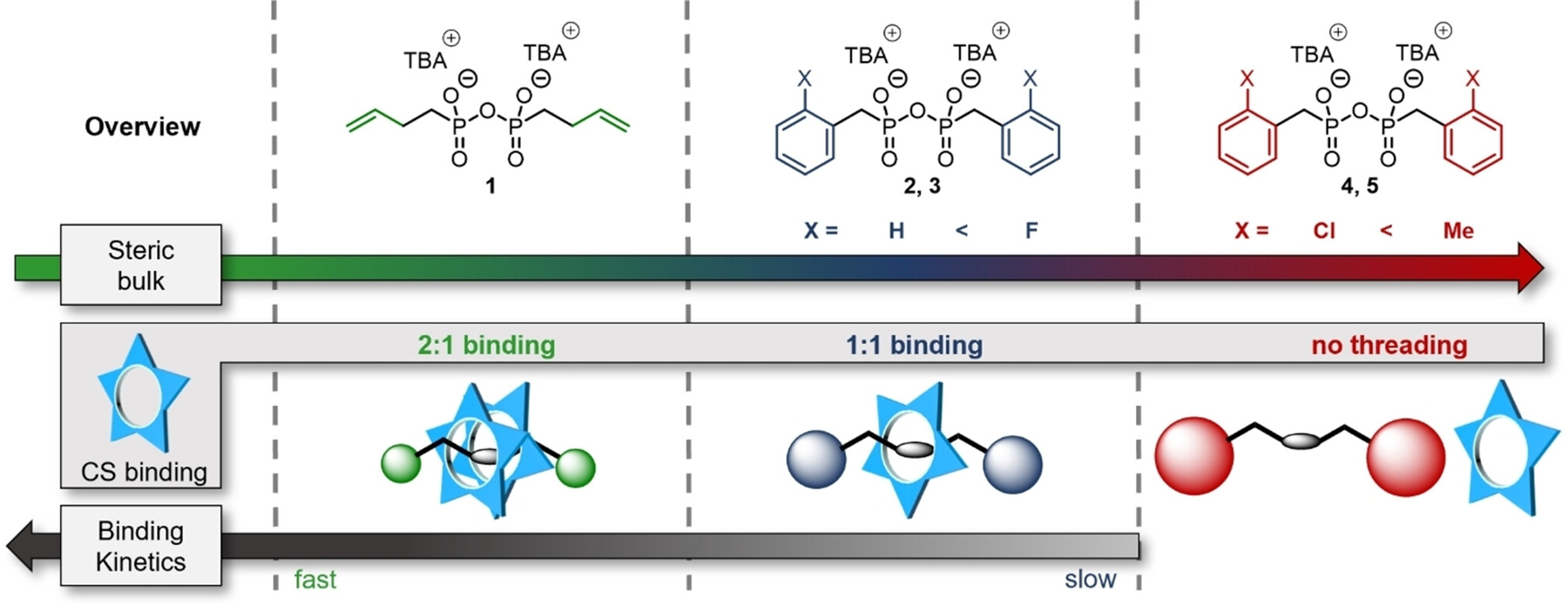
Overview of key findings of this study. Five different pyrophosphonates divided into three categories by their steric bulk.

We report the first example of organo-pyrophosphonate recognition and characterize their threading inside cyanostar macrocycles, which is modulated by the size of the organic substituent to afford pseudorotaxanes with one or two macrocycles (Figure 5). We synthesized a series of five short threads, explored their non-covalent C–H···O hydrogen bonding interactions with the anion-binding cyanostar macrocycle and characterized both thermodynamics and kinetics of threading. Using NMR titrations, we found that the previously reported propensity for cyanostar to form 2:1 complexes can be switched off completely by fine-tuning the steric profile of the pyrophosphonate. While DFT shows the pyrophosphonate anion is unable to perfectly fit inside two macrocycles, experiments show the homoallyl-PP alone can form a [3]pseudorotaxane. Increasing the steric demand yielded the successful assembly of a [2]pseudorotaxane with the F-benzyl-PP pyrophosphonate in chlorinated solvents. This first example of a 1:1 **CS**:anion complex was further corroborated by HRMS and NOESY NMR. We therefore demonstrate an approach to make use of substituents in designer anions with different steric demand to tune supramolecular recognition. Furthermore, we gained steric control not only over the thermodynamic binding mode, but also over the threading kinetics, which vary over at least one order of magnitude. Threading kinetics on the minutes-to-hours timescale represent the boundary between pseudorotaxanes and rotaxanes^[77]^ and can be harnessed to create directional molecular machines, which will be at the focus of future work.

## Experimental Section

### Synthesis of pyrophosphonates (PP):

Tetrabutylammonium phosphonates were dissolved in DCM and 10 equivalents of *N,N’*-diisopropylcarbodiimide (DIC) were added. The mixture was stirred at room temperature for 48 h. DCM was evaporated and the residue was purified by reversed-phase column chromatography. Lyophilization yielded the desired pyrophosphonates in 29–46 %.

### ^1^H NMR titrations:

All compounds were dried for 24 h under oil pump vacuum before use. A 0.5 mM solution of cyanostar in DCM-d_2_ (dried over 3 Å molecular sieves) was prepared. A freshly prepared stock solution of the corresponding pyrophosphonate (25 mM, DCM-d_2_) was added in a stepwise titration (1 μL = 0.1 equiv). The solutions were equilibrated according to their binding kinetics (see below) before measurement. All measurements were performed on a Bruker Avance 600 NEO spectrometer (600 MHz).

### High Resolution Mass Spectrometry Electrospray Ionization (HRMS-ESI):

A 200 *μ*M solution of a 1:1 mixture of cyanostar and the pyrophosphonate in HPLC-grade DCM was prepared and HRMS was measured with ESI in the negative ion mode on an Agilent 1260 Infinity II system with a 6546 LC/QTOF mass spectrometer. In the 2D-MS experiment a collision voltage of 10–30 V was applied to the [2]pseudorotaxane in the gas phase.

### ^31^P NMR titrations and determination of binding constants (*K*_A_):

500 μL of a 2.5 mM solution of the corresponding pyrophosphonate was prepared in CDCl_3_ or DCM-d_2_. A 50 mM solution of cyanostar in CDCl_3_ or DCM-d_2_ was added in a stepwise titration (5 μL = 0.2 equiv. cyanostar). ^31^P NMR was measured and the ratio of free pyro-phosphonate and [2]pseudorotaxane was obtained by relative integration. Concentrations (including cyanostar) were calculated considering the total volume. Binding constants (*K*_A_) were determined (as a mean value of seven measurements with the standard deviation as error) under the reasonable assumption that exchange is slow on the NMR time scale [Eq. (1)]:

(1) $$K_{A}=\frac{\left[HG\right]}{\left[G\right]\times \left[H\right]}=\frac{\left[pseudorotaxane\right]}{\left[pyrophosphonate\right]\times \left[CS\right]}$$

### Binding kinetics by ^1^H NMR monitoring:

25 μL of a cyanostar stock solution (20 mM in DCM-d_2_) was diluted with 435 μL DCM-d_2_. 40 μL of a stock solution (25 mM in DCM-d_2_) of the corresponding pyrophosphonate (**PP**) were added (1 mM **CS** + 2 mM **PP**) and a series of NMR spectra were recorded. A lag time of 120 s between addition of pyrophosphonate and the first measurement was taken into account. A characteristic peak for the formation of the [*n*]pseudorotaxane was monitored over time.

### Variable temperature (VT) NMR studies:

25 μL of a 20 mM cyanostar stock solution (in CDCl_3_) and 40 μL of a 25 mM stock solution of the corresponding pyrophosphonate (in CDCl_3_) were diluted to 500 μL with CDCl_3_ (1 mM **CS** + 2 mM **PP**). VT ^1^H NMR was measured starting from the lower temperature. The temperature was equilibrated for 5 min before each measurement.

### DFT calculations:

A restricted hybrid HF-DFT SCF calculation was performed using Pulay DIIS+geometric direct minimization SPAR-TAN’20 on the RB3LYP functional with a 6–31G(D) basis set for the geometry optimization.

## Supplementary Material

Supporting Information

## Figures and Tables

**Table 1.** Summary of binding thermodynamics and kinetics of the [2]pseudorotaxanes.^[87,88]^

Pyrophosphonate	A value^[a]^	*K*_A_ [M^−1^]^[b]^	*t*_50_^[c]^
1 (homoallyl)	n.a.	n.d.	< 20 s
2 (benzyl)	H: 0.0	2600±300 (CDCl_3_)	< 20 s
3 (F-benzyl)	F: 0.15	1300±300 (CDCl_3_) 4400±500 (CD_2_Cl_2_)	~ 3.5 min
4 (Cl-benzyl)	Cl: 0.43	No threading^[d]^	
5 (Me-benzyl)	Me: 1.70	No threading^[d]^	

[a] Indicates the size of the *ortho*-substituent on the phenyl group.^[89,90]^

[b] At 298 K, 2.5 mM. Only CDCl_3_ was used for benzyl-PP **2** where no 2:1 binding was observed due to the lower polarity of this solvent (compared to CD_2_Cl_2_).

[c] At 298 K, 1 mM in CD_2_Cl_2_.

[d] Up to 333 K in CDCl_3_ or up to 403 K in C_2_D_2_Cl_4_ (S52).

n.a.: not applicable.

n.d.: not determined (complex mixture).

## Data Availability

The data that support the findings of this study are available from the corresponding author upon reasonable request.

## References

[1] Simmons HE, Park CH, J. Am. Chem. Soc 1968, 90, 2428–2429.

[2] Simmons HE, Park CH, J. Am. Chem. Soc 1968, 90, 2429–2431.

[3] Simmons HE, Park CH, J. Am. Chem. Soc 1968, 90, 2431–2432.

[4] Lehn J-M, Sonveaux E, Willard AK, J. Am. Chem. Soc 1978, 100, 4914–4916.

[5] Molina P, Zapata F, Caballero A, Chem. Rev 2017, 117, 9907–9972. 28665114 10.1021/acs.chemrev.6b00814

[6] Boer SA, Foyle EM, Thomas CM, White NG, Chem. Soc. Rev 2019, 48, 2596–2614. 30860210 10.1039/c8cs00828k

[7] Schmidtchen FP, Berger M, Chem. Rev 1997, 97, 1609–1646. 11851460 10.1021/cr9603845

[8] Kang SO, Llinares JM, Day VW, Bowman-James K, Chem. Soc. Rev 2010, 39, 3980–4003. 20820597 10.1039/c0cs00083c

[9] Chen L, Berry SN, Wu X, Howe ENW, Gale PA, Chem 2020, 6, 61–141.

[10] Macreadie LK, Gilchrist AM, McNaughton DA, Ryder WG, Fares M, Gale PA, Chem 2022, 8, 46–118.

[11] Evans NH, Beer PD, Angew. Chem. Int. Ed 2014, 53, 11716–11754. 10.1002/anie.201309937 25204549

[12] Gale PA, Busschaert N, Haynes CJE, Karagiannidis LE, Kirby IL, Chem. Soc. Rev 2014, 43, 205–241. 24108306 10.1039/c3cs60316d

[13] Gale PA, Howe ENW, Wu X, Chem 2016, 1, 351–422.

[14] Busschaert N, Caltagirone C, Van Rossom W, Gale PA, Chem. Rev 2015, 115, 8038–8155. 25996028 10.1021/acs.chemrev.5b00099

[15] Kaur N, Kaur G, Fegade UA, Singh A, Sahoo SK, Kuwar AS, Singh N, TrAC Trends Anal. Chem 2017, 95, 86–109.

[16] Albelda MT, Frías JC, García-España E, Schneider HJ, Chem. Soc. Rev 2012, 41, 3859–3877. 22441360 10.1039/c2cs35008d

[17] Benasco AR, Tropp J, Kaphle V, Chen Y, Zhao W, Eedugurala N, Ng TN, Flood AH, Azoulay JD, Adv. Electron. Mater 2022, 8, 2101353.

[18] Liu Z, Wu LF, Xu J, Bonfio C, Russell DA, Sutherland JD, Nat. Chem 2020, 12, 1023–1028. 33093680 10.1038/s41557-020-00564-3 PMC7610406

[19] Davis JT, Okunola O, Quesada R, Chem. Soc. Rev 2010, 39, 3843–3862. 20820462 10.1039/b926164h

[20] Zhang Z, Schreiner PR, Chem. Soc. Rev 2009, 38, 1187–1198. 19421588 10.1039/b801793j

[21] Phipps RJ, Hamilton GL, Toste FD, Nat. Chem 2012, 4, 603–614. 22824891 10.1038/nchem.1405

[22] Zhao Y, Cotelle Y, Liu L, López-Andarias J, Bornhof AB, Akamatsu M, Sakai N, Matile S, Acc. Chem. Res 2018, 51, 2255–2263. 30188692 10.1021/acs.accounts.8b00223

[23] Kwamen C, Niemeyer J, Chem. Eur. J 2021, 27, 175–186. 32705740 10.1002/chem.202002876 PMC7821015

[24] Kang K, Lohrman JA, Nagarajan S, Chen L, Deng P, Shen X, Fu K, Feng W, Johnson DW, Yuan L, Org. Lett 2019, 21, 652–655. 30638017 10.1021/acs.orglett.8b03778 PMC6653609

[25] Zhao J, Yang D, Yang XJ, Wu B, Coord. Chem. Rev 2019, 378, 415–444.

[26] Yang D, Zhao J, Yang XJ, Wu B, Org. Chem. Front 2018, 5, 662–690.

[27] Hollstein S, Shyshov O, Hanževački M, Zhao J, Rudolf T, Jäger CM, von Delius M, Angew. Chem. Int. Ed 2022, 61, e202201831. 10.1002/anie.202201831 PMC9400851 35384202

[28] Zhang W, Zhao J, Yang D, ChemPlusChem 2022, 87, e202200294. 36410745 10.1002/cplu.202200294

[29] Feng GF, Geng J, Da Feng F, Huang W, Sci. Rep 2020, 10, 2–6. 32170278 10.1038/s41598-020-61813-6 PMC7070053

[30] Zhang G, Mastalerz M, Chem. Soc. Rev 2014, 43, 1934–1947. 24336604 10.1039/c3cs60358j

[31] Thevenet A, Custelcean R, Moyer BA, Jansone-Popova S, Chem. Eur. J 2020, 26, 14290–14294. 32790908 10.1002/chem.202003100

[32] Parks FC, Sheetz EG, Stutsman SR, Lutolli A, Debnath S, Raghavachari K, Flood AH, J. Am. Chem. Soc 2022, 144, 1274–1287. 35015538 10.1021/jacs.1c10758

[33] Massena CJ, Decato DA, Berryman OB, Angew. Chem 2018, 130, 16341–16345. 10.1002/anie.201810415 PMC6449053 30324741

[34] Dhara A, Fadler RE, Chen Y, Köttner LA, Van Craen D, Carta V, Flood AH, Chem. Sci 2023, 14, 2585–2595. 36908961 10.1039/d2sc05121d PMC9993851

[35] Vickers MS, Beer PD, Chem. Soc. Rev 2007, 36, 211–225. 17264924 10.1039/b518077p

[36] Caballero A, Zapata F, Beer PD, Coord. Chem. Rev 2013, 257, 2434–2455.

[37] Heard W, Goldup SM, ACS Cent. Sci 2020, 6, 117–128. 32123730 10.1021/acscentsci.9b01185 PMC7047278

[38] Riebe J, Niemeyer J, Eur. J. Org. Chem 2021, 2021, 5106–5116.

[39] Hübner GM, Gläser J, Seel C, Vögtle F, Angew. Chem. Int. Ed 1999, 38, 383–386. 10.1002/(SICI)1521-3773(19990201)38:3<383::AID-ANIE383>3.0.CO;2-H 29711647

[40] Wisner JA, Beer PD, Drew MGB, Sambrook MR, J. Am. Chem. Soc 2002, 124, 12469–12476. 12381188 10.1021/ja027519a

[41] Sambrook MR, Beer PD, Lankshear MD, Ludlow RF, Wisner JA, Org. Biomol. Chem 2006, 4, 1529–1538. 16604221 10.1039/b518178j

[42] Lim JYC, Bunchuay T, Beer PD, Chem. Eur. J 2017, 23, 4700–4707. 28160507 10.1002/chem.201700030

[43] Ghosh P, Mermagen O, Schalley CA, Chem. Commun 2002, 2, 2628–2629. 10.1039/b208361b 12510270

[44] Fiala T, Sindelar V, Supramol. Chem 2016, 28, 810–816.

[45] Lee S, Chen CH, Flood AH, Nat. Chem 2013, 5, 704–710. 23881503 10.1038/nchem.1668

[46] Qiao B, Liu Y, Lee S, Pink M, Flood AH, Chem. Commun 2016, 52, 13675–13678. 10.1039/c6cc08113d 27812564

[47] Hargrove AE, Nieto S, Zhang T, Sessler JL, Anslyn EV, Chem. Rev 2011, 111, 6603–6782. 21910402 10.1021/cr100242s PMC3212652

[48] Ngo HT, Liu X, Jolliffe KA, Chem. Soc. Rev 2012, 41, 4928–4965. 22688834 10.1039/c2cs35087d

[49] Pal S, Ghosh TK, Ghosh R, Mondal S, Ghosh P, Coord. Chem. Rev 2020, 405, 213128.

[50] Gavette JV, Mills NS, Zakharov LN, Johnson CA, Johnson DW, Haley MM, Angew. Chem 2013, 125, 10460–10464. 10.1002/anie.201302929 PMC3857097 23934912

[51] Wezenberg SJ, Vlatković M, Kistemaker JCM, Feringa BL, J. Am. Chem. Soc 2014, 136, 16784–16787. 25402836 10.1021/ja510700j

[52] Rice R, Vacchina P, Norris-Mullins B, Morales MA, Smith BD, Antimicrob. Agents Chemother 2016, 60, 2932–2940. 26926632 10.1128/AAC.00410-16 PMC4862449

[53] Kyung KIMS, Dong Hoon LEE, Hong JIN, Yoon J, Acc. Chem. Res 2009, 42, 23–31. 18798656 10.1021/ar800003f

[54] Lee S, Yuen KKY, Jolliffe KA, Yoon J, Chem. Soc. Rev 2015, 44, 1749–1762. 25578599 10.1039/c4cs00353e

[55] Sokkalingam P, Kim DS, Hwang H, Sessler JL, Lee CH, Chem. Sci 2012, 3, 1819–1824.

[56] Ali M, Ahmed I, Ramirez P, Nasir S, Niemeyer CM, Mafe S, Ensinger W, Small 2016, 12, 2014–2021. 26939057 10.1002/smll.201600160

[57] Jolliffe KA, Acc. Chem. Res 2017, 50, 2254–2263. 28805368 10.1021/acs.accounts.7b00252

[58] Ma C, Lin C, Wang Y, Chen X, TrAC Trends Anal. Chem 2016, 77, 226–241.

[59] Fujita K, Fujiwara S, Yamada T, Tsuchido Y, Hashimoto T, Hayashita T, J. Org. Chem 2017, 82, 976–981. 27997800 10.1021/acs.joc.6b02513

[60] Sheetz G, Zhang Z, Marogil A, Che M, Pink M, Carta V, Raghavachari K, Flood AH, Chem. Eur. J 2022, 28, e202201584. 35754003 10.1002/chem.202201584

[61] Fadler RE, Al Ouahabi A, Qiao B, Carta V, Niklas FK, Gao X, J. Org. Chem 2021, 86, 4532–4546. 33636075 10.1021/acs.joc.0c02887 PMC8063573

[62] Zhao W, Tropp J, Qiao B, Pink M, Azoulay JD, Flood AH, J. Am. Chem. Soc 2020, 142, 2579–2591. 31931561 10.1021/jacs.9b12645

[63] Sun J, Vogel J, Chen L, Schleper AL, Bergner T, Kuehne AJC, von Delius M, Chem. Eur. J 2022, 28, e202104116. 35038189 10.1002/chem.202104116 PMC9303926

[64] Englert A, Vogel JF, Bergner T, Loske J, Von Delius M, J. Am. Chem. Soc 2022, 144, 15266–15274. 35953065 10.1021/jacs.2c05861 PMC9413217

[65] Borsley S, Leigh DA, Roberts BMW, Nat. Chem 2022, 14, 728–738. 35778564 10.1038/s41557-022-00970-9

[66] Singh N, Formon GJM, De Piccoli S, Hermans TM, Adv. Mater 2020, 32, 1906834. 10.1002/adma.201906834 32064688

[67] Das K, Gabrielli L, Prins LJ, Angew. Chem. Int. Ed 2021, 60, 20120–20143. 10.1002/anie.202100274 PMC8453758 33704885

[68] Van Rossum SAP, Tena-Solsona M, Van Esch JH, Eelkema R, Boekhoven J, Chem. Soc. Rev 2017, 46, 5519–5535. 28703817 10.1039/c7cs00246g

[69] De S, Klajn R, Adv. Mater 2018, 30, 1706750. 10.1002/adma.201706750 29520846

[70] Zhao W, Qiao B, Chen CH, Flood AH, Angew. Chem. Int. Ed 2017, 56, 13083–13087. 10.1002/anie.201707869 28833990

[71] Fatila M, Pink M, Twum EB, Karty JA, Flood AH, Chem. Sci 2018, 9, 2863–2872. 29780454 10.1039/c7sc05290a PMC5941797

[72] Zhao W, Qiao B, Tropp J, Pink M, Azoulay JD, Flood AH, J. Am. Chem. Soc 2019, 141, 4980–4989. 30789722 10.1021/jacs.9b00248

[73] Zhao W, Flood AH, White NG, Chem. Soc. Rev 2020, 49, 7893–7906. 32677649 10.1039/d0cs00486c

[74] Qiao B, Anderson JR, Pink M, Flood AH, Chem. Commun 2016, 52, 8683–8686. 10.1039/c6cc03463b 27331606

[75] Xue M, Yang Y, Chi X, Yan X, Huang F, Chem. Rev 2015, 115, 7398–7501. 25734835 10.1021/cr5005869

[76] McConnell AJ, Beer PD, Chem. Eur. J 2011, 17, 2724–2733. 21264965 10.1002/chem.201002528

[77] Affeld A, Hühner GM, Seel C, Schalley CA, Eur. J. Org. Chem 2001, 15, 2877–2890.

[78] Erbas-Cakmak S, Leigh DA, McTernan CT, Nussbaumer AL, Chem. Rev 2015, 115, 10081–10206. 26346838 10.1021/acs.chemrev.5b00146 PMC4585175

[79] Leigh DA, Wong JKY, Dehez F, Zerbetto F, Nature 2003, 424, 174–179. 12853952 10.1038/nature01758

[80] Coskun A, Banaszak M, Astumian RD, Stoddart JF, Grzybowski BA, Chem. Soc. Rev 2012, 41, 19–30. 22116531 10.1039/c1cs15262a

[81] Bruns CJ, Nat. Nanotechnol 2022, 17, 1231–1234. 36494473 10.1038/s41565-022-01247-5

[82] Aprahamian I, ACS Cent. Sci 2020, 6, 347–358. 32232135 10.1021/acscentsci.0c00064 PMC7099591

[83] Cheng C, McGonigal PR, Schneebeli ST, Li H, Vermeulen NA, Ke C, Stoddart JF, Nat. Nanotechnol 2015, 10, 547–553. 25984834 10.1038/nnano.2015.96

[84] Qiu Y, Zhang L, Pezzato C, Feng Y, Li W, Nguyen MT, Cheng C, Shen D, Guo QH, Shi Y, Cai K, Alsubaie FM, Astumian RD, Stoddart JF, J. Am. Chem. Soc 2019, 141, 17472–17476. 31622089 10.1021/jacs.9b08927

[85] Zhang L, Qiu Y, Liu WG, Chen H, Shen D, Song B, Cai K, Wu H, Jiao Y, Feng Y, Seale JSW, Pezzato C, Tian J, Tan Y, Chen XY, Guo QH, Stern CL, Philp D, Astumian RD, Goddard III WA, Stoddart JF, Nature 2023, 613, 280–286. 36631649 10.1038/s41586-022-05421-6 PMC9834048

[86] Corra S, Bakić MT, Groppi J, Baroncini M, Silvi S, Penocchio E, Esposito M, Credi A, Nat. Nanotechnol 2022, 17, 746–751. 35760895 10.1038/s41565-022-01151-y

[87] The free energy of hydration of phosphates is reported to be relatively high, compared to other anions.^[88]^ It is likely that this is also true for pyrophosphonates. We found that addition of water can completely shut down the CS:PP binding (Figure S69) due to competitive solvation of the PP dianions. We therefore dried all substances under vacuum prior to use and performed sample preparation under exclusion of moisture.

[88] Marcus Y, J. Chem. Soc. Faraday Trans 1993, 89, 2995–2999.

[89] Eliel EL, Wilen SH, Mander LN, Stereochemistry of Organic Compounds, John Wiley&Sons, New York 1994.

[90] Solel E, Ruth M, Schreiner PR, J. Am. Chem. Soc 2021, 143, 20837–20848. 34846890 10.1021/jacs.1c09222

[91] Solel E, Ruth M, Schreiner PR, J. Org. Chem 2021, 86, 7701–7713. 33988377 10.1021/acs.joc.1c00767

[92] Fatila EM, Twum EB, Sengupta A, Pink M, Karty JA, Raghavachari K, Flood AH, Angew. Chem. Int. Ed 2016, 55, 14057–14062. 10.1002/anie.201608118 27712022

