# Supplementary material for: Steric Control over the Threading of Pyrophosphonates with One or Two Cyanostar Macrocycles during Pseudorotaxane Formation
Source: Chemistry. Author manuscript; Available in PMC 2024 Jul 20. (PMC10655069; doi:10.1002/chem.202300899)
Supplement: Supporting Information [file NIHMS1925964-supplement-Supporting_Information.pdf]

// Chemistry–A European Journal

Supporting Information

Steric Control over the Threading of Pyrophosphonates with One or Two Cyanostar Macrocycles during Pseudorotaxane Formation

Julian Vogel, Yusheng Chen, Rachel E. Fadler, Amar H. Flood,* and Max von Delius*

Table of Contents

General Methods 3

Synthesis 4

NMR Kinetic Experiments 47

NMR Variable Temperature (VT) Experiments 50

^{1}H NMR Titrations 54

NOESY NMR spectrum of [2]pseudorotaxane 57

Mass Spectrometry (MS) Experiments 58

^{31}P NMR titrations and binding constants 60

DFT calculations 66

References 77

General Methods

All reagents were obtained from commercial suppliers (Merck Sigma Aldrich, TCI, AcrosOrganics, FisherScientific or VWR) and used as received.

Phosphonate deprotection,[1] formation of TBA salts,[2] and cyanostar synthesis[3] were performed according to literature procedures with some modifications.

Reversed-phase chromatography was performed on an Advion Interchim Scientific Puriflash 430, using HPLC-grade solvents and an Interchim PF-15C18HP-F0025 C18 column. For lyophilization a Christ Alpha 3-4 LSCbasic was used.

Nuclear magnetic resonance (NMR) spectra were recorded on BrukerAvance 400 or 600 NEO spectrometers (^{1}H: 400 or 600 Hz, ^{13}C: 101 Hz, ^{31}P: 162 or 243 Hz) at room temperature (298 K). Chemical shifts were referenced on residual solvent peaks ($CDCl_3$: 7.26 ppm, $C_2D_2Cl_4$: 6.00 ppm, DCM-d_2: 5.32 ppm, DMSO-d_6: 2.50 ppm). ^{31}P-NMR chemicals shifts were referenced on 85% H_3PO_4 (0.00 ppm). Chemical shifts (δ) are denoted in ppm and coupling constants (*J*) in Hz. Peak multiplicities are indicated as s = singlet, d = doublet, t = triplet and m = multiplet.

High resolution electrospray ionization mass spectroscopy (HRMS – ESI) was performed on an Agilent 1260 Infinity II system with a 6546 LC/QTOF mass spectrometer.

Materials for all studies were dried at least 24 h under vacuum and the sample preparation was performed in a MBraun glovebox to exclude moisture. Deuterated solvents were dried over 3 Å molecular sieves for at least three days prior to use. All NMR studies were performed on a BrukerAvance 600 NEO spectrometer.

The following abbreviations were used: CS: Cyanostar, DFT: Density Functional Theory, DIC: *N,N*-diisopropylcarbodiimide, DCM: dichloromethane, DMSO: dimethylsulfoxide, ESI: electrospray ionization, HPLC: high-performance liquid chromatography, HRMS: high-resolution mass spectrometry, MeCN: acetonitrile, MPLC: medium-performance liquid chromatography, NMR: nuclear magnetic resonance, NOESY: Nuclear Overhauser effect spectroscopy, PP: pyrophosphonate, TBA: tetrabutylammonium, VT: variable temperature.

Synthesis

Synthesis overview

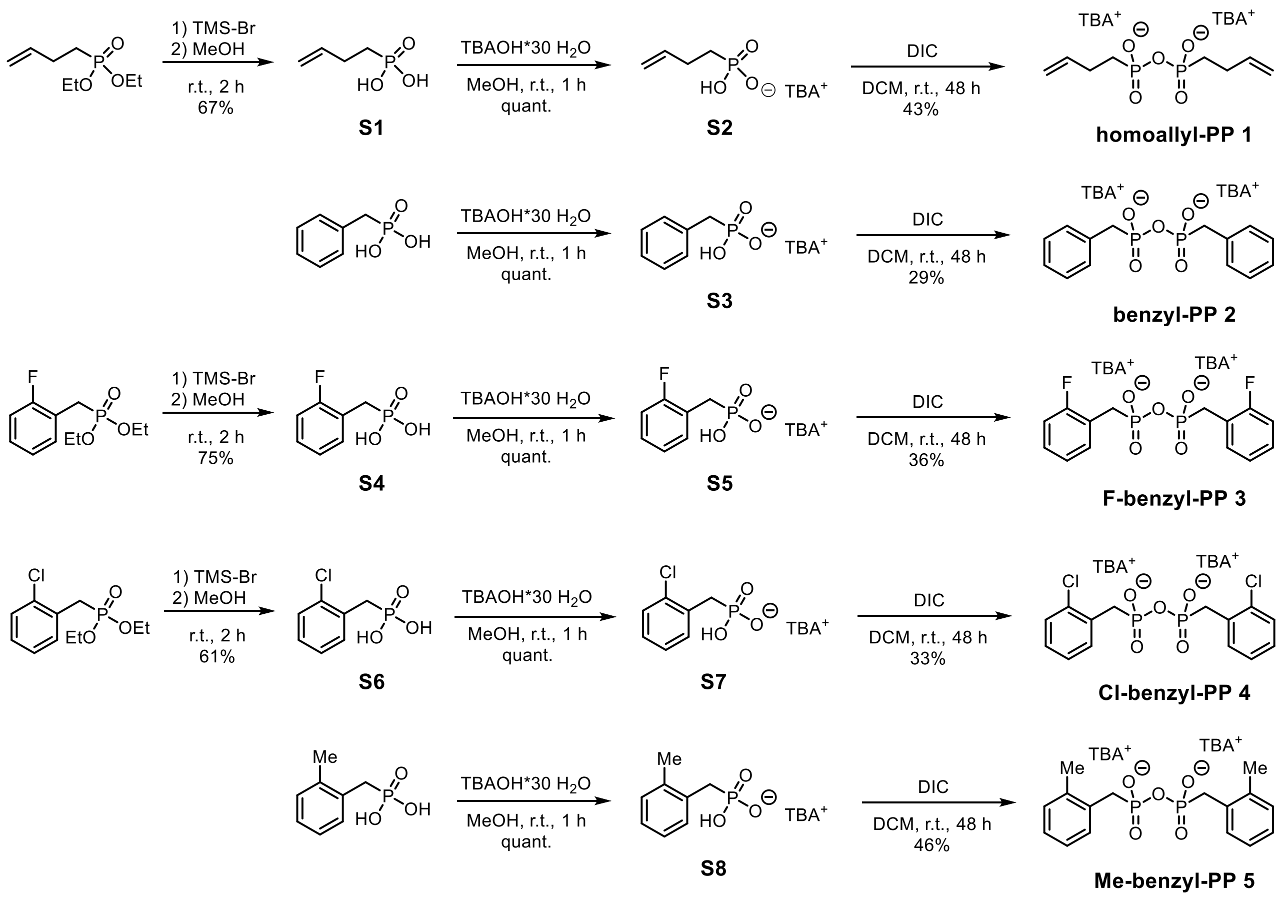

Scheme S1: Synthesis overview.

Synthesis of 3-butenylphosphonic acid S1

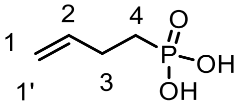

S1

1.00 mL (5.20 mmol) diethyl 3-butenylphosphonate were dissolved in 50 mL anhydrous DCM and 6.91 mL (52.0 mmol, 10 equiv.) trimethylsilylbromide were added. The mixture was stirred for 2 h at room temperature under inert atmosphere until TLC (DCM, 5% MeOH, stained with $KMnO_4$) indicated full conversion. DCM was evaporated and the residue was stirred for 1 h in anhydrous MeOH before the solvent was removed. MPLC on an Interchim PF-15C18HP-F0025 C18 column with a mobile phase of MeCN and 25 mM formic acid (5-10% MeCN) and subsequent lyophilization yielded 474 mg (3.48 mmol, 67%) of the desired product as a colourless solid.

^{1}H-NMR (400 MHz, DMSO-d$_6$): δ [ppm] = 5.92-5.82 (m, 1 H, **2**), 5.03 (dd, ^{3}J = 17.2 Hz, ^{2}J = 1.68 Hz, 1 H, **1'**), 4.94 (dd, ^{3}J = 10.2 Hz, ^{2}J = 1.40 Hz, 1 H, **1**), 2.20 (m, 2 H, **3**), 1.59 (m, 2 H, **4**).

^{13}C-NMR (101 MHz, DMSO-d$_6$): δ [ppm] = 138.4 (d), 114.7, 27.0 (d), 26.8 (d).

^{31}P-NMR (162 MHz, DMSO-d$_6$, external standard: 85% H_3PO_4): δ [ppm] = 27.30.

HRMS(ESI (-)): m/z = 135.02179 (calc. 135.02165, δm/m = 1.04 ppm) $[M-H]^-$

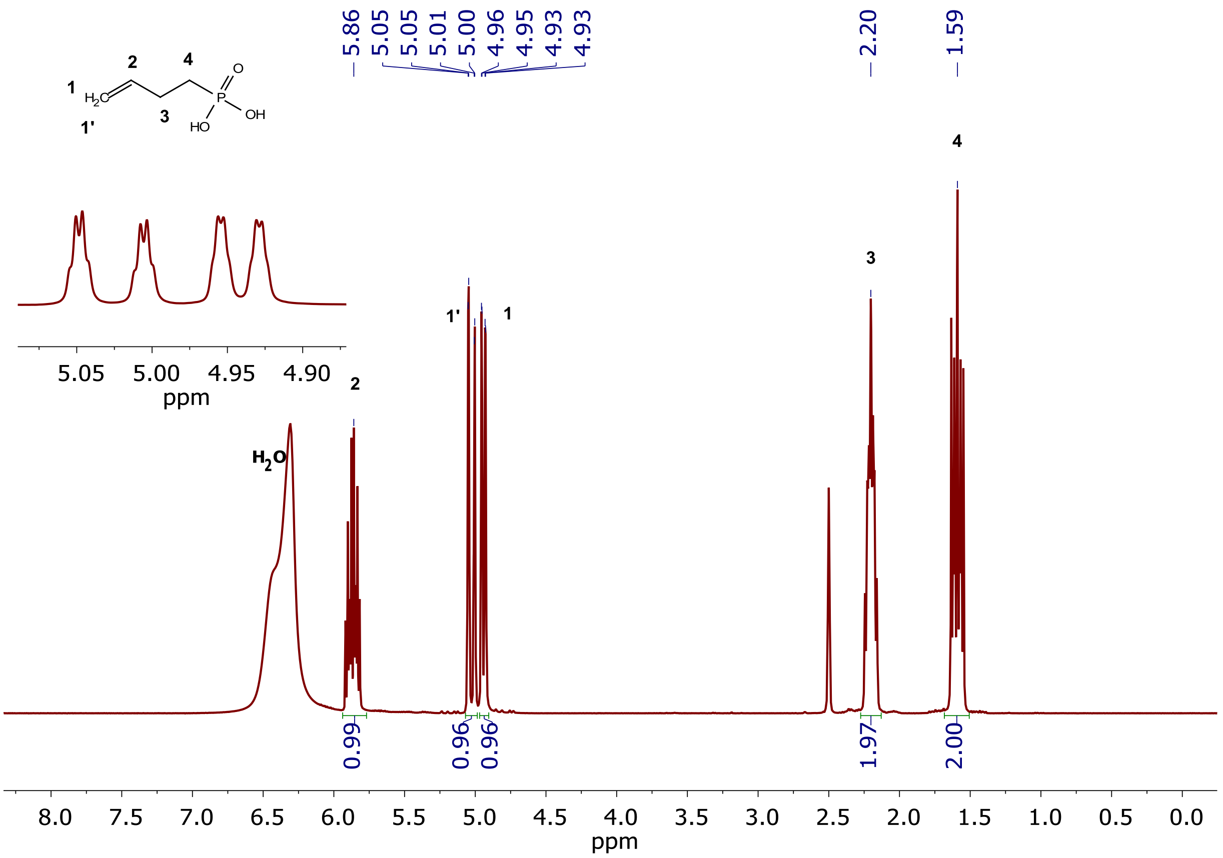

Figure S1: ^{1}H NMR (400 MHz, DMSO-d$_6$, 298 K) of 3-butenylphosphonic acid **S1**.

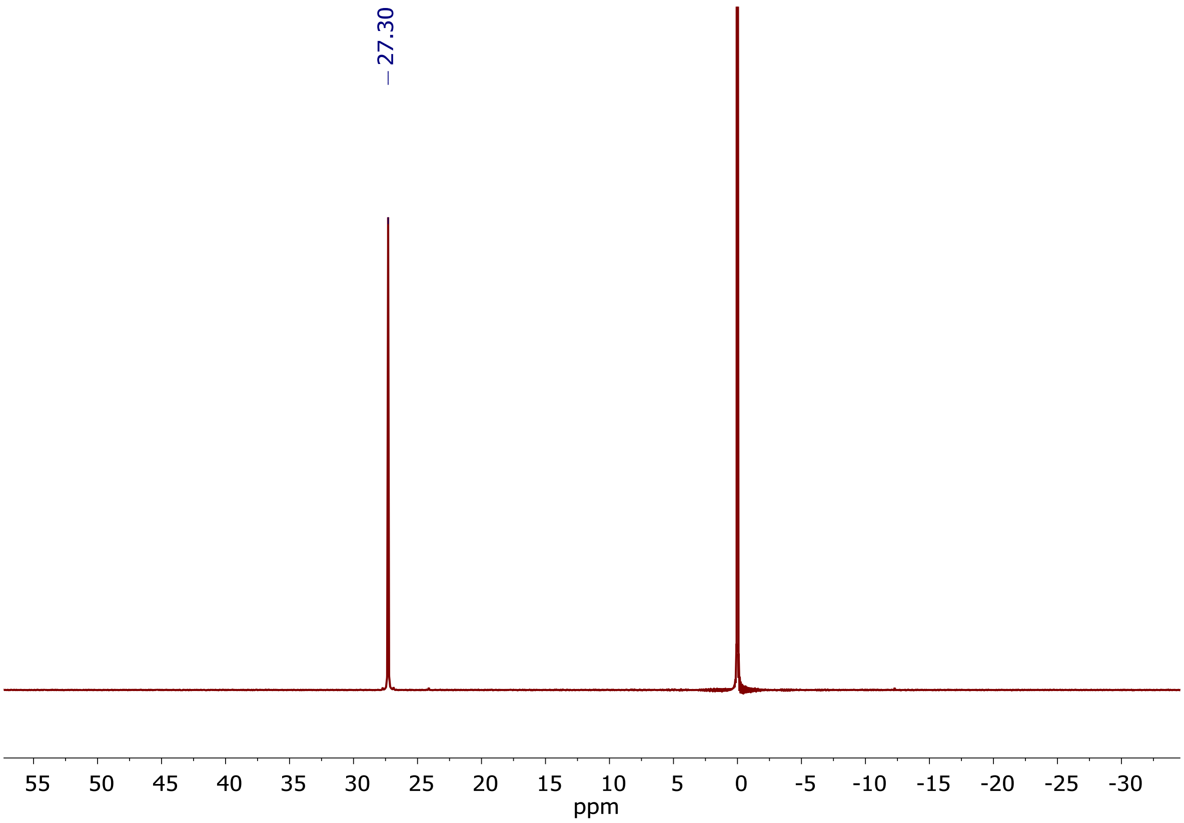

Figure S2: ^{31}P NMR (162 MHz, DMSO-d$_6$, 298 K, 85% H_3PO_4) of 3-butenylphosphonic acid **S1**.

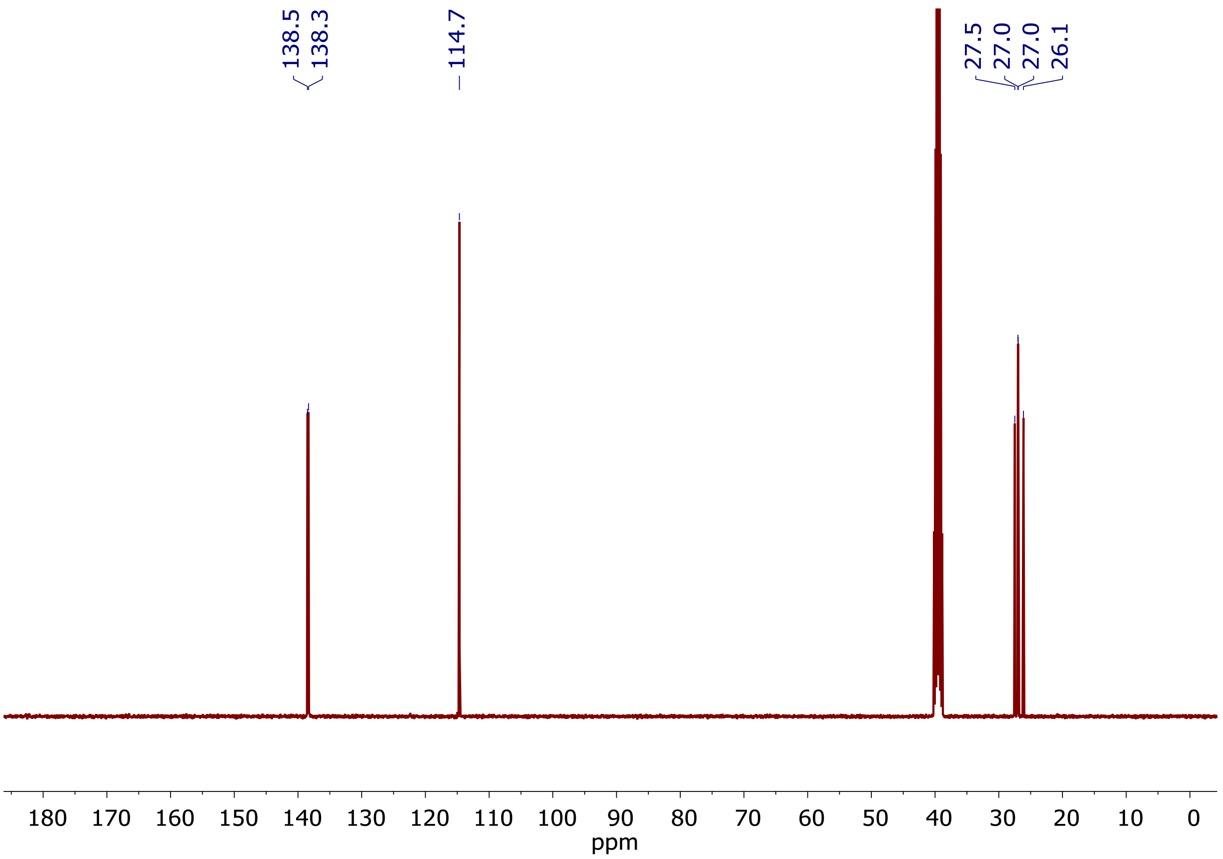

Figure S3: ^{13}C NMR (101 MHz, DMSO-d_6, 298 K) of 3-butenylphosphonic acid **S1**.

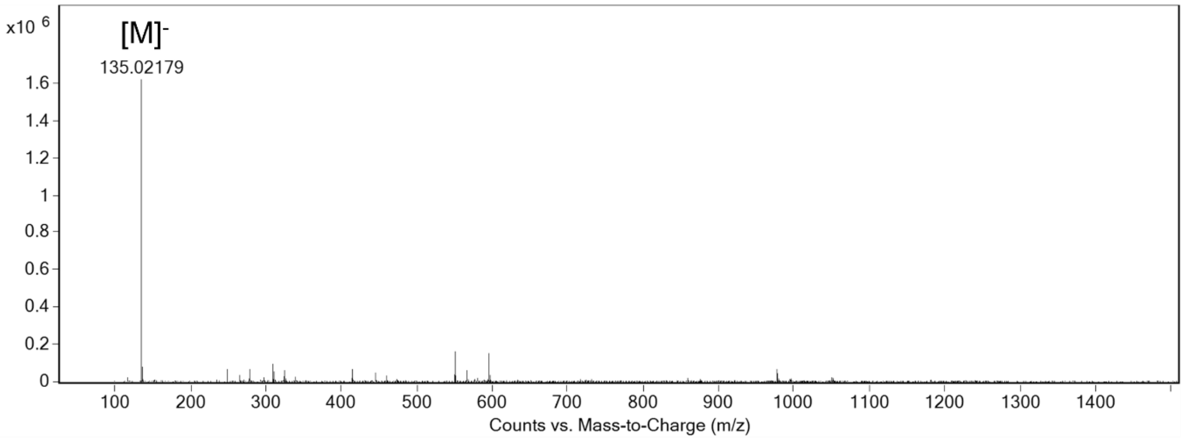

Figure S4: HRMS (ESI, negative mode) of 3-butenylphosphonic acid **S1**.

Synthesis of tetrabutylammonium 3-butenyl phosphonate S2

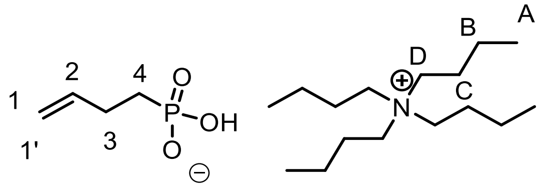

S2

450 mg (3.30 mmol) 3-butenylphosphonic acid **S1** and 2.64 g (3.30 mmol, 1.0 equiv.) TBAOH · 30 H_2O were combined in a round bottom flask and dissolved in 40 mL MeOH. The mixture was stirred for 1 h at room temperature, before the solvent was removed and the product was dried in vacuo to afford 1.25 g (3.30 mmol, quant.) of the desired TBA salt as a colourless solid.

^{1}H-NMR (400 MHz, $CDCl_3$): δ [ppm] = 5.93-5.82 (m, 1 H, **2**), 4.90 (dd, ^{3}J = 17.2 Hz, ^{2}J = 1.83 Hz, 1 H, **1'**), 4.74 (d_{br}, ^{3}J = 10.8 Hz, 1 H, **1**), 3.31-3.27 (m, 8 H, **D**), 2.35 (m, 2 H, **3**), 1.63-1.58 (m, 8 H, **C**), 1.53 (m, 2 H, **4**), 1.45-1.36 (m, 8 H, **B**), 0.93 (t, ^{3}J = 7.36 Hz, 12 H, **A**).

^{13}C-NMR (101 MHz, $CDCl_3$): δ [ppm] = 141.4 (d), 112.3, 58.7, 29.6 (d), 29.4 (d), 24.2, 19.8, 13.8.

^{31}P-NMR (162 MHz, $CDCl_3$, external standard: 85% H_3PO_4): δ [ppm] = 21.78.

HRMS(ESI (-)): m/z = 135.02236 (calc. 135.02165, δm/m = 5.26 ppm) $[M]^-$, 512.32897 (calc. 512.32753, δm/m = 2.81 ppm) $[2M+TBA]^-$.

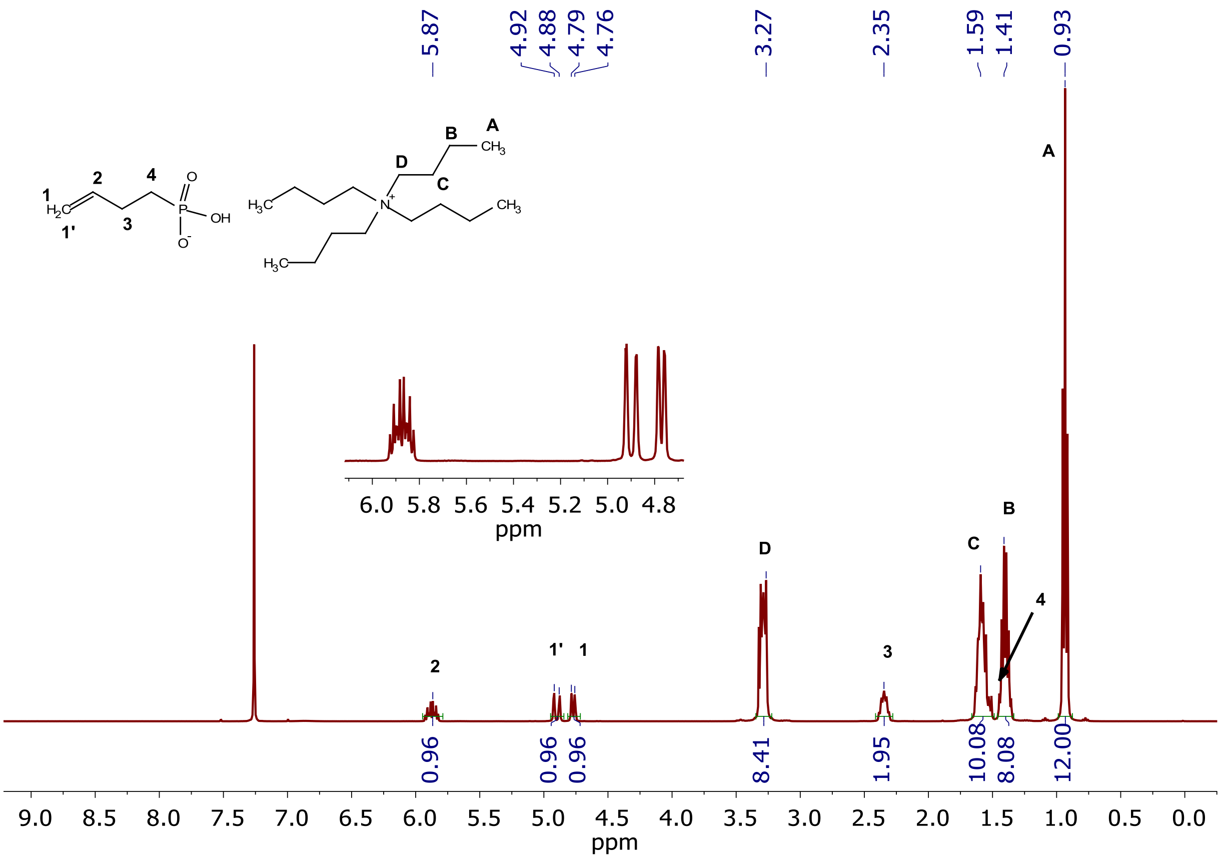

Figure S5: ^{1}H NMR (400 MHz, $CDCl_3$, 298 K) of tetrabutylammonium 3-butenyl phosphonate **S2**.

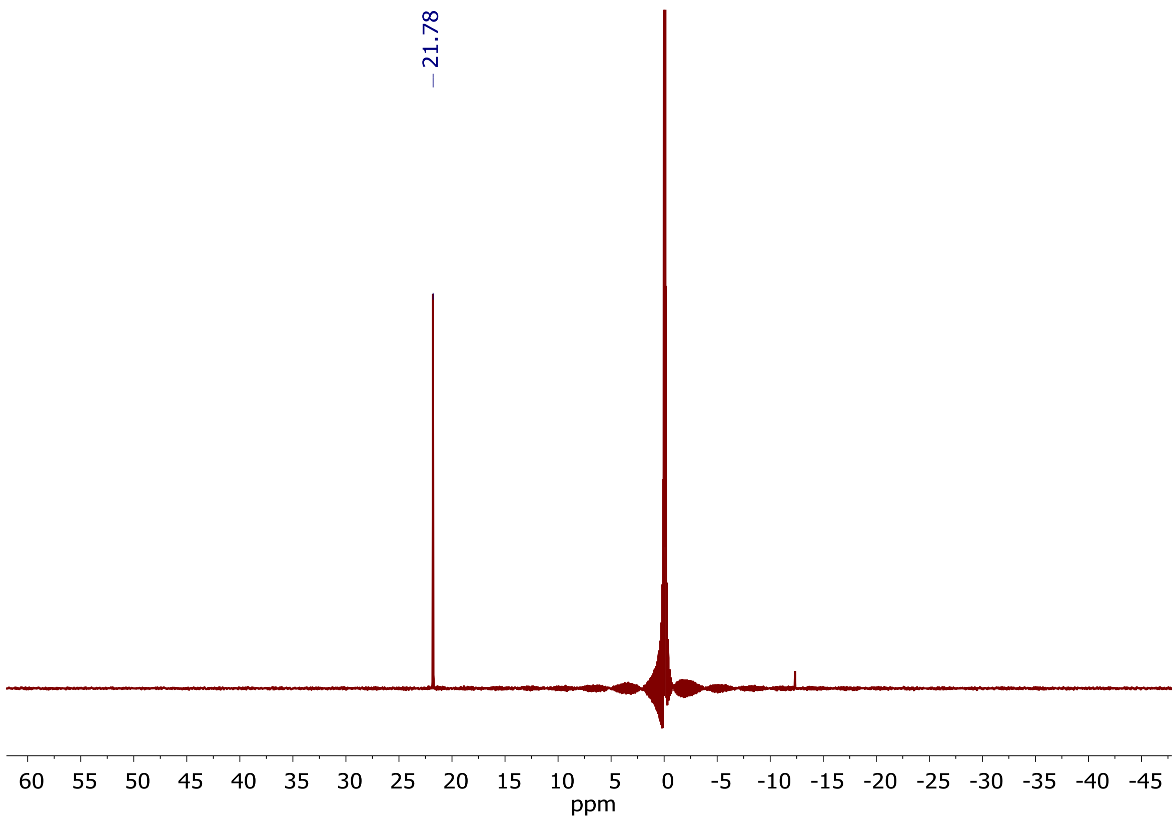

Figure S6: ^{31}P NMR (162 MHz, $CDCl_3$, 298 K, 85% H_3PO_4) of tetrabutylammonium 3-butenyl phosphonate **S2**. Peak at -12.6 ppm, belongs to an impurity in the internal standard.

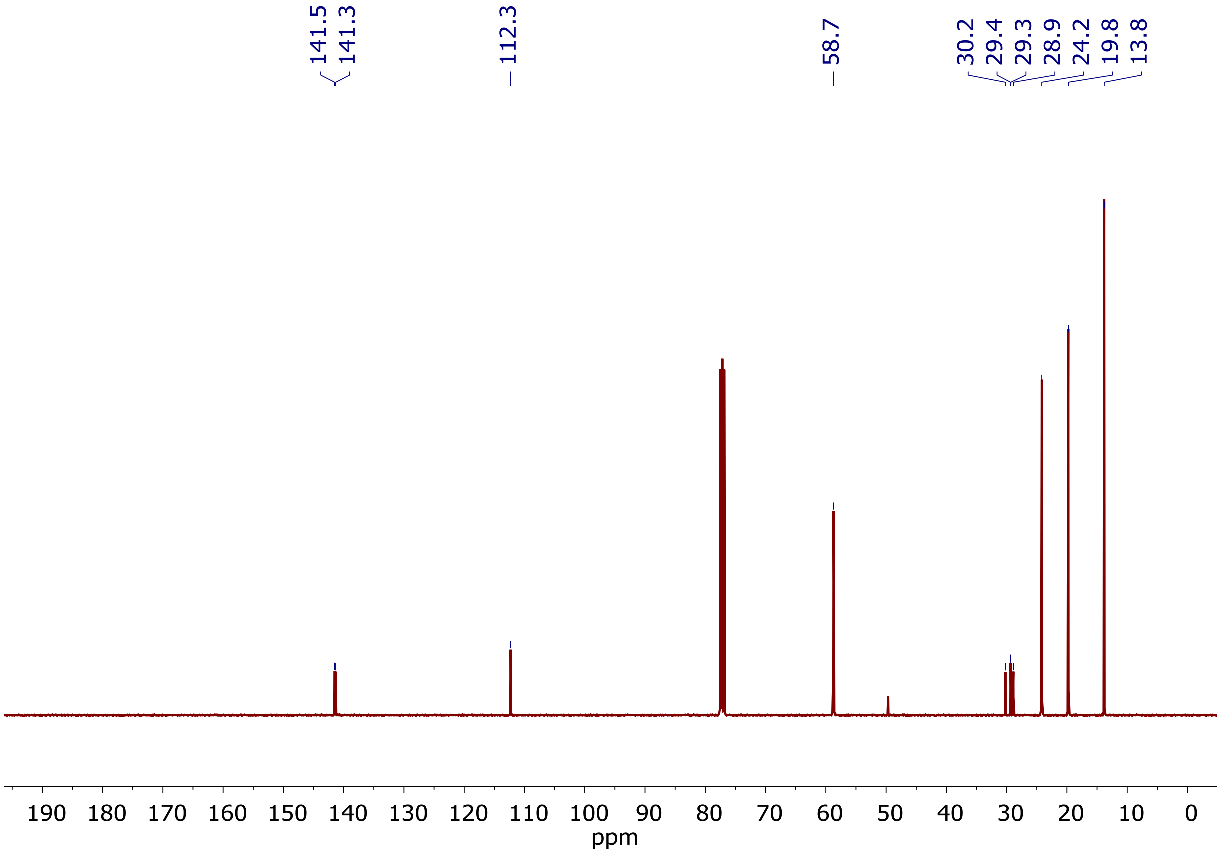

Figure S7: ^{13}C NMR (101 MHz, $CDCl_3$, 298 K) of tetrabutylammonium 3-butenyl phosphonate **S2**.

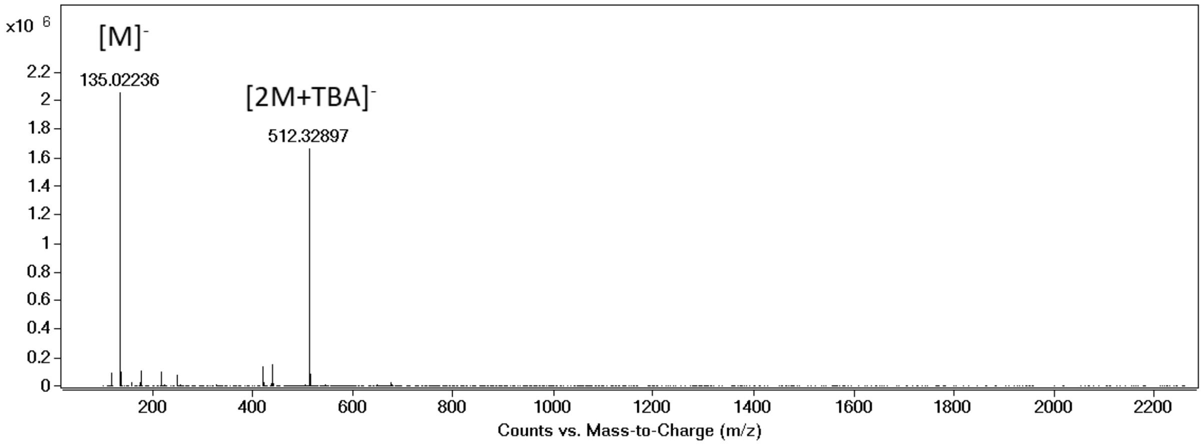

Figure S8: HRMS (ESI, negative mode) of tetrabutylammonium 3-butenyl phosphonate **S2**.

Synthesis of *bis*-tetrabutylammonium 3-butenyl pyrophosphonate (homoallyl-PP) 1

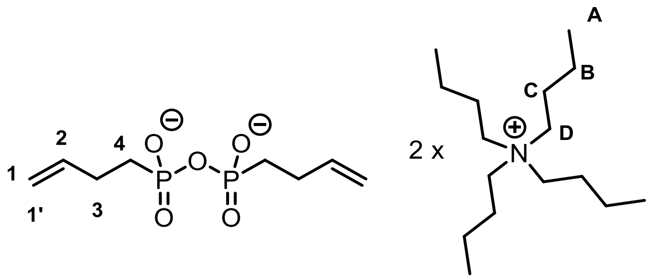

1

350 mg (0.93 mmol) tetrabutylammonium 3-butenyl phosphonate **S2** were dissolved in 40 mL DCM and 1.41 mL (9.30 mmol, 10 equiv.) *N,N'*-diisopropylcarbodiimide (DIC) were added. The mixture was stirred at room temperature for 48 h. DCM was evaporated and the residue was dissolved in 4 mL H_2O / MeCN (3:1) mixture. MPLC on an Interchim PF-15C18HP-F0025 C18 column with a mobile phase of MeCN and water (20-30% MeCN) and subsequent lyophilization yielded 150 mg (0.20 mmol, 43%) of the dimer as a colourless oil.

^{1}H-NMR (400 MHz, $CDCl_3$): δ [ppm] = 5.94-5.85 (m, 2 H, **2**), 4.95 (dd, ^3J = 17.1 Hz, ^2J = 1.85 Hz, 2 H, **1'**), 4.82 (d_{br}, ^3J = 10.2 Hz, 2 H, **1**), 3.39-3.33 (m, 16 H, **D**), 2.39 (m, 4 H, **3**), 1.74 (m, 4 H, **4**), 1.63-1.59 (m, 16 H, **C**), 1.47-1.39 (m, 16 H, **B**), 0.96 (t, ^3J = 7.30 Hz, 24 H, **A**).

^{13}C-NMR (101 MHz, $CDCl_3$): δ [ppm] = 141.1 (t), 112.6, 58.7, 30.1 (d), 28.9 (d), 24.2, 19.8, 13.8.

^{31}P-NMR (162 MHz, $CDCl_3$, external standard: 85% H_3PO_4): δ [ppm] = 13.64.

HRMS(ESI (-)): m/z = 253.04108 (calc. 253.04002, δm/m = 4.19 ppm) $[M]^-$, 494.31733 (calc. 494.31697, δm/m = 0.73 ppm) $[2M+TBA]^-$.

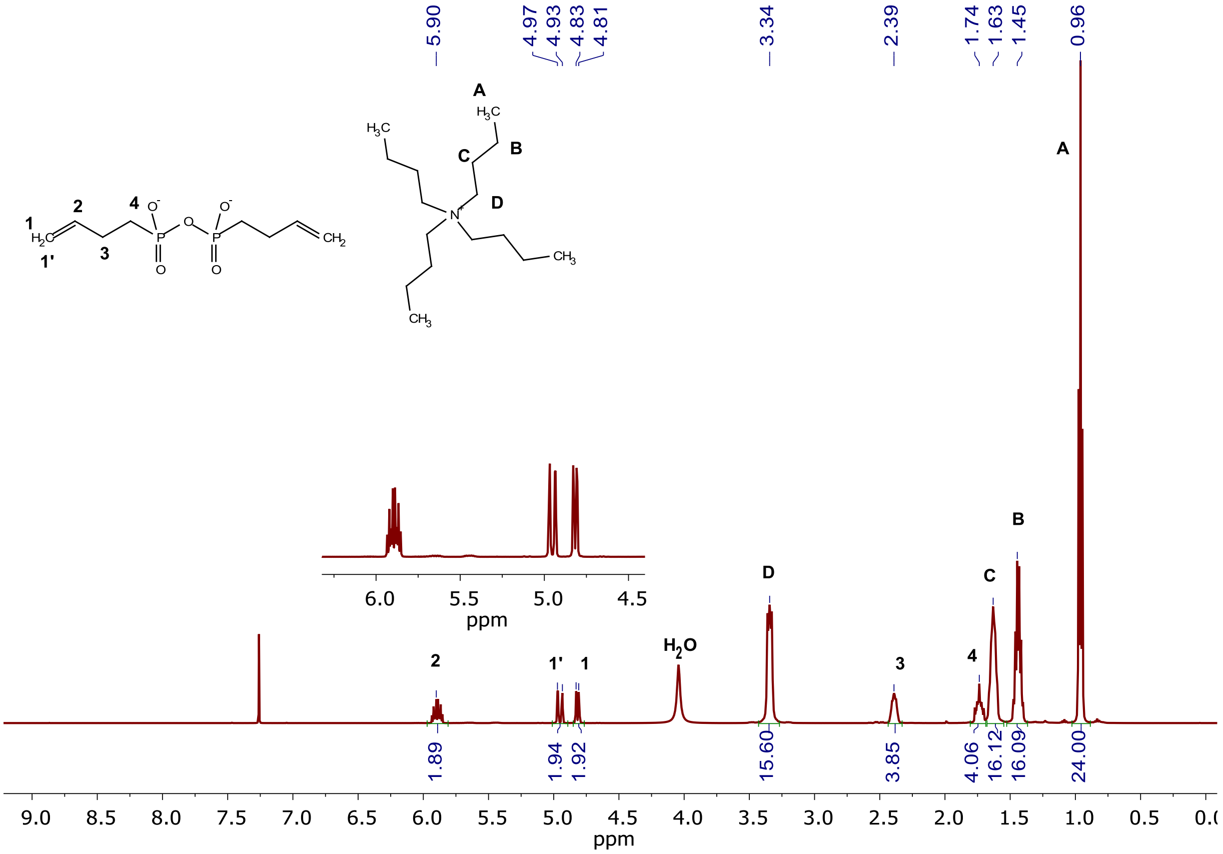

Figure S9: ^{1}H NMR (400 MHz, $CDCl_3$, 298 K) of *bis*-tetrabutylammonium 3-butenyl pyrophosphonate **1**.

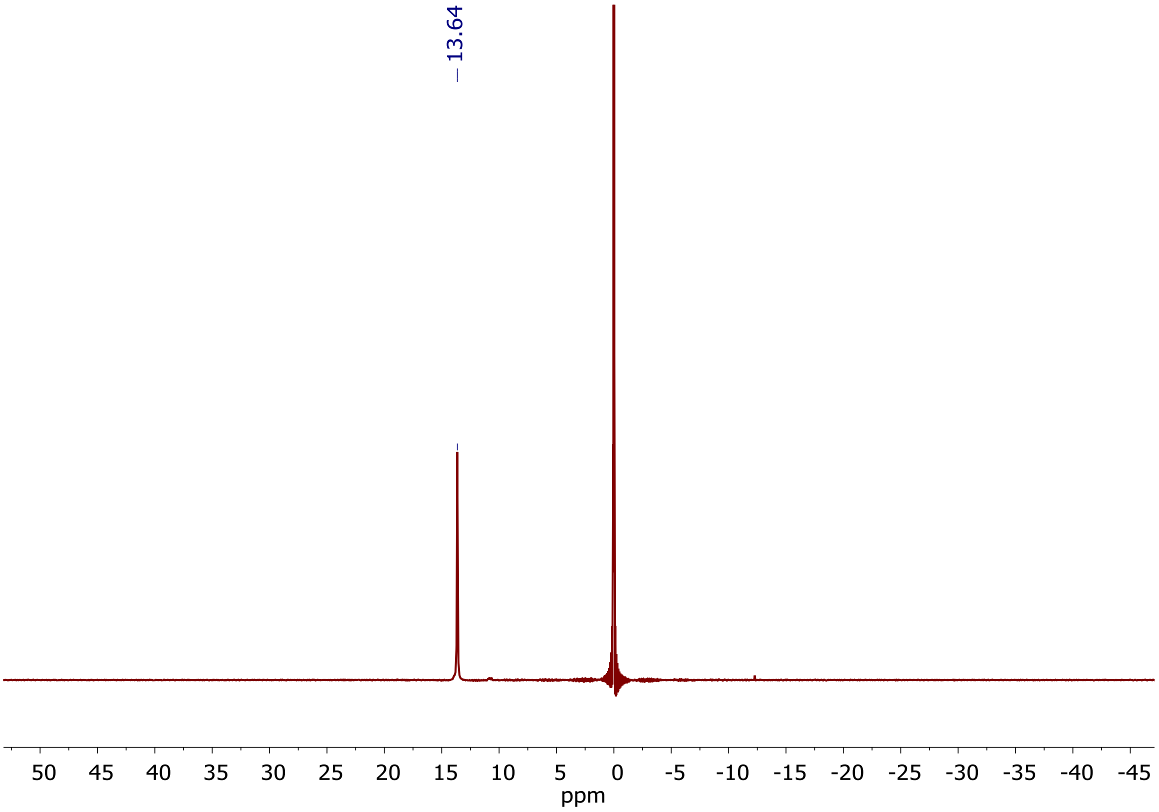

Figure S10: ^{31}P NMR (162 MHz, $CDCl_3$, 298 K, 85% H_3PO_4) of *bis*-tetrabutylammonium 3-butenyl pyrophosphonate **1**.

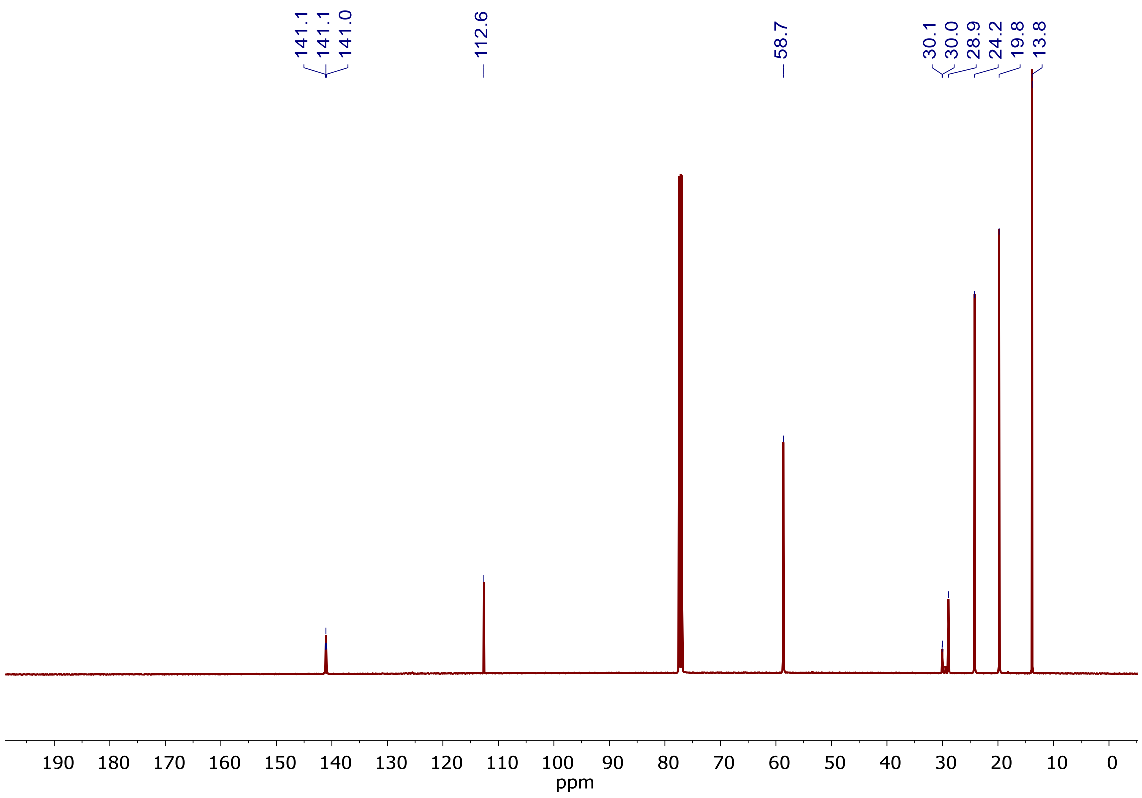

Figure S11: ^{13}C NMR (101 MHz, $CDCl_3$, 298 K) of *bis*-tetrabutylammonium 3-butenyl pyrophosphonate **1**.

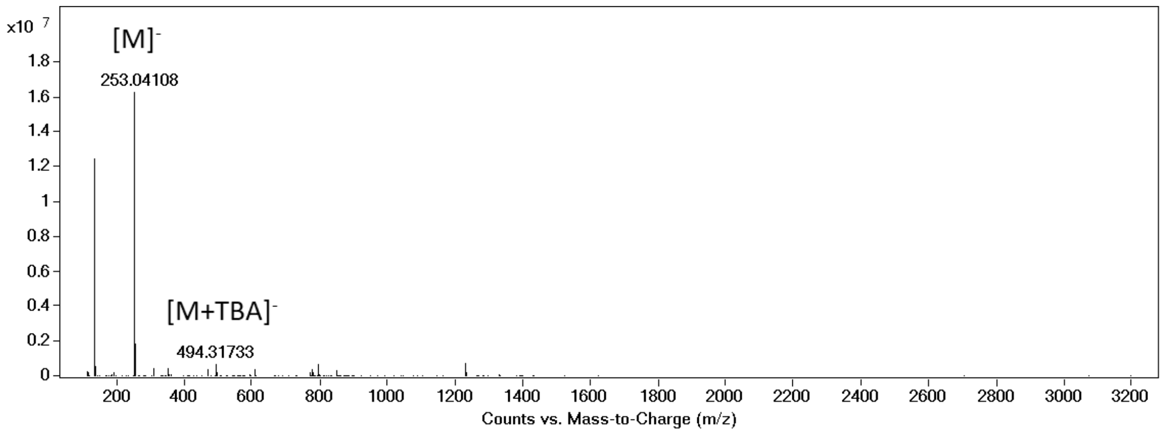

Figure S12: HRMS (ESI, negative mode) of *bis*-tetrabutylammonium 3-butenyl pyrophosphonate **1**.

Synthesis of tetrabutylammonium benzyl phosphonate S3

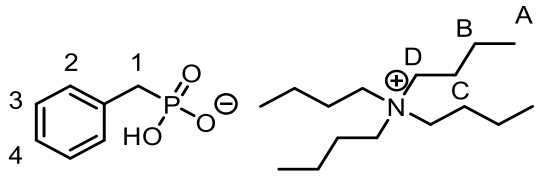

S3

200 mg (1.16 mmol) benzylphosphonic acid and 928 mg (1.16 mmol, 1.0 equiv.) TBAOH · 30 H_2O were combined in a round bottom flask and dissolved in 12 mL MeOH. The mixture was stirring for 1 h at room temperature, before the solvent was removed and the product was dried in vacuo to afford 483 mg (1.16 mmol, quant.) of the desired TBA salt as a colourless solid.

^{1}H-NMR (400 MHz, $CDCl_3$): δ [ppm] = 7.42-7.36 (m, 2 H, **3**), 7.16 (t, ^{3}J = 7.59 Hz, 2 H, **2**), 7.08-7.04 (dt, ^{3}J = 7.48 Hz, ^{4}J = 1.36 Hz, 1 H, **4**), 3.20-3.09 (m, 8 H, **D**), 2.93 (d, $^{2}J_{H-P}$ = 20.7 Hz, 2 H, **1**), 1.60-1.44 (m, 8 H, **C**), 1.42-1.30 (m, 8 H, **B**), 0.95 (t, ^{3}J = 7.35 Hz, 12 H, **A**).

^{13}C-NMR (101 MHz, $CDCl_3$): δ [ppm] = 138.4 (d), 130.3 (d), 127.6 (d), 124.7 (d), 58.8, 37.8 (d), 24.2, 19.8, 13.9.

^{31}P-NMR (162 MHz, $CDCl_3$, external standard: 85% H_3PO_4): δ [ppm] = 16.71.

HRMS(ESI (-)): m/z = 171.02168 (calc. 171.02165, δm/m = 0.18 ppm) $[M]^-$, 584.32771 (calc. 584.32754, δm/m = 0.34 ppm) $[2M+TBA]^-$.

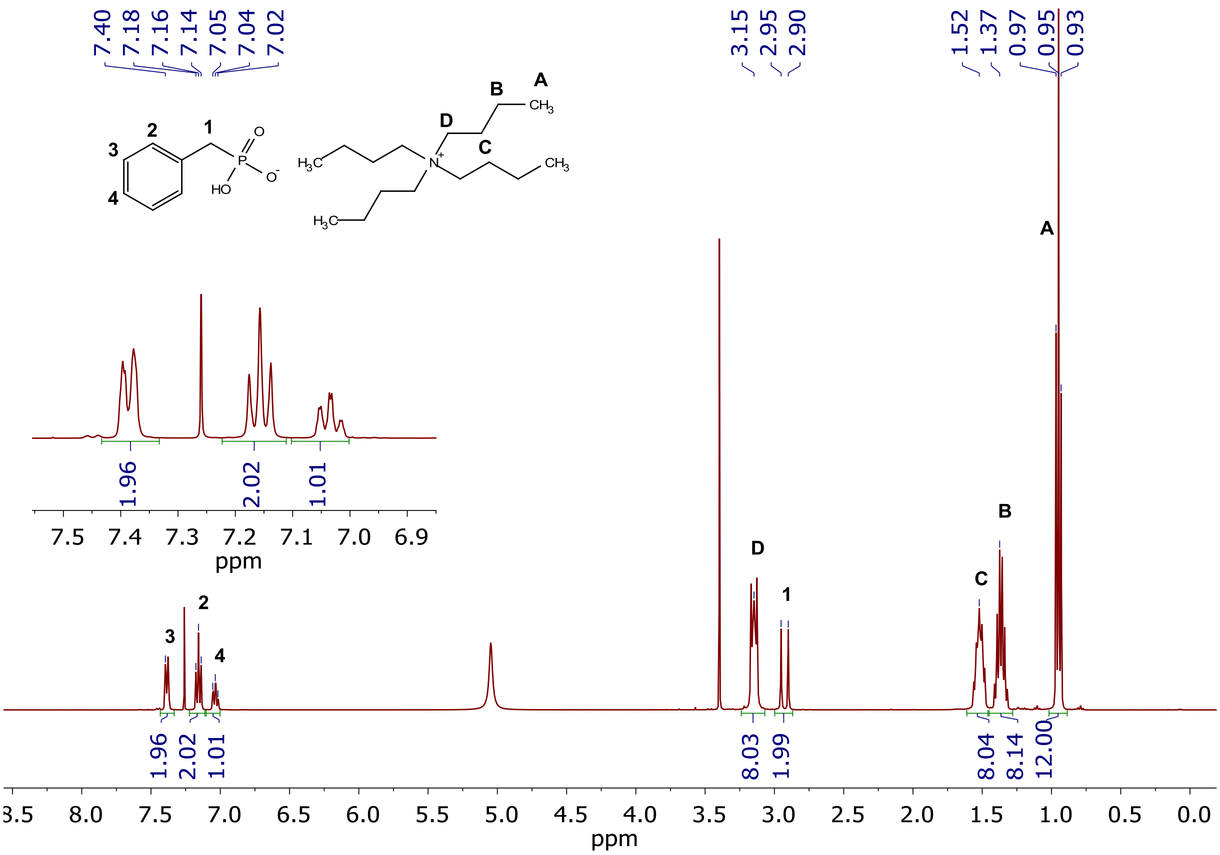

Figure S13: ^{1}H NMR (400 MHz, $CDCl_3$, 298 K) of tetrabutylammonium benzyl phosphonate **S3**.

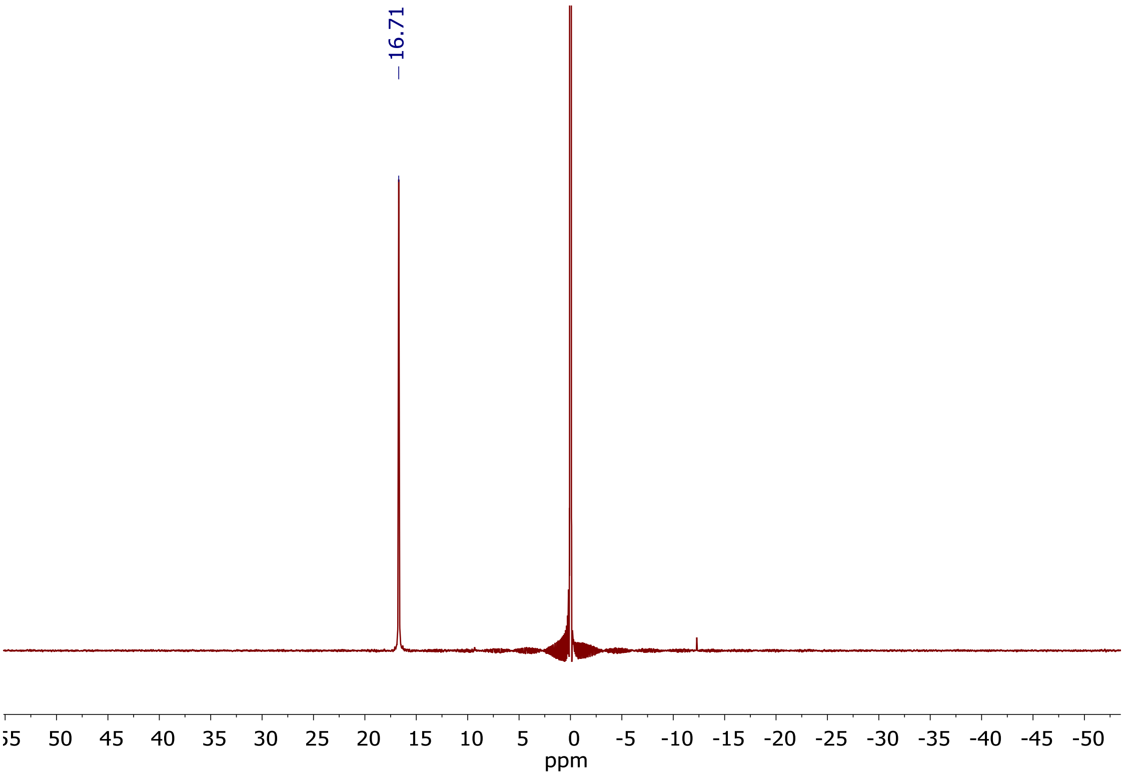

Figure S14: ^{31}P NMR (162 MHz, $CDCl_3$, 298 K, 85% H_3PO_4) of tetrabutylammonium benzyl phosphonate **S3**. Peak at -12.6 ppm, belongs to an impurity in the internal standard.

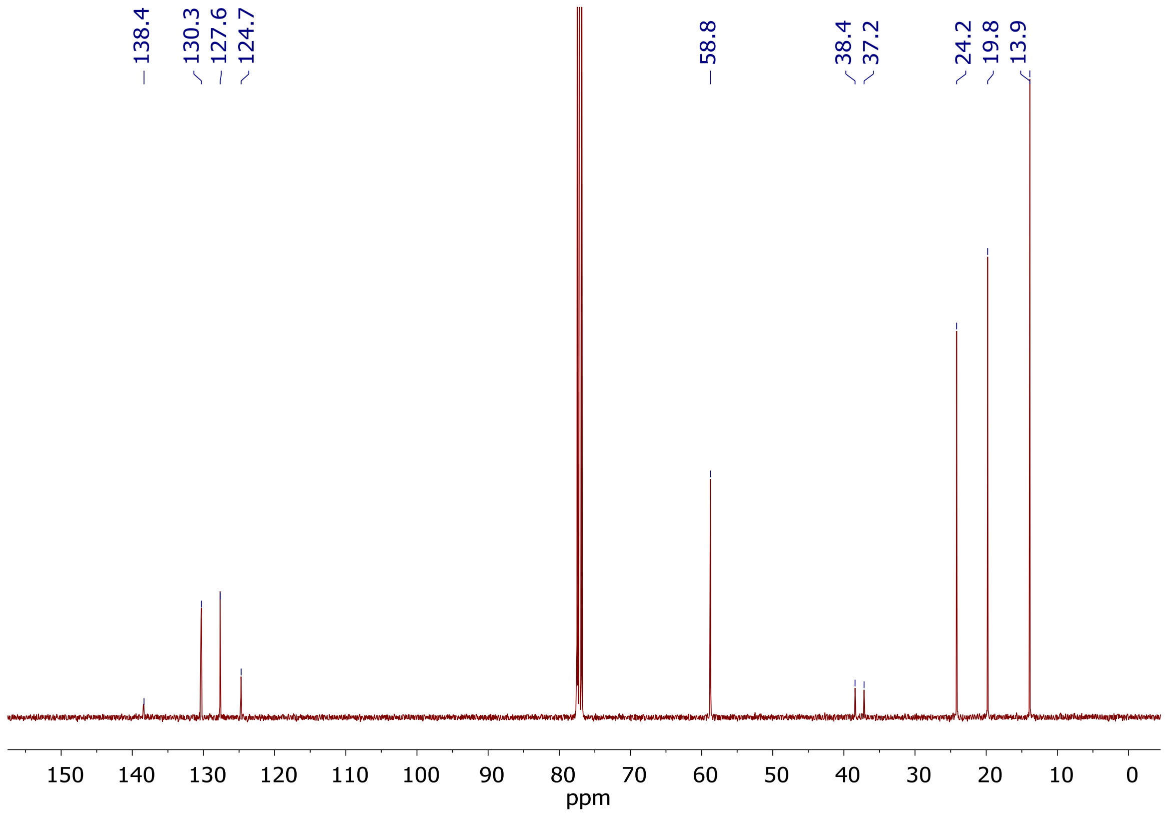

Figure S15: ^{13}C NMR (101 MHz, $CDCl_3$, 298 K) of tetrabutylammonium benzyl phosphonate **S3**.

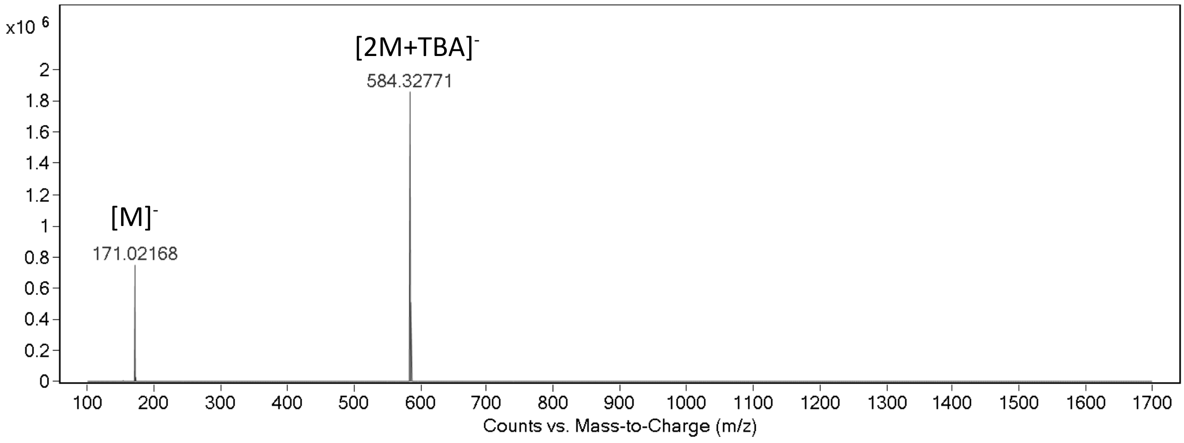

Figure S16: HRMS (ESI, negative mode) of tetrabutylammonium benzyl phosphonate **S3**.

Synthesis of *bis*-tetrabutylammonium benzyl pyrophosphonate (benzyl-PP) 2

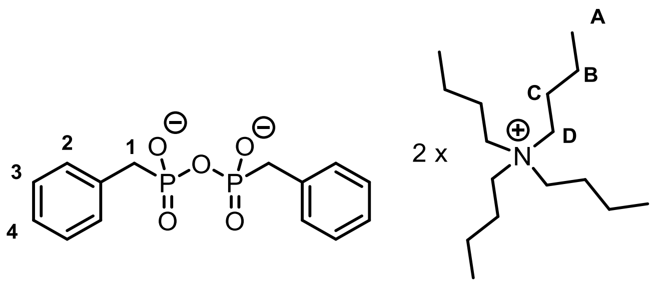

2

150 mg (0.35 mmol) benzyl phosphonate **S3** were dissolved in 15 mL DCM and 558 µL (3.50 mmol, 10 equiv.) *N,N'*-diisopropylcarbodiimide (DIC) were added. The mixture was stirred at room temperature for 48 h. DCM was evaporated and the residue was dissolved in 4 mL H_2O / MeCN (1:1) mixture and MPLC on an Interchim PF-15C18HP-F0025 C18 column with a mobile phase of MeCN and water (20-30% MeCN) and subsequent lyophilization yielded 40 mg (0.05 mmol, 29%) of the dimer as a colourless oil.

^{1}H-NMR (400 MHz, DMSO-d$_6$): δ [ppm] = 7.32-7.28 (m, 4 H, **3**), 7.11 (t, ^{3}J = 7.63 Hz, 4 H, **2**), 7.04-6.98 (m, 2 H, **4**), 3.22-3.12 (m, 16 H, **D**), 2.93-2.85 (m, 4 H, **1**), 1.63-1.52 (m, 16 H, **C**), 1.35-1.26 (m, 16 H, **B**), 0.93 (t, ^{3}J = 7.41 Hz, 24 H, **A**).

^{13}C-NMR (101 MHz, DMSO-d$_6$): δ [ppm] = 139.8 (t), 130.0 (t), 127.0, 123.8, 57.5, 37.6 (d), 23.1, 19.2, 13.5.

^{31}P-NMR (162 MHz, DMSO-d$_6$, external standard: 85% H_3PO_4): δ [ppm] = 6.84.

HRMS(ESI (-)): m/z = 325.04006 (calc. 325.04002, δm/m = 0.12 ppm) [M]$^-$, 566.31640 (calc. 566.31697, δm/m = 1.01 ppm) [2M+TBA]$^-$.

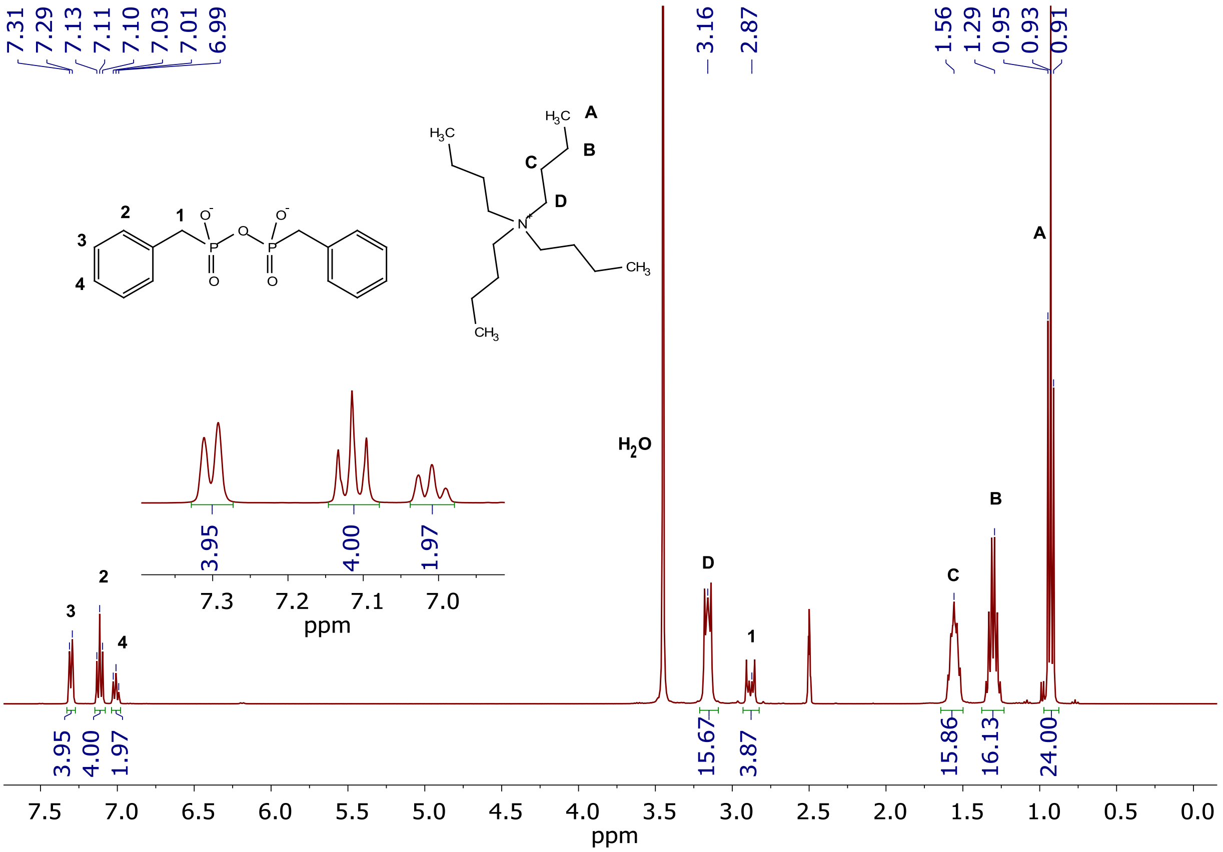

Figure S17: ^{1}H NMR (400 MHz, DMSO-d6, 298 K) of *bis*-tetrabutylammonium benzyl pyrophosphonate **2**.

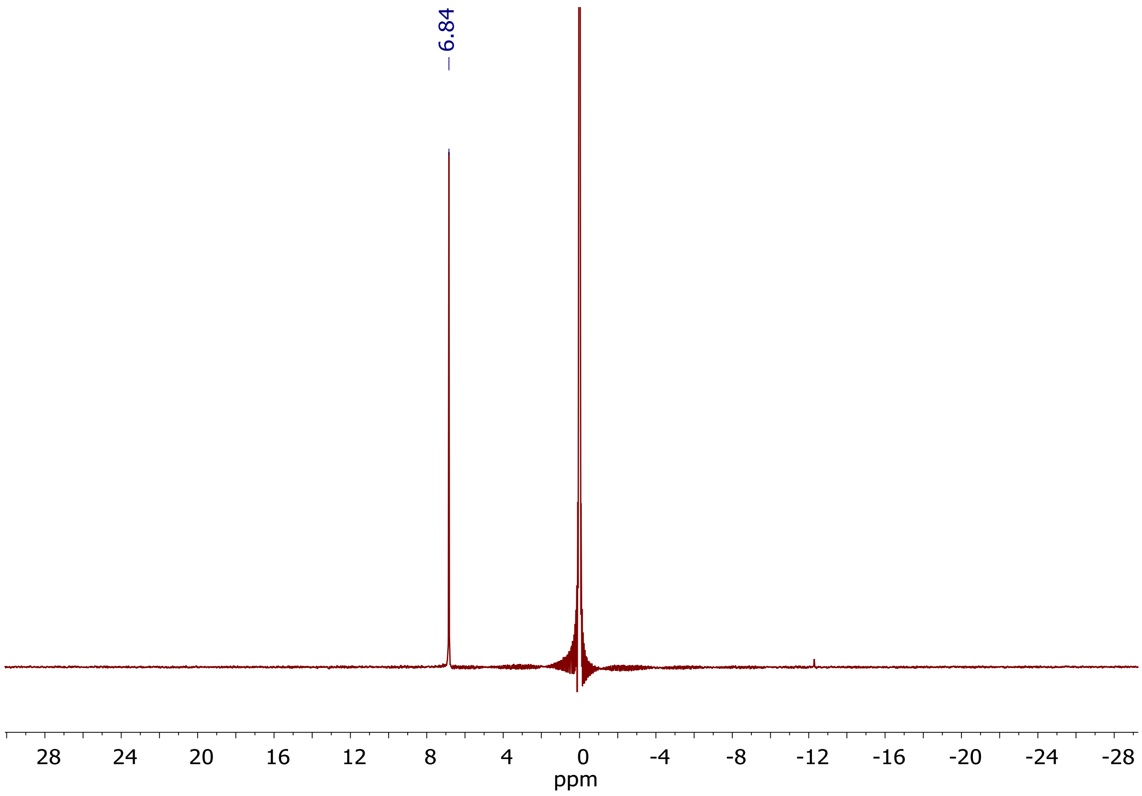

Figure S18: ^{31}P NMR (162 MHz, DMSO-d6, 298 K, 85% H_3PO_4) of *bis*-tetrabutylammonium benzyl pyrophosphonate **2**. Peak at -12.6 ppm, belongs to an impurity in the internal standard.

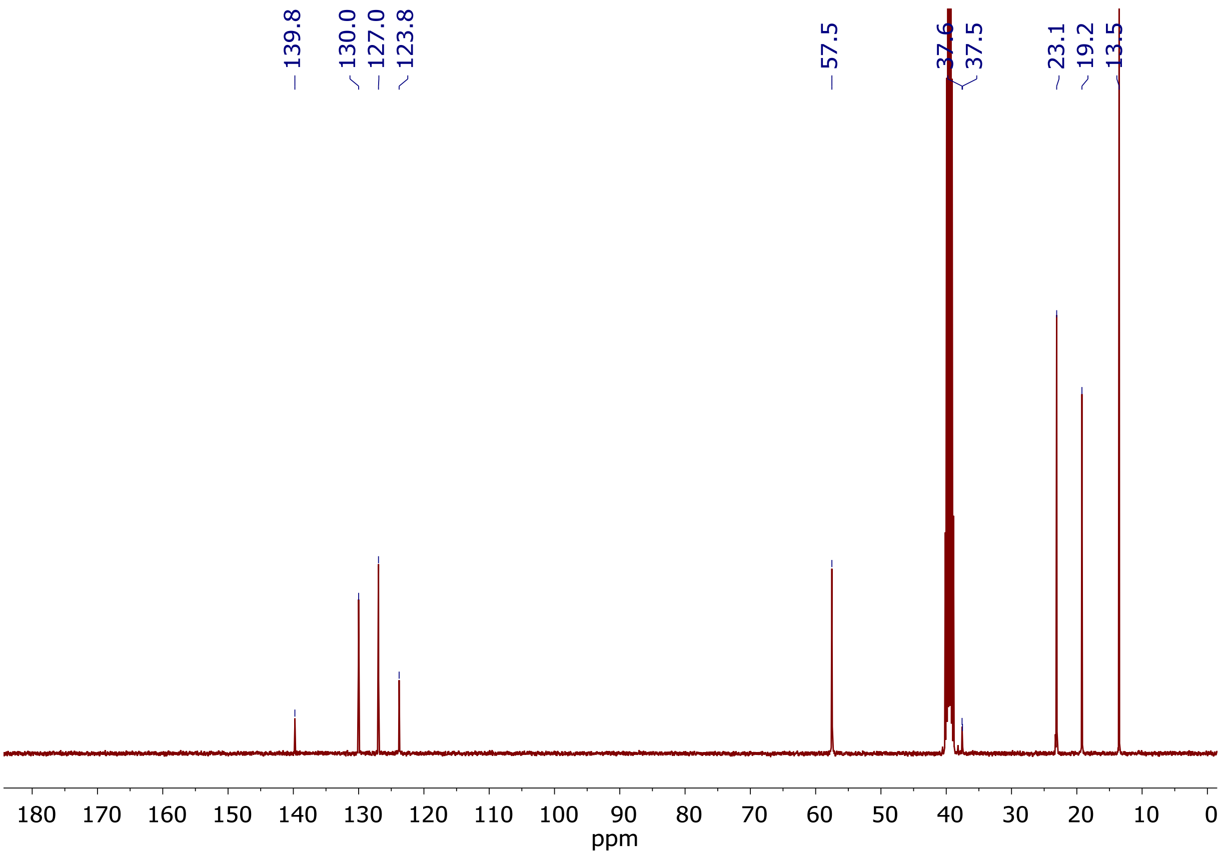

Figure S19: ^{13}C NMR (101 MHz, DMSO-d$_6$, 298 K) of *bis*-tetrabutylammonium benzyl pyrophosphonate **2**.

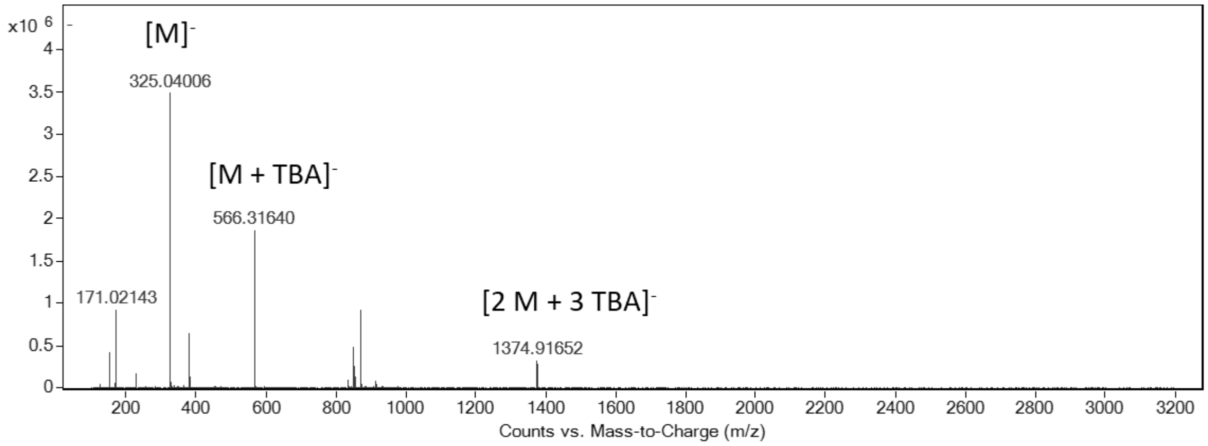

Figure S20: HRMS (ESI, negative mode) of *bis*-tetrabutylammonium benzyl pyrophosphonate **2**.

Synthesis of 2-fluorobenzylphosphonic acid S4

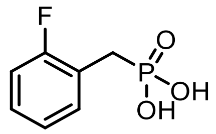

S4

450 mg (1.83 mmol) diethyl 2-fluorobenzylphosphonate were dissolved in 20 mL anhydrous DCM and 1.70 mL (12.8 mmol, 7 equiv.) trimethylsilylbromide were added. The mixture was stirred for 2 h at room temperature under inert atmosphere. DCM was evaporated and the residue was stirred for 1 h in anhydrous MeOH before the solvent was removed. MPLC on an Interchim PF-15C18HP-F0025 C18 column with a mobile phase of MeCN and 25 mM formic acid (5-35% MeCN) and subsequent lyophilization yielded 260 mg (1.36 mmol, 75%) of the desired product as a colourless solid.

^{1}H-NMR (400 MHz, DMSO-d$_6$): δ [ppm] = 7.35 (m, 1 H, **Ar**), 7.23 (m, 1 H, **Ar**), 7.12 (m, 2 H, **Ar**), 2.96 (d, $^{2}J_{H\text{-}P}$ = 21.2 Hz, 2 H, **CH$_2$**).

^{13}C-NMR (101 MHz, DMSO-d$_6$): δ [ppm] = 161.7 – 159.2 (dd), 132.1 (t), 128.1 (dd), 124.0 (t), 121.4 (dd), 115.0 (dd), 28.8 – 27.5 (dd).

^{31}P-NMR (162 MHz, DMSO-d$_6$, external standard: 85% H_3PO_4): δ [ppm] = 20.95.

^{19}F-NMR (367 MHz, **DMSO-d$_6$):** δ [ppm] = -117.3.

HRMS(ESI (-)): m/z = 189.01210 (calc. 189.01223, δm/m = 0.69 ppm) [M-H]$^-$

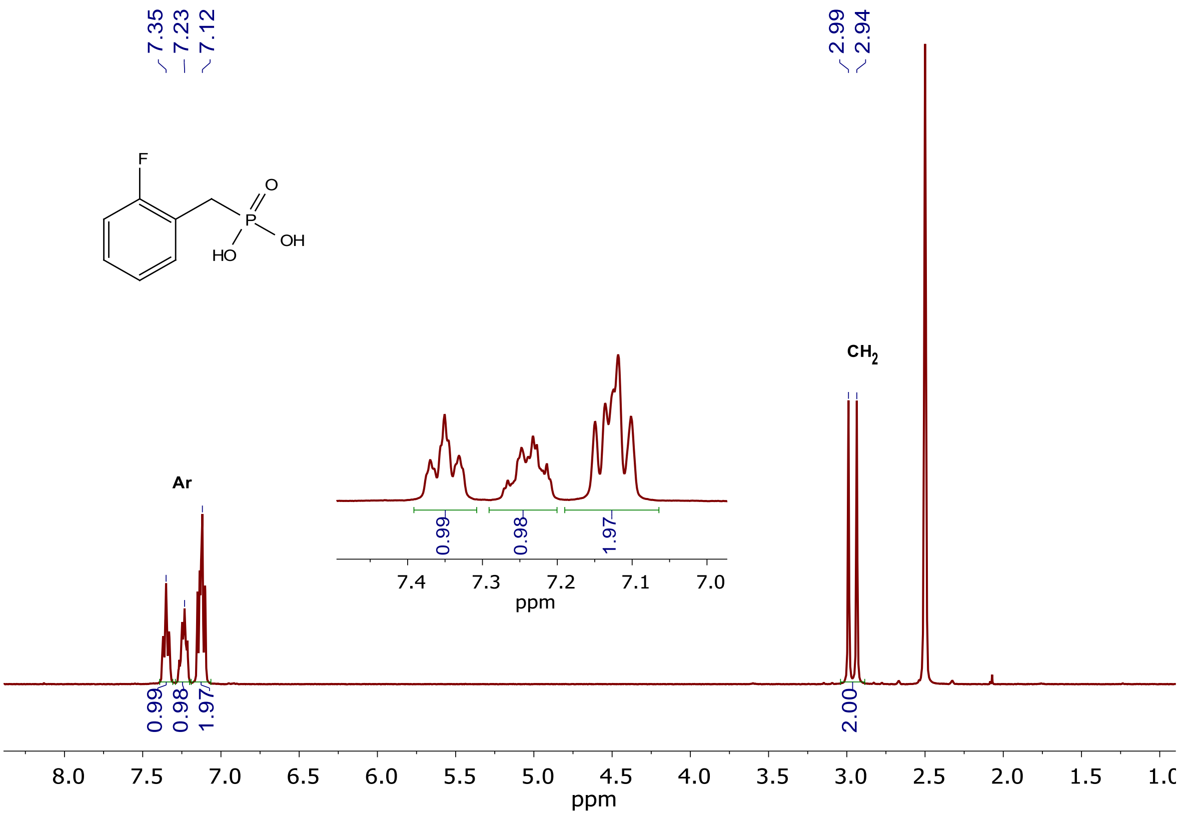

Figure S21: ^{1}H NMR (400 MHz, DMSO-d$_6$, 298 K) of 2-fluorobenzylphosphonic acid **S4**.

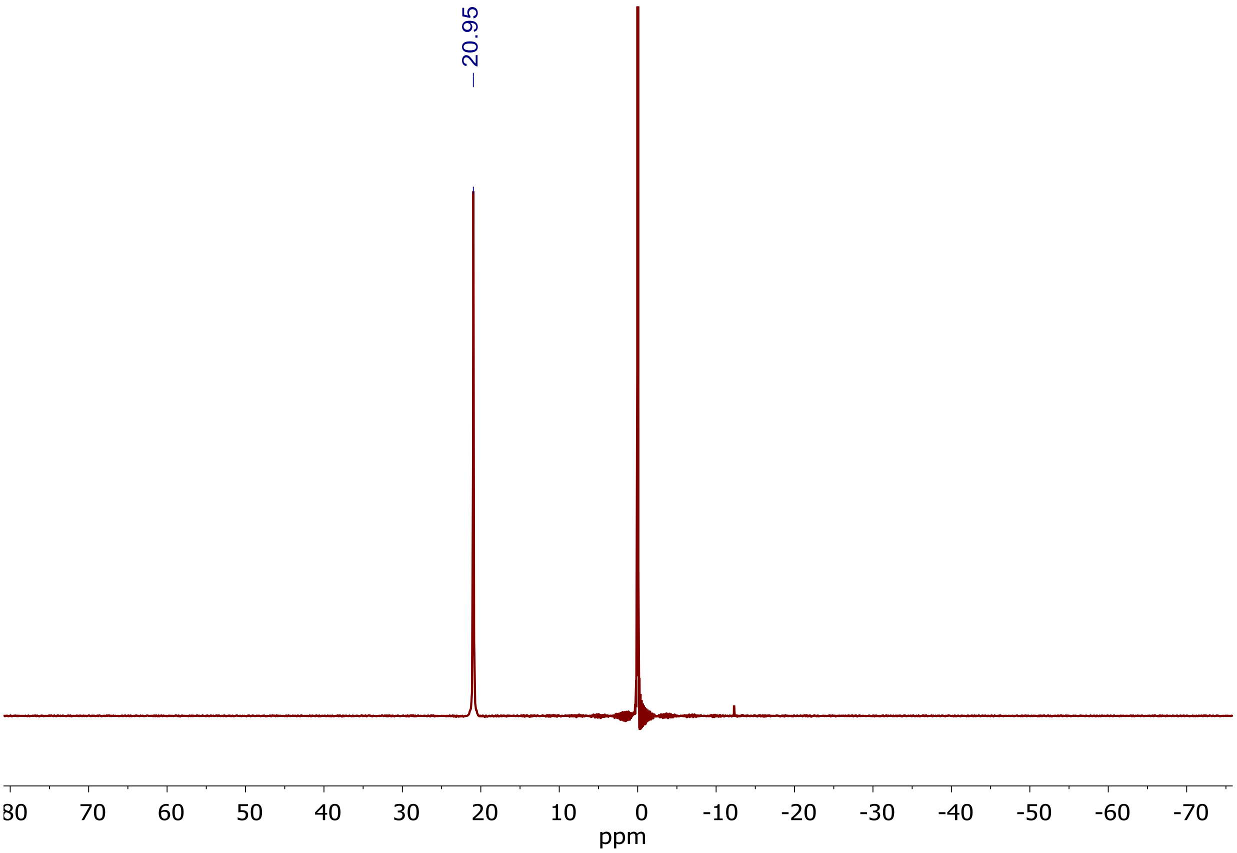

Figure S22: ^{31}P NMR (162 MHz, DMSO-d$_6$, 298 K, 85% H_3PO_4) of 2-fluorobenzylphosphonic acid **S4**. Peak at -12.6 ppm, belongs to an impurity in the internal standard.

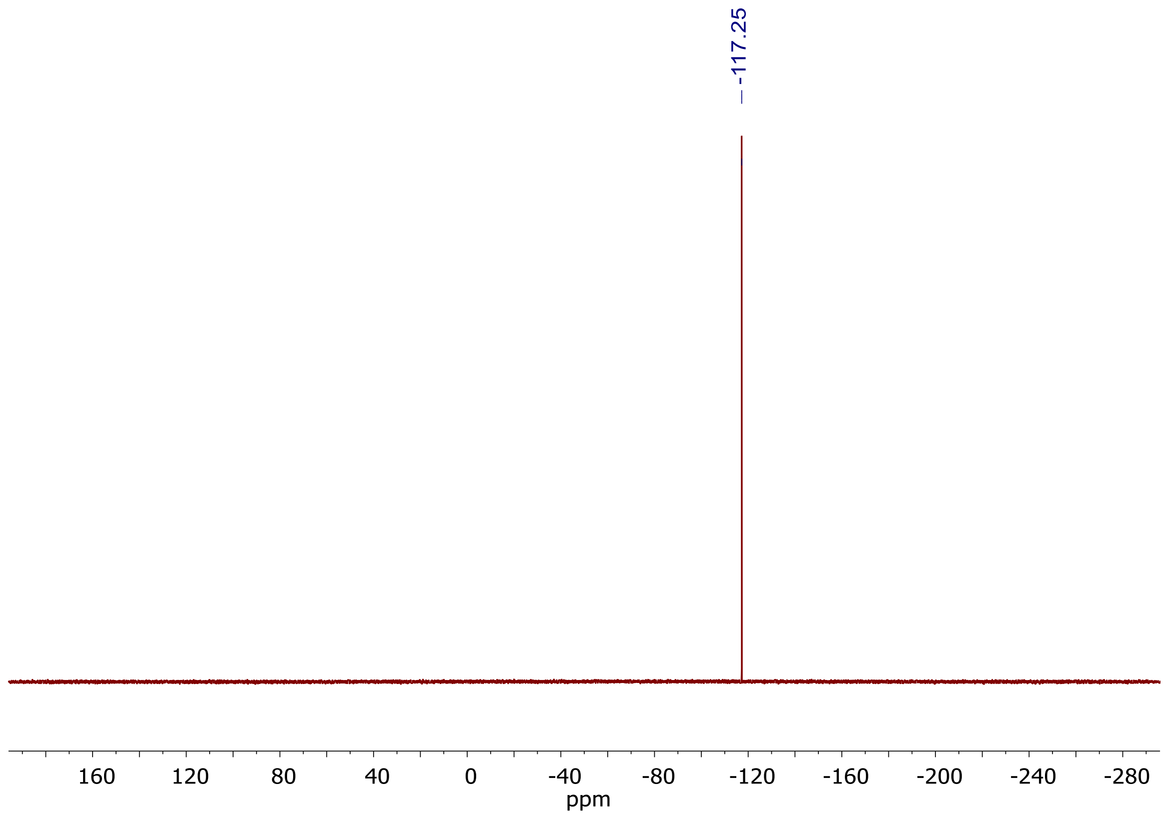

Figure S23: ^{19}F NMR (367 MHz, DMSO-d_6, 298 K) of 2-fluorobenzylphosphonic acid **S4**.

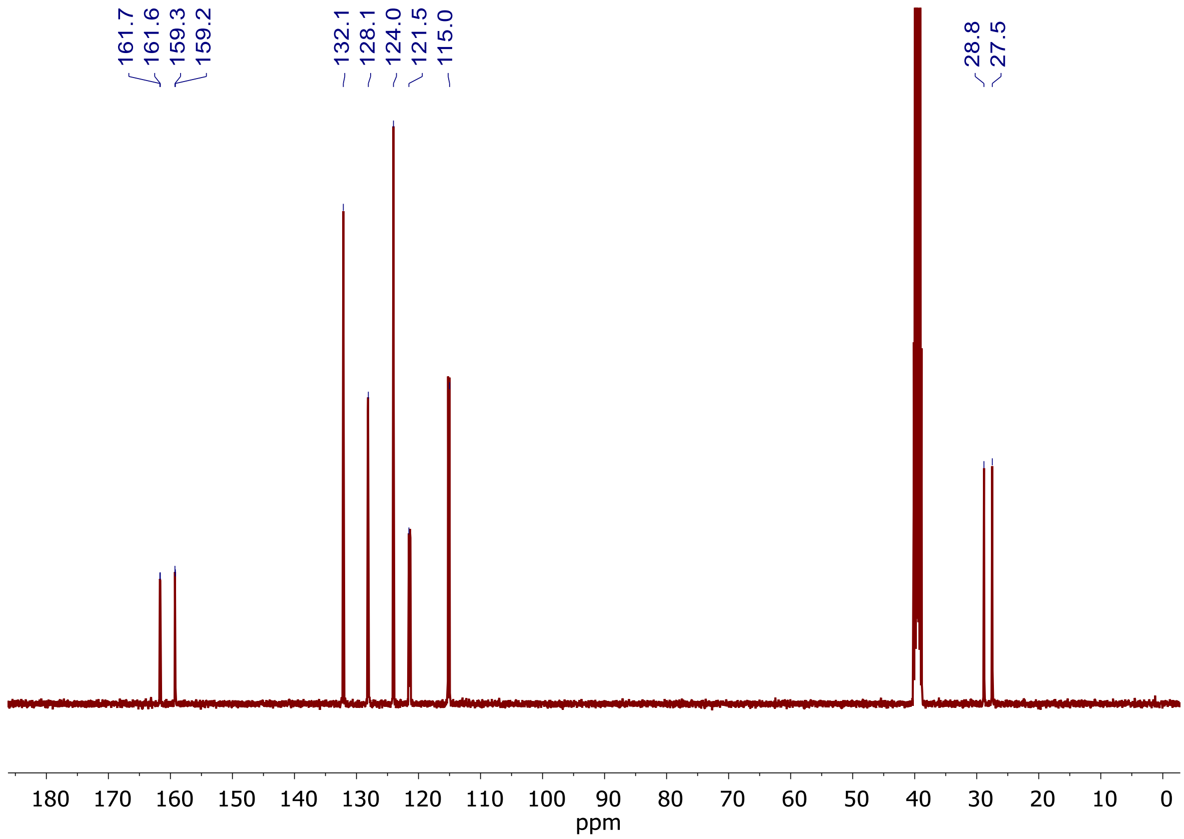

Figure S24: ^{13}C NMR (101 MHz, DMSO-d_6, 298 K) of 2-fluorobenzylphosphonic acid **S4**.

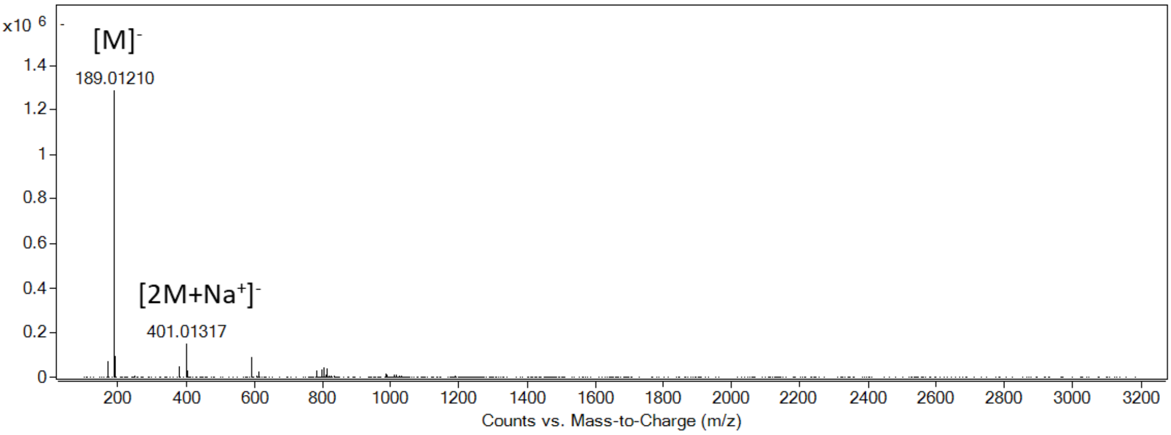

Figure S25: HRMS (ESI, negative mode) of 2-fluorobenzylphosphonic acid **S4**.

Synthesis of tetrabutylammonium 2-fluorobenzyl phosphonate S5

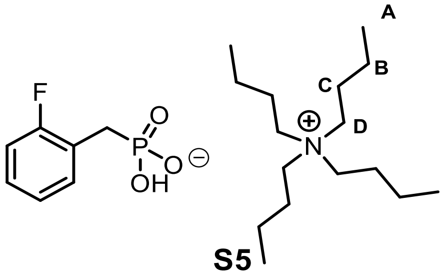

S5

120 mg (0.63 mmol) 2-fluorobenzylphosphonic acid **S4** and 505 mg (0.63 mmol, 1.0 equiv.) TBAOH · 30 H_2O were combined in a round bottom flask and dissolved in 8 mL MeOH. The mixture was stirring for 1 h at room temperature, before the solvent was removed and the product was dried in vacuo to afford 265 mg (0.62 mmol, quant.) of the desired TBA salt as a colourless solid.

^{1}H-NMR (400 MHz, $CDCl_3$): δ [ppm] = 7.70 (m, 1 H, **Ar**), 6.96 (m, 2 H, **Ar**), 6.86 (m, 1 H, **Ar**), 3.26 – 3.22 (m, 8 H, **D**), 2.93 (d, $^{2}J_{H\text{-}P}$ = 20.3 Hz, 2 H, **CH_2**), 1.56 (m, 8 H, **C**), 1.43 – 1.34 (m, 8 H, **B**), 0.95 (t, ^{3}J = 7.24 Hz, 12 H, **A**).

^{13}C-NMR (101 MHz, $CDCl_3$): δ [ppm] = 162.4 (d), 160.0 (d) 133.0 (t), 125.9 (d), 123.3 (t), 114.4 (d), 58.8, 29.8 (d), 24.2, 19.8, 13.9.

^{31}P-NMR (162 MHz, $CDCl_3$, external standard: 85% H_3PO_4): δ [ppm] = 15.98.

^{19}F-NMR (367 MHz, $CDCl_3$): δ [ppm] = -117.6.

HRMS(ESI (-)): m/z = 189.01262 (calc. 189.01223, δm/m = 2.06 ppm) $[M-H]^-$, 620.30897 (calc. 620.30869, δm/m = 0.45 ppm) $[2M+TBA]^-$.

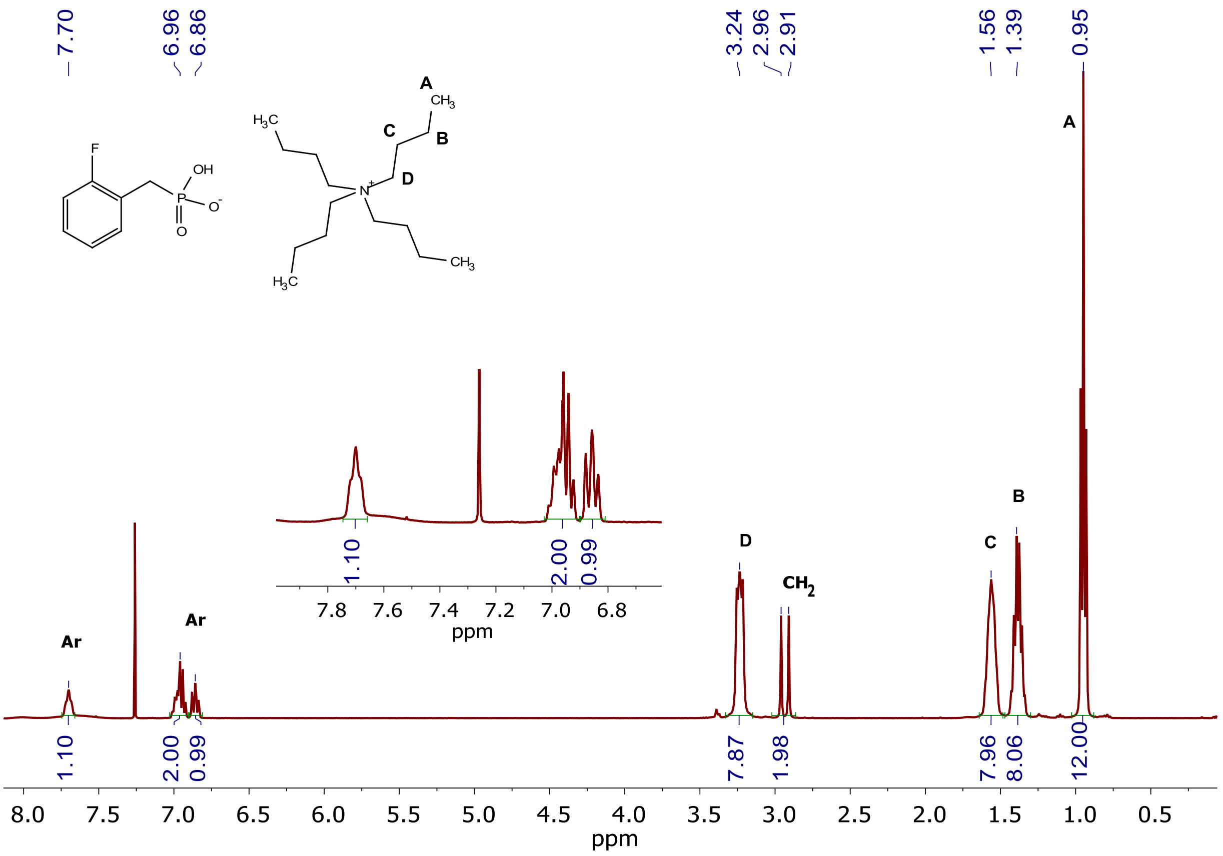

Figure S26: ^{1}H NMR (400 MHz, $CDCl_3$, 298 K) of tetrabutylammonium 2-fluorobenzyl phosphonate **S5**.

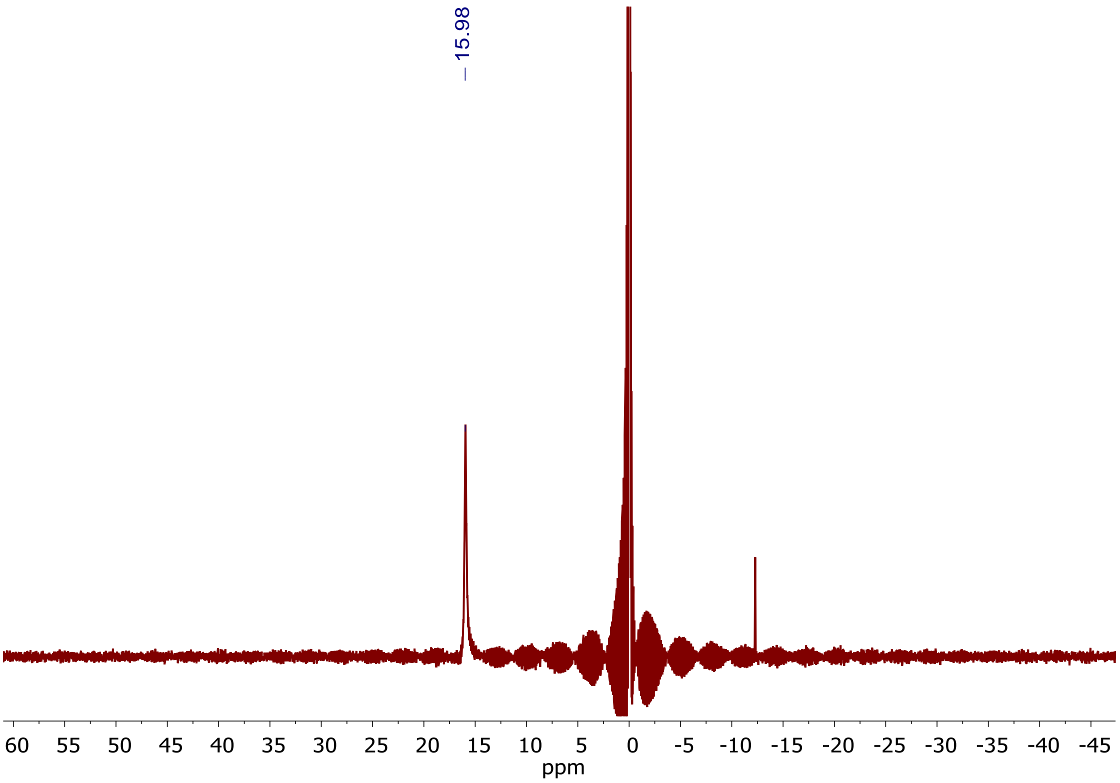

Figure S27: ^{31}P NMR (162 MHz, $CDCl_3$, 298 K, 85% H_3PO_4) of tetrabutylammonium 2-fluorobenzyl phosphonate **S5.** Peak at -12.6 ppm, belongs to an impurity in the internal standard.

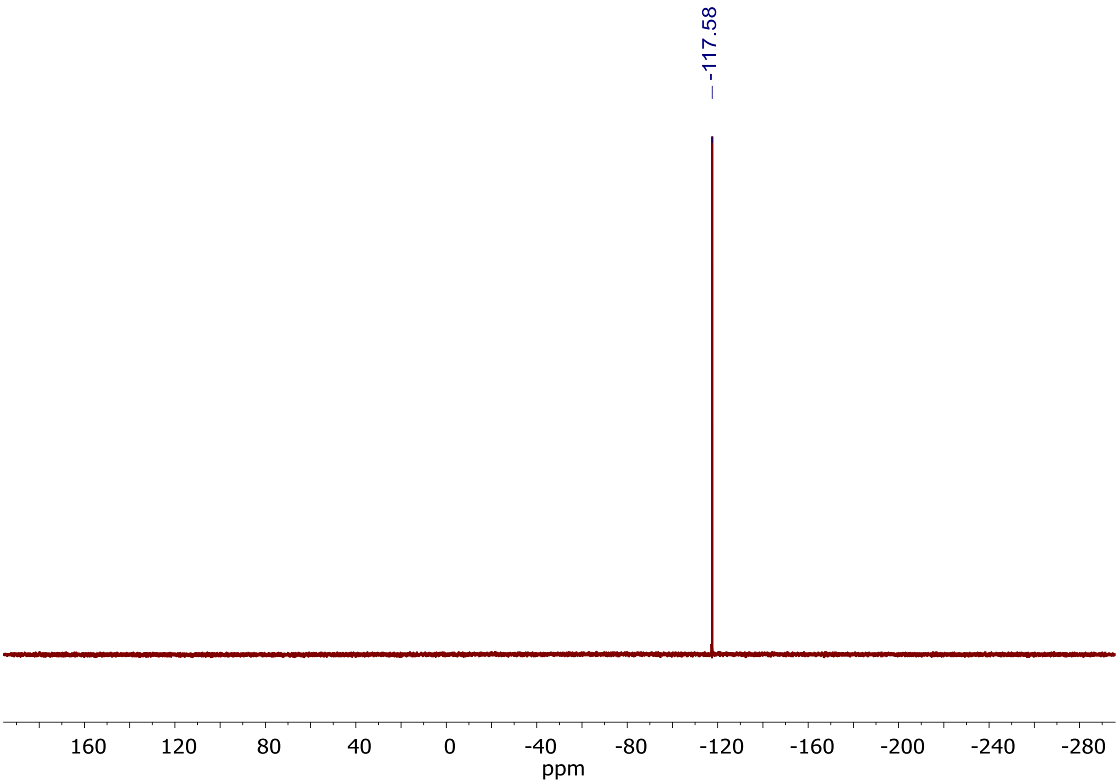

Figure S28: ^{19}F NMR (367 MHz, $CDCl_3$, 298 K) of tetrabutylammonium 2-fluorobenzyl phosphonate **S5**.

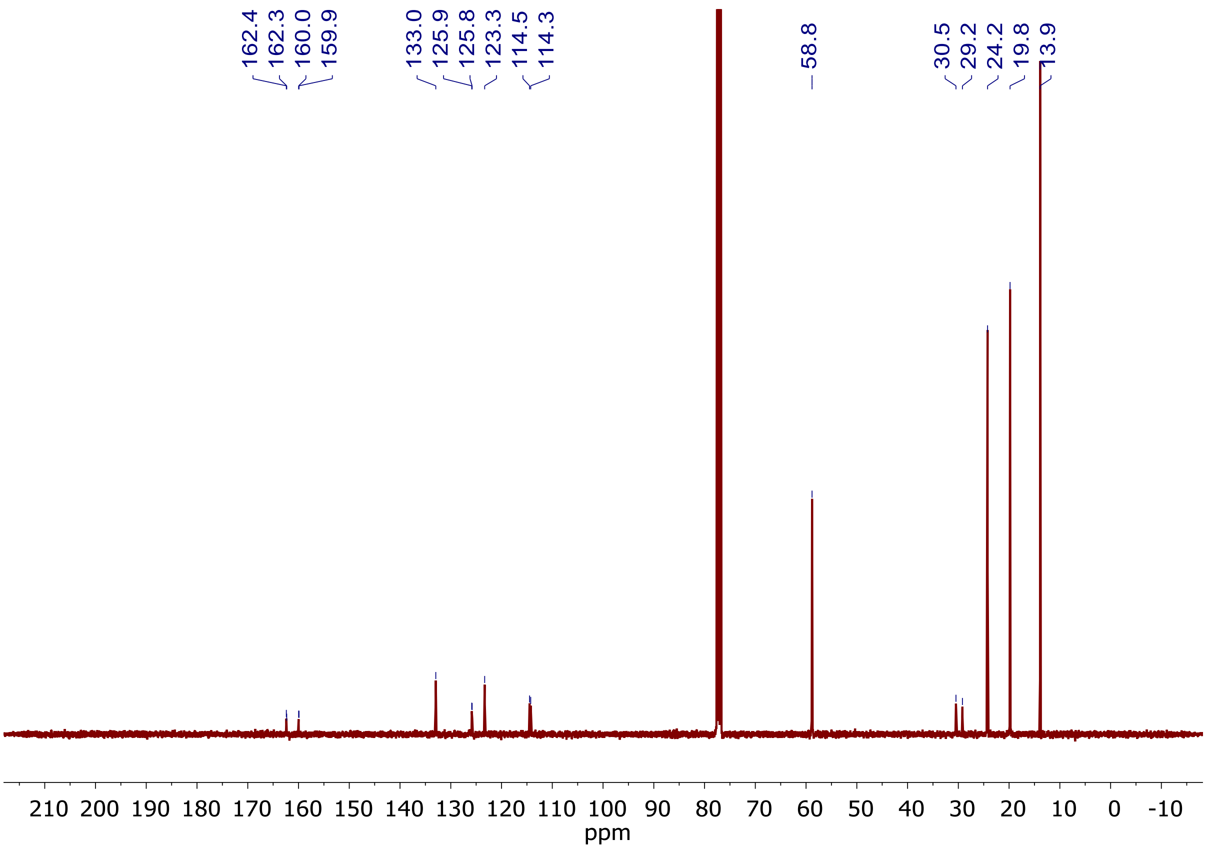

Figure S29: ^{13}C NMR (101 MHz, $CDCl_3$, 298 K) of tetrabutylammonium 2-fluorobenzyl phosphonate **S5**.

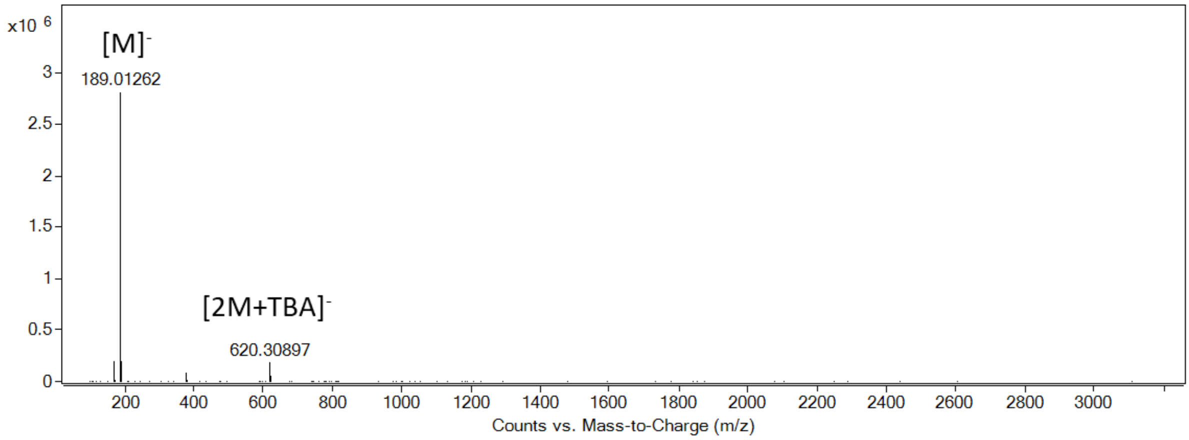

Figure S30: HRMS (ESI, negative mode) of tetrabutylammonium 2-fluorobenzyl phosphonate **S5**.

Synthesis of *bis*-tetrabutylammonium 2-fluorobenzyl pyrophosphonate (F-benzyl-PP) 3

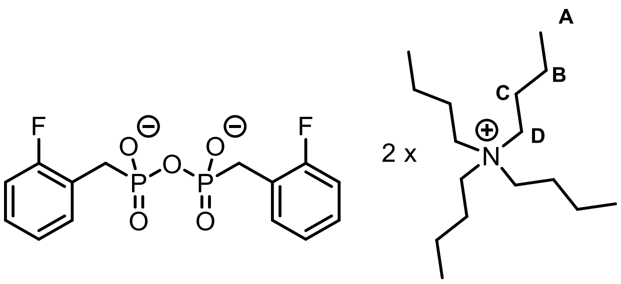

3

130 mg (0.30 mmol) tetrabutylammonium 2-fluorobenzyl phosphonate **S5** were dissolved in15 mL DCM and 560 µL (3.00 mmol, 10 equiv.) *N,N'*-diisopropylcarbodiimide (DIC) were added. The mixture was stirred at room temperature for 48 h. DCM was evaporated and the residue was dissolved in 4 mL of a H_2O / MeCN (3:1) mixture. MPLC on an Interchim PF-15C18HP-F0025 C18 column with a mobile phase of MeCN and water (20-30% MeCN) and subsequent lyophilization yielded 46 mg (0.054 mmol, 36%) of the dimer as a colourless oil.

^{1}H-NMR (400 MHz, DMSO-d_6): δ [ppm] = 7.74 (m, 2 H, **Ar**), 7.08-7.03 (m, 2 H, **Ar**), 6.97-6.93 (m, 4 H, **Ar**), 3.19-3.15 (m, 16 H, **D**), 2.97-2.92 (m, 4 H, **CH_2**), 1.60-1.52 (m, 16 H, **C**), 1.35-1.26 (m, 16 H, **B**), 0.93 (t, ^{3}J = 7.34 Hz, 24 H, **A**).

^{13}C-NMR (101 MHz, DMSO-d_6): δ [ppm] = 161.7 - 159.2 (dt), 132.6 (d), 126.7 (d), 125.5 (d), 122.9 (d), 113.9 (d), 57.5, 30.2 – 28.8 (dd), 23.1, 19.2, 13.5.

^{31}P-NMR (162 MHz, DMSO-d_6, external standard: 85% H_3PO_4): δ [ppm] = 5.56.

^{19}F-NMR (367 MHz, **$CDCl_3$):** δ [ppm] = -117.9

HRMS(ESI (-)): m/z = 361.02120 (calc. 361.02118, δm/m = 0.06 ppm) $[M]^-$, 602.29745 (calc. 602.29813, δm/m = 1.13 ppm) $[M+TBA]^-$

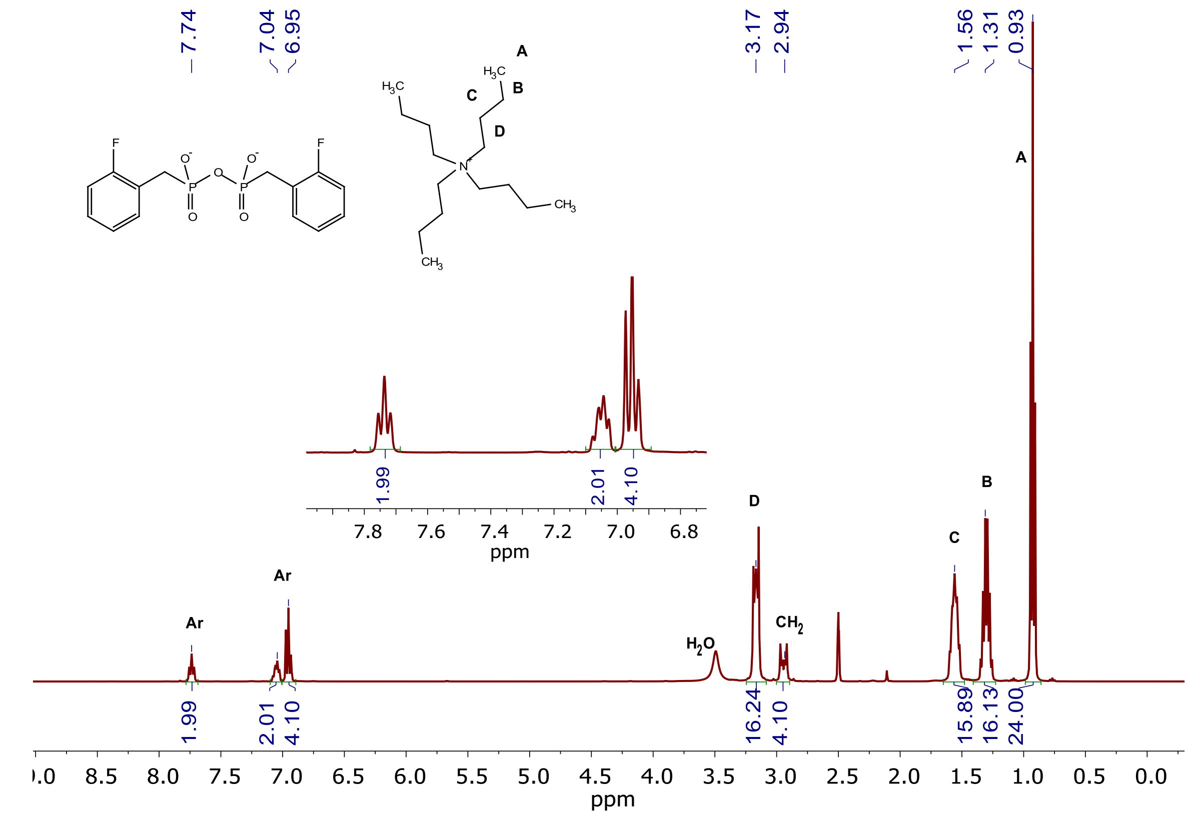

Figure S31: ^{1}H NMR (400 MHz, DMSO-d_6, 298 K) of *bis*-tetrabutylammonium 2-fluorobenzyl pyrophosphonate **3**.

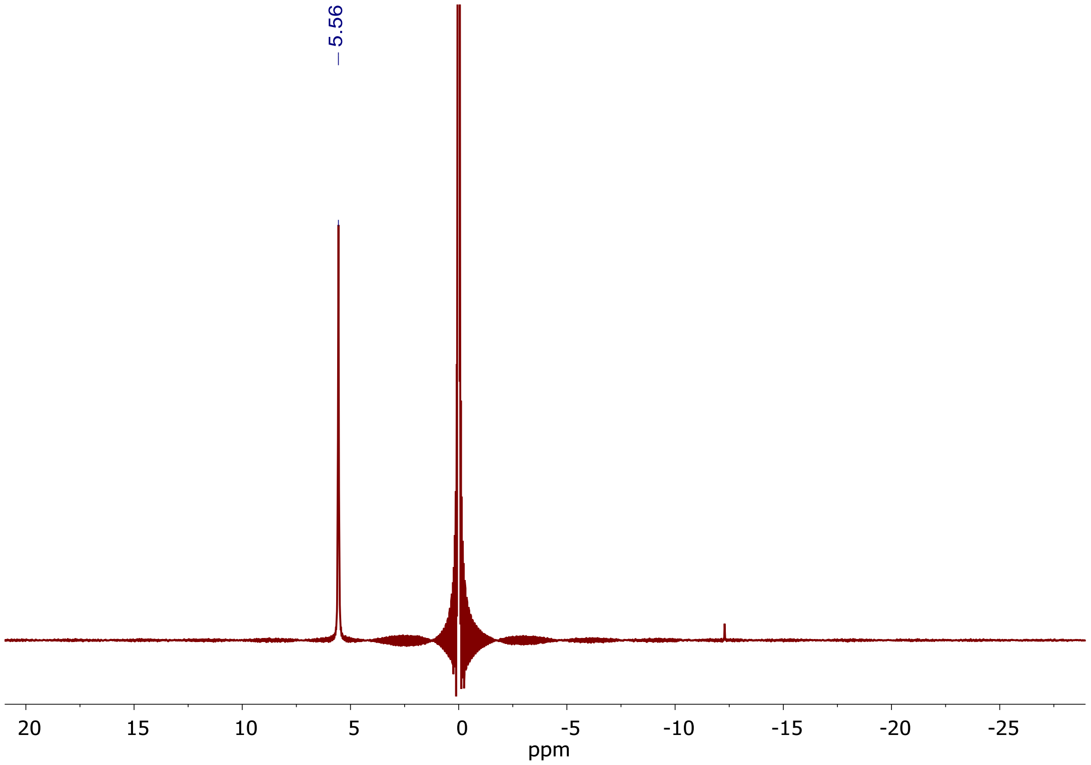

Figure S32: ^{31}P NMR (162 MHz, DMSO-d_6, 298 K, 85% H_3PO_4) of *bis*-tetrabutylammonium 2-fluorobenzyl pyrophosphonate **3**. Peak at -12.6 ppm, belongs to an impurity in the internal standard.

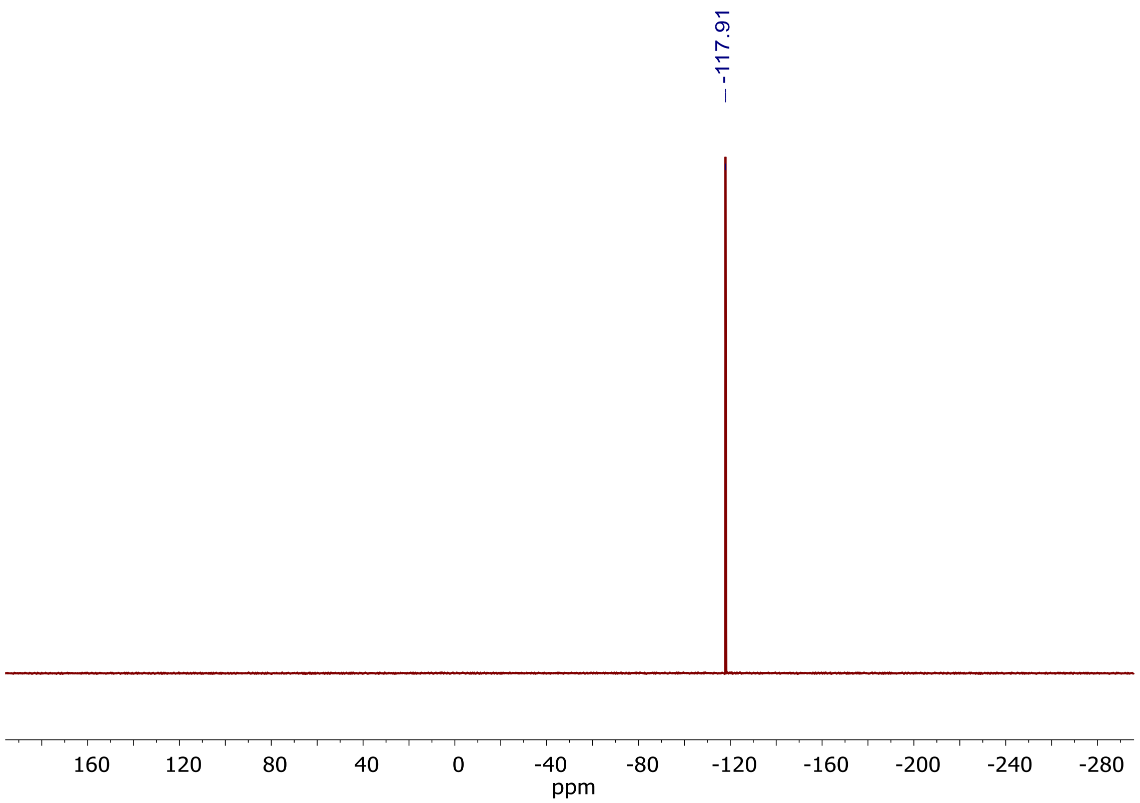

Figure S33: ^{19}F NMR (367 MHz, DMSO-d$_6$, 298 K) of *bis*-tetrabutylammonium 2-fluorobenzyl pyrophosphonate **3**.

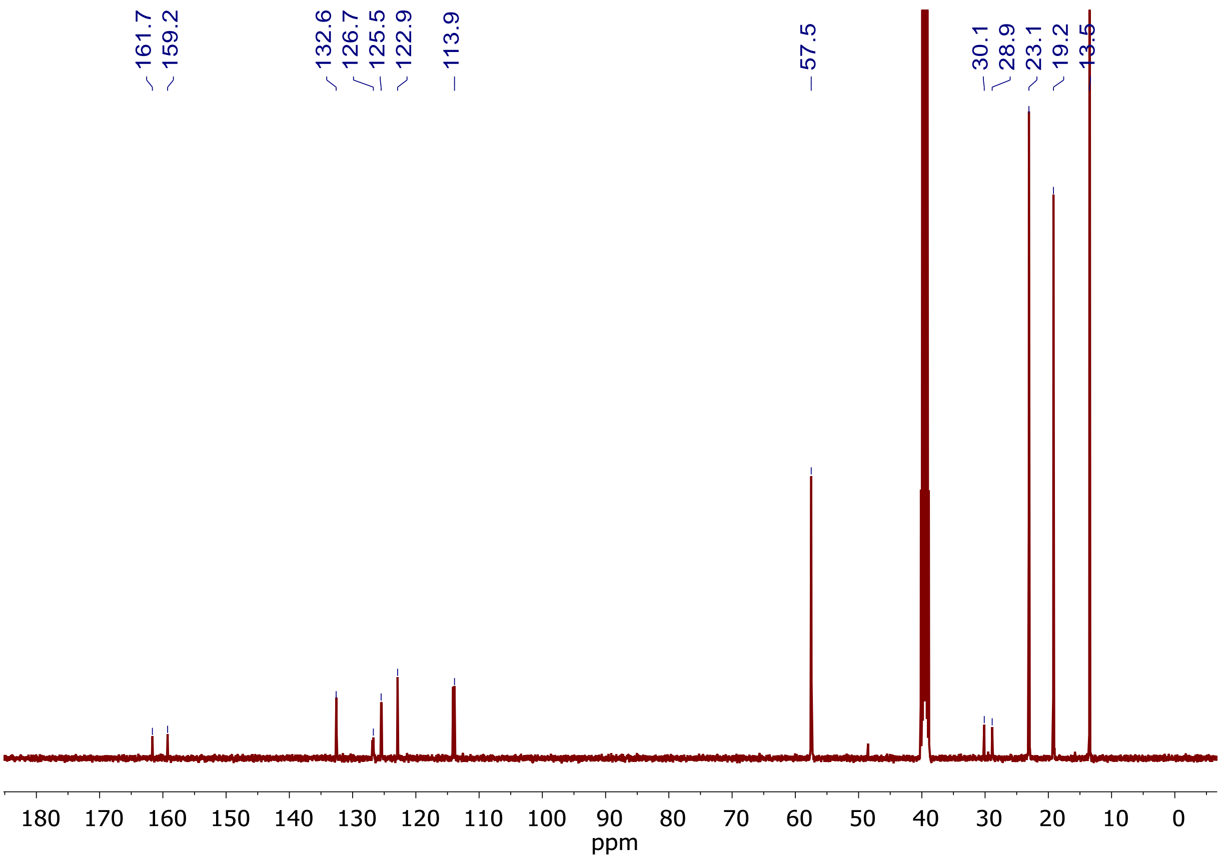

Figure S34: ^{13}C NMR (101 MHz, DMSO-d$_6$, 298 K) of *bis*-tetrabutylammonium 2-fluorobenzyl pyrophosphonate **3**.

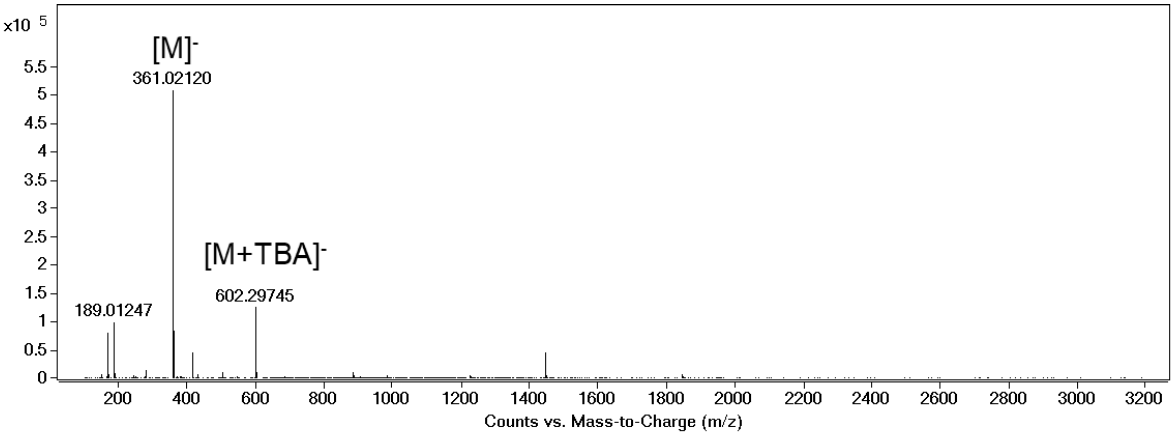

Figure S35: HRMS (ESI, negative mode) of *bis*-tetrabutylammonium 2-fluorobenzyl pyrophosphonate **3**.

Synthesis of 2-chlorobenzylphosphonic acid S6

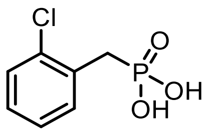

S6

431 µL (500 mg, 1.90 mmol) diethyl 2-chlorobenzylphosphonate were dissolved in 20 mL anhydrous DCM and 2.49 mL (19.0 mmol, 10 equiv.) trimethylsilylbromide were added. The mixture was stirred for 2 h at room temperature under inert atmosphere. DCM was evaporated and the residue was stirred for 1 h in anhydrous MeOH before the solvent was removed. MPLC on an Interchim PF-15C18HP-F0025 C18 column with a mobile phase of MeCN and 25 mM formic acid (5-35% MeCN) and subsequent lyophilization yielded 240 mg (1.16 mmol, 61%) of the desired product as a colourless solid.

^{1}H-NMR (400 MHz, DMSO-d$_6$): δ [ppm] = 7.42 (m, 2 H, **Ar**), 7.29 – 7.20 (m, 2 H, **Ar**), 3.14 (d, $^{2}J_{H\text{-}P}$ = 21.7 Hz, 2 H, **CH$_2$**).

^{13}C-NMR (101 MHz, DMSO-d$_6$): δ [ppm] = 133.4 – 132.3 (dd), 131.9 (d), 129.1 (d), 127.8 (d), 126.8 (d), 33.2 – 31.9 (d).

^{31}P NMR (162 MHz, DMSO-d$_6$, external standard: 85% H_3PO_4): δ [ppm] = 20.44.

HRMS(ESI (-)): m/z = 204.98322 (calc. 204.98268, δm/m = 3.12 ppm) [M-H]$^-$

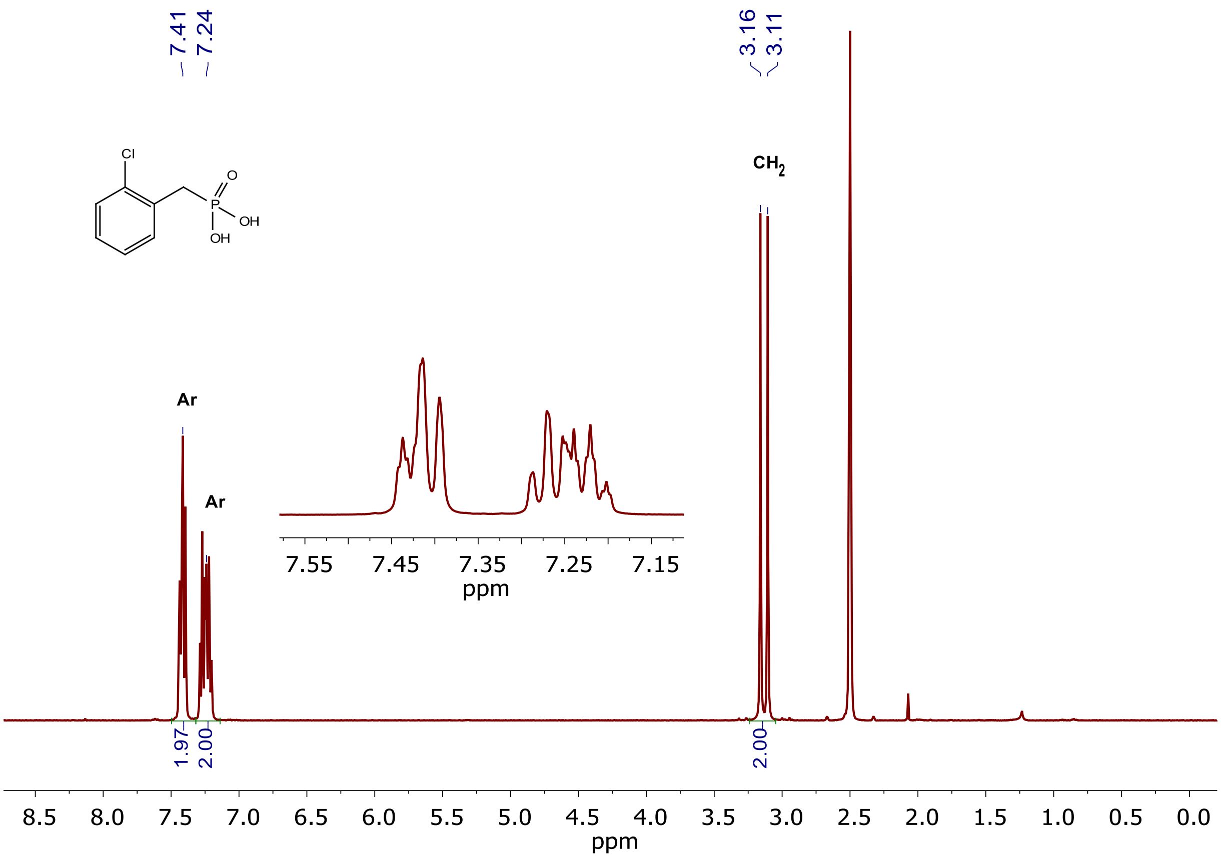

Figure S36: ^{1}H NMR (400 MHz, DMSO-d_6, 298 K) of 2-chlorobenzylphosphonic acid **S6**.

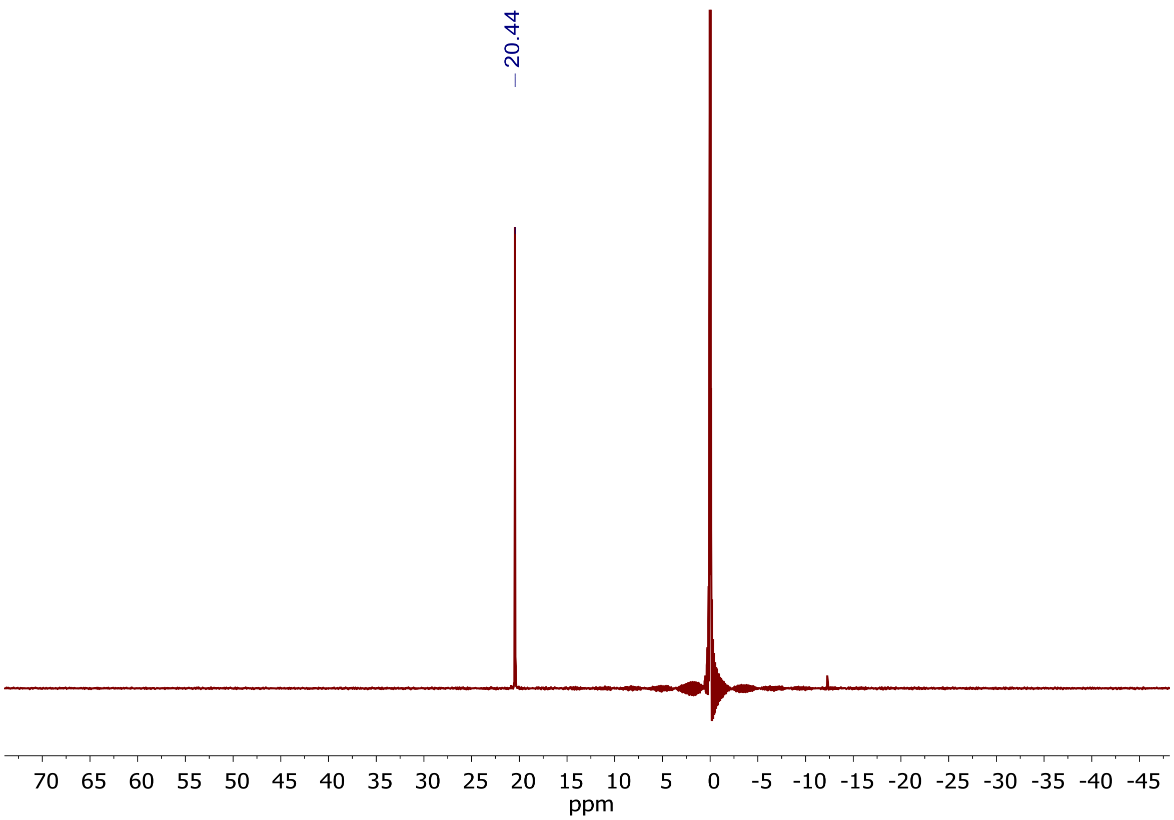

Figure S37: ^{31}P NMR (162 MHz, DMSO-d_6, 298 K, 85% H_3PO_4) of 2-chlorobenzylphosphonic acid **S6**. Peak at -12.6 ppm, belongs to an impurity in the internal standard.

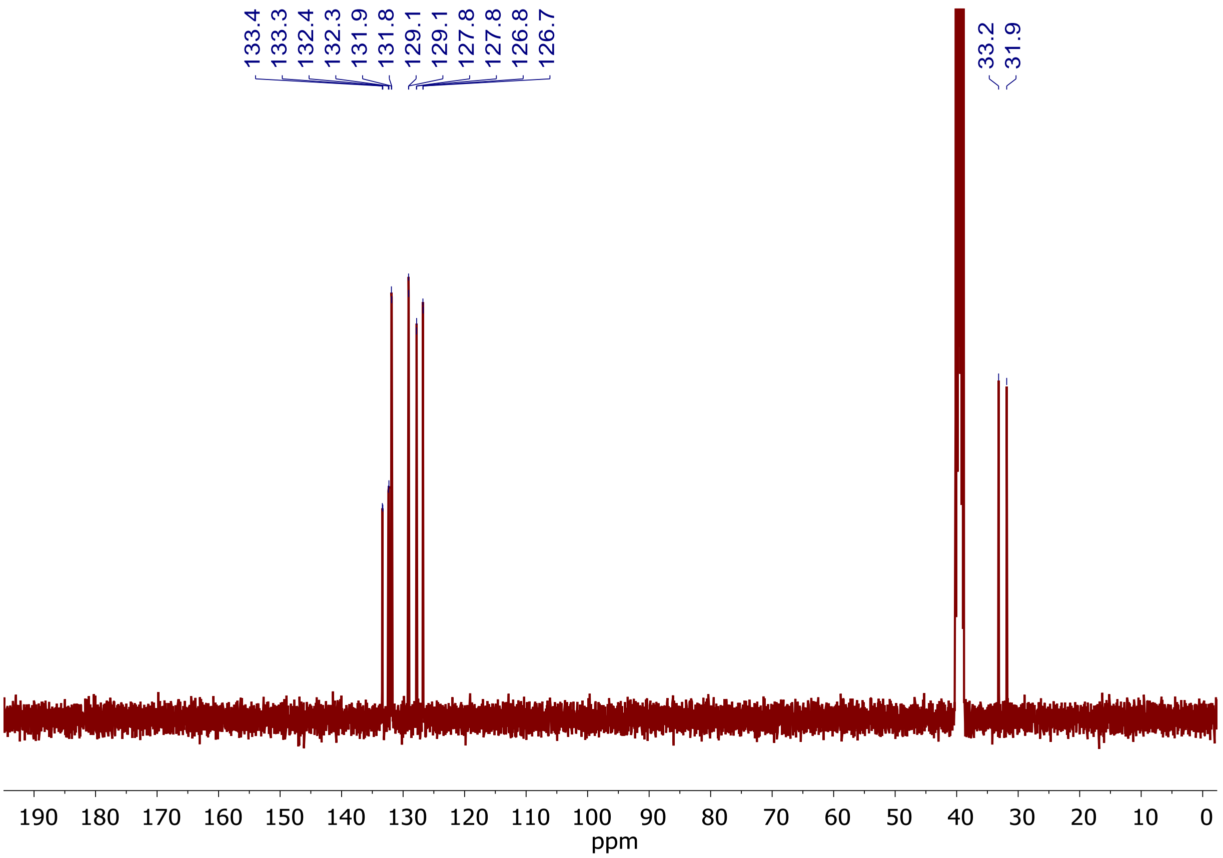

Figure S38: ^{13}C NMR (101 MHz, DMSO-d_6, 298 K) of 2-chlorobenzylphosphonic acid **S6**.

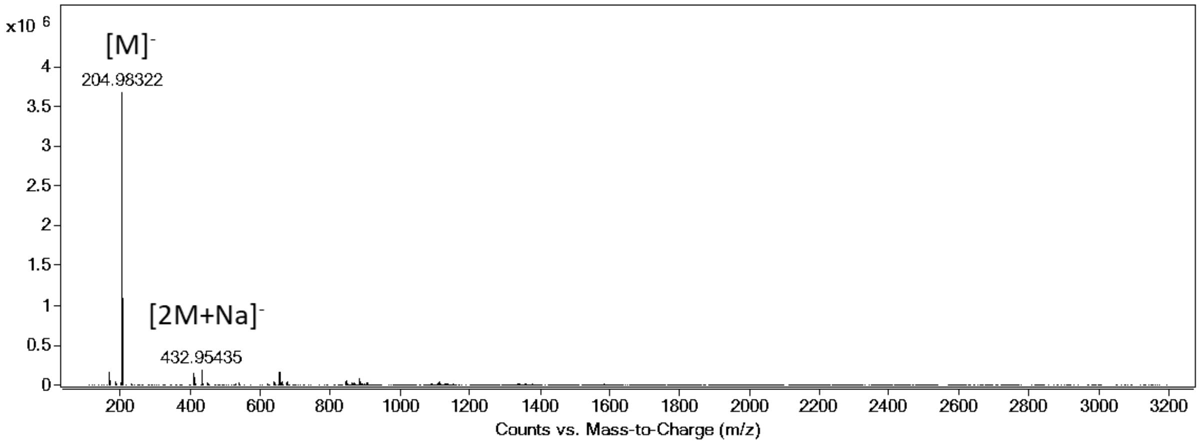

Figure S39: HRMS (ESI, negative mode) of 2-chlorobenzylphosphonic acid **S6**.

Synthesis of tetrabutylammonium 2-chlorobenzyl phosphonate S7

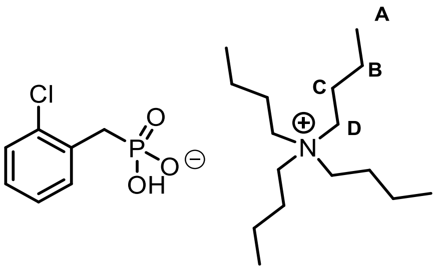

S7

225 mg (1.09 mmol) 2-chlorobenzylphosphonic acid **S6** and 872 mg (1.09 mmol, 1.0 equiv.) TBAOH · 30 H_2O were combined in a round bottom flask and dissolved in 15 mL MeOH. The mixture was stirring for 1 h at room temperature, before the solvent was removed and the product was dried in vacuo to afford 490 mg (1.09 mmol, quant.) of the desired TBA salt as a colourless solid.

^{1}H-NMR (400 MHz, $CDCl_3$): δ [ppm] = 7.82 (m, 1 H, **Ar**), 7.20 (m, 1 H, **Ar**), 7.08 (m, 1 H, **Ar**), 6.96 (m, 1 H, **Ar**), 3.23 – 3.19 (m, 8 H, **D**), 3.11 (d, $^{2}J_{H\text{-}P}$ = 20.5 Hz, 2 H, **CH_2**), 1.55 (m, 8 H, **C**), 1.42 – 1.33 (m, 8 H, **B**), 0.95 (t, ^{3}J = 7.31 Hz, 12 H, **A**).

^{13}C-NMR (101 MHz, $CDCl_3$): δ [ppm] = 137.2 (d), 134.4 (d) 132.6 (d), 128.6 (d), 126.1 (d), 125.9 (d), 58.8, 34.7 (d), 24.2, 19.8, 13.9.

^{31}P-NMR (162 MHz, $CDCl_3$, external standard: 85% H_3PO_4): δ [ppm] = 15.89.

HRMS(ESI (-)): m/z = 204.98344 (calc. 204.98213, δm/m = 6.39 ppm) $[M]^-$, 652.25043 (calc. 652.24904, δm/m = 2.13 ppm) $[2M+TBA]^-$.

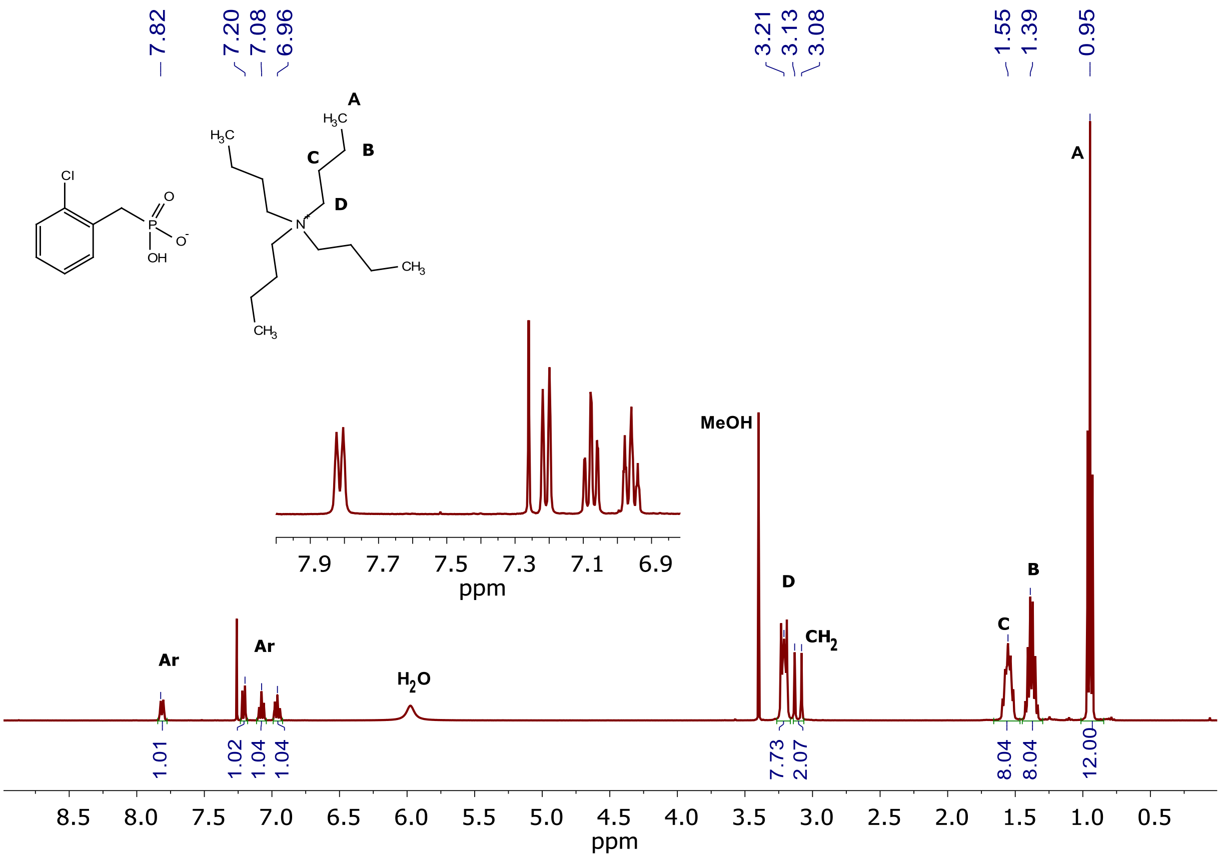

Figure S40: ^{1}H NMR (400 MHz, $CDCl_3$, 298 K) of tetrabutylammonium 2-chlorobenzyl phosphonate **S7**.

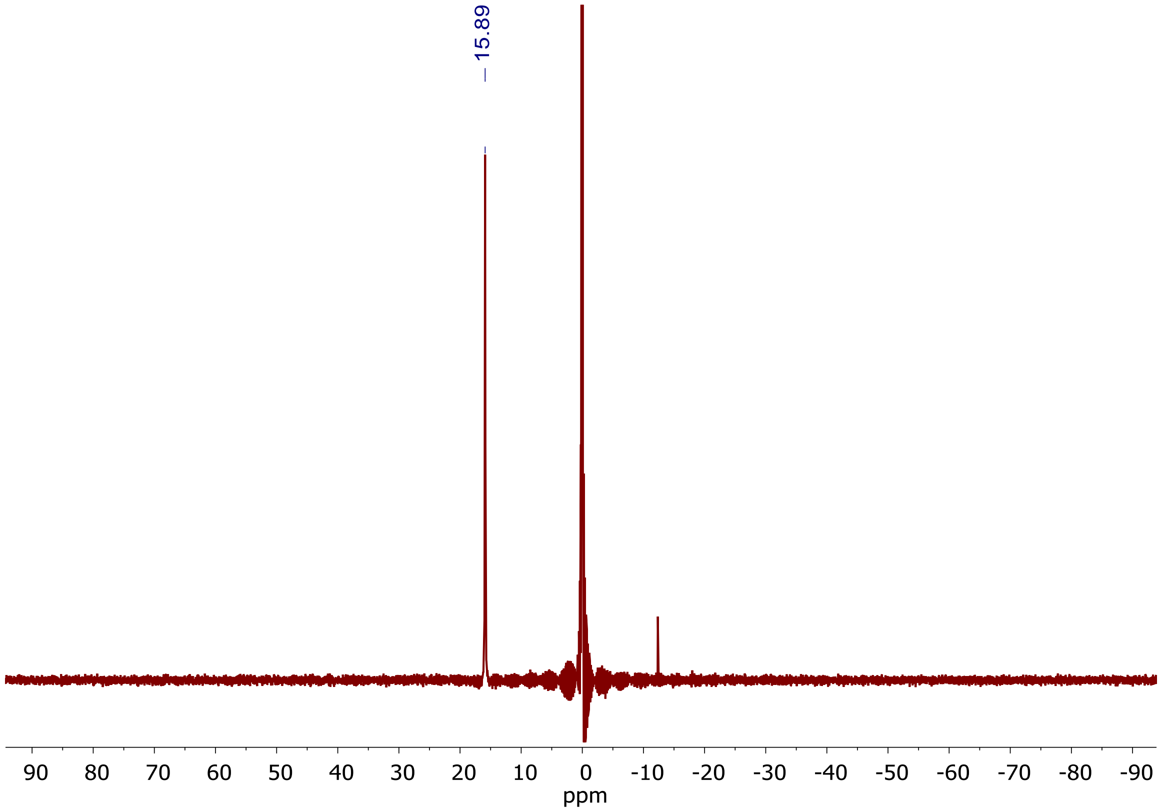

Figure S41: ^{31}P NMR (162 MHz, $CDCl_3$, 298 K, 85% H_3PO_4) of tetrabutylammonium 2-chlorobenzyl phosphonate **S7**. Peak at -12.6 ppm, belongs to an impurity in the internal standard.

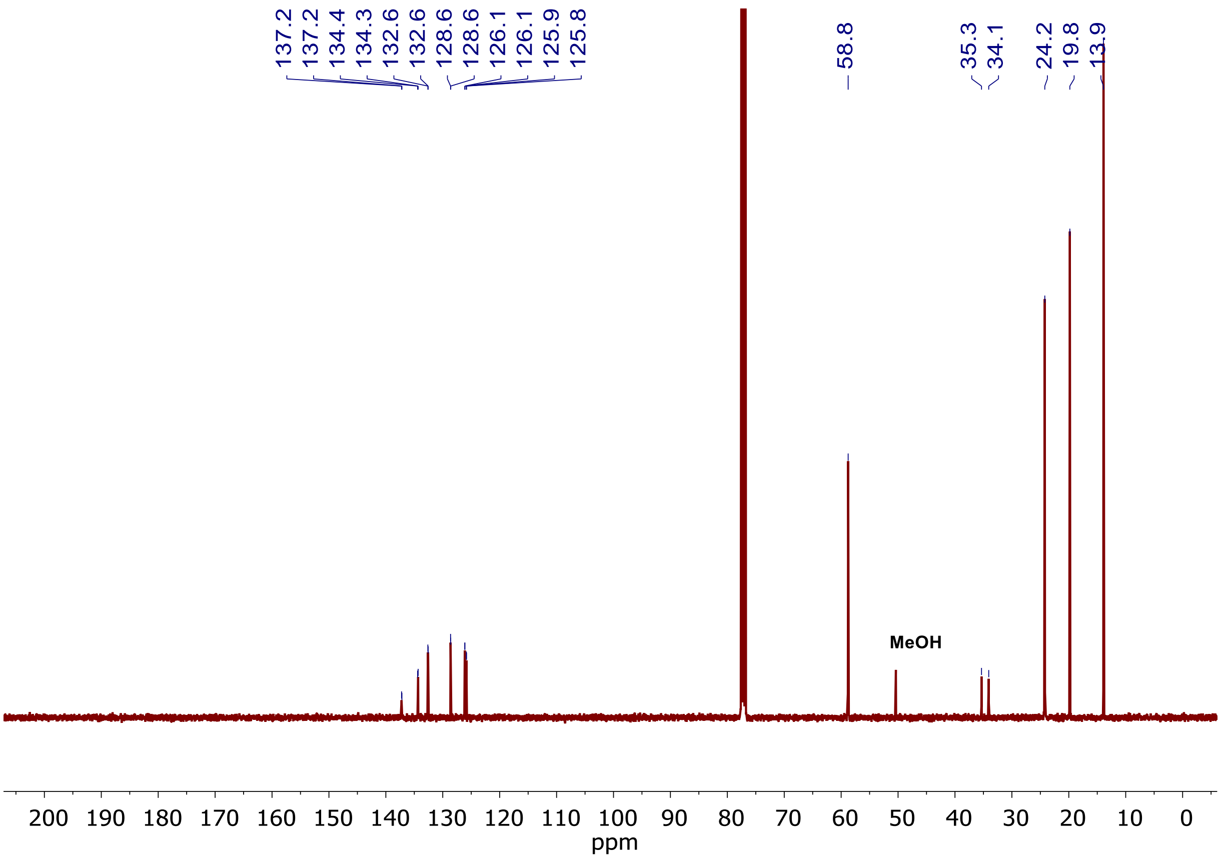

Figure S42: ^{13}C NMR (101 MHz, $CDCl_3$, 298 K) of tetrabutylammonium 2-chlorobenzyl phosphonate **S7**.

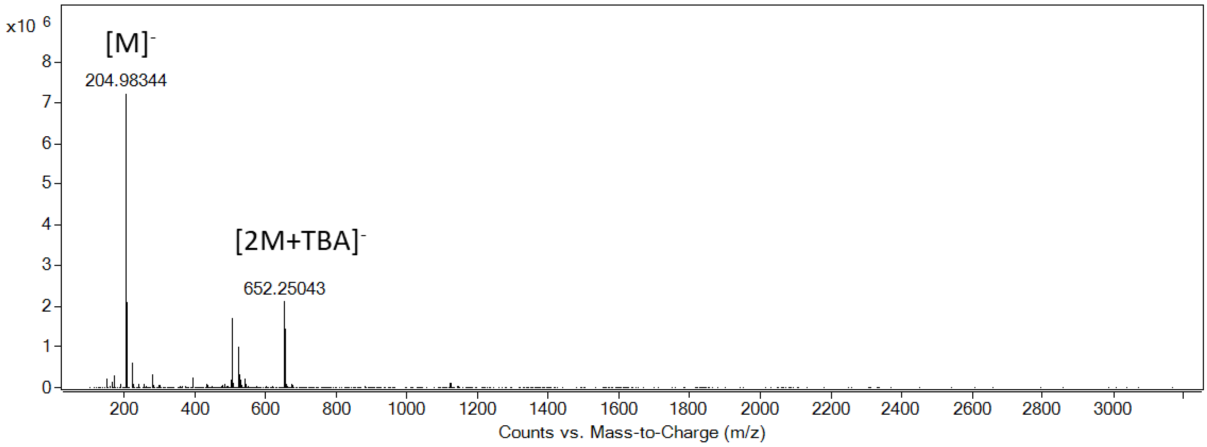

Figure S43: HRMS (ESI, negative mode) of tetrabutylammonium 2-chlorobenzyl phosphonate **S7**.

Synthesis of *bis*-tetrabutylammonium 2-chlorobenzyl pyrophosphonate (Cl-benzyl-PP) 4

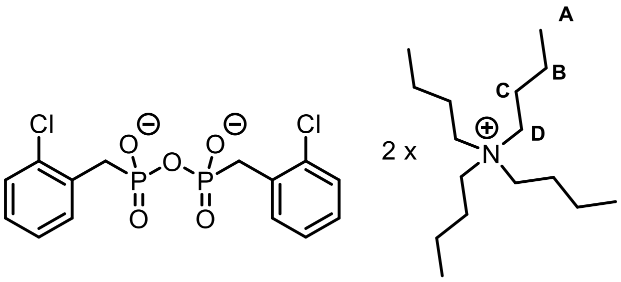

4

490 mg (1.09 mmol) tetrabutylammonium 2-chlorobenzyl phosphonate **S7** were dissolved in 30 mL DCM and 1.71 mL (10.9 mmol, 10 equiv.) *N,N'*-diisopropylcarbodiimide (DIC) were added. The mixture was stirred at room temperature for 48 h. DCM was evaporated and the residue was dissolved in 4 mL of a H_2O / MeCN (3:1) mixture. MPLC on an Interchim PF-15C18HP-F0025 C18 column with a mobile phase of MeCN and water (20-30% MeCN) and subsequent lyophilization yielded 160 mg (0.18 mmol, 33%) of the dimer as a colourless oil.

^{1}H-NMR (400 MHz, $CDCl_3$): δ [ppm] = 7.83 (m, 2 H, **Ar**), 7.19 (m, 2 H, **Ar**), 7.06 (m, 2 H, **Ar**), 6.96 (m, 2 H, **Ar**), 3.28 – 3.19 (m, 16 H, **D**), 3.30 – 3.26 (m, 4 H, **CH_2**), 1.59-1.51 (m, 16 H, **C**), 1.41-1.32 (m, 16 H, **B**), 0.92 (t, ^3J = 7.35 Hz, 24 H, **A**).

^{13}C-NMR (101 MHz, $CDCl_3$): δ [ppm] = 136.6, 134.4 (t), 132.6, 128.6, 126.2 (d), 126.0 (d), 58.7, 35.3 – 33.9 (dd), 24.2, 19.8, 13.8.

^{31}P-NMR (162 MHz, $CDCl_3$, external standard: 85% H_3PO_4): δ [ppm] = 8.52.

HRMS(ESI (-)): m/z = 392.96248 (calc. 392.96208, δm/m = 1.02 ppm) $[M]^-$, 634.23973 (calc. 634.23848, δm/m = 1.97 ppm) $[M+TBA]^-$

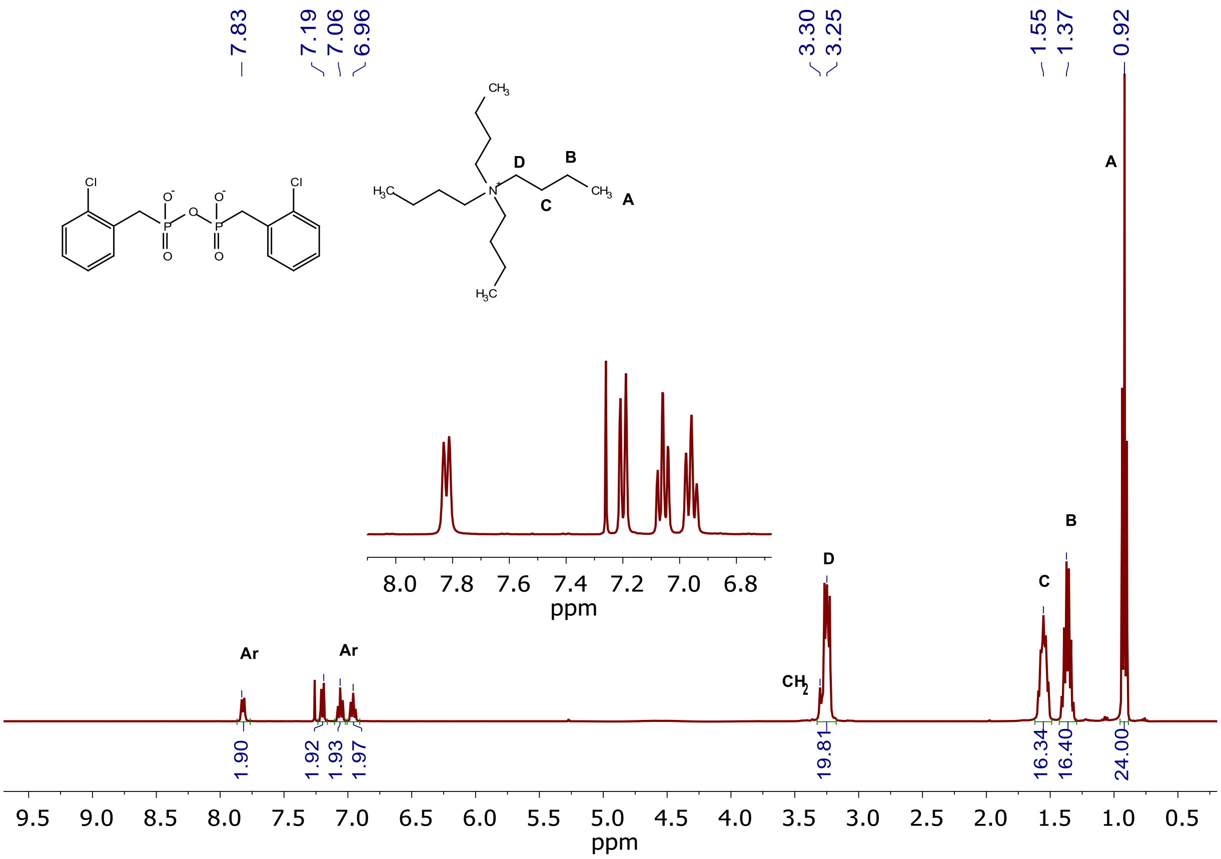

Figure S44: ^{1}H NMR (400 MHz, $CDCl_3$, 298 K) of *bis*-tetrabutylammonium 2-chlorobenzyl pyrophosphonate **4**.

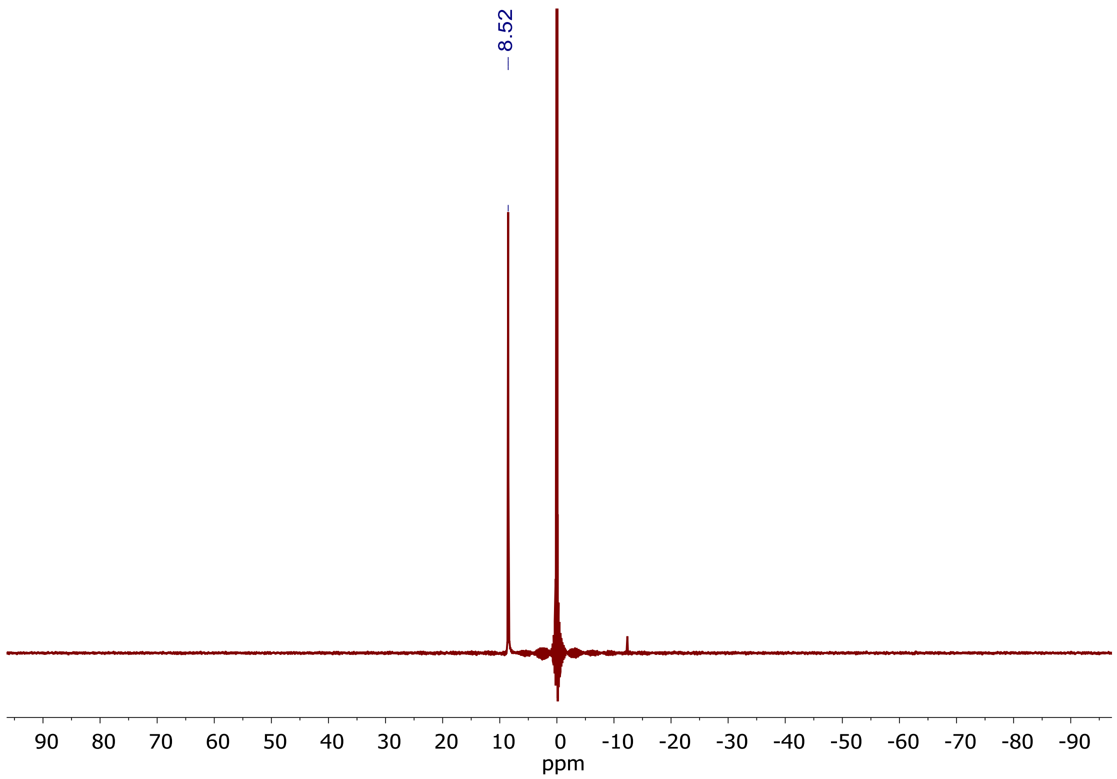

Figure S45: ^{31}P NMR (162 MHz, $CDCl_3$, 298 K, 85% H_3PO_4) of *bis*-tetrabutylammonium 2-chlorobenzyl pyrophosphonate **4**. Peak at -12.6 ppm, belongs to an impurity in the internal standard.

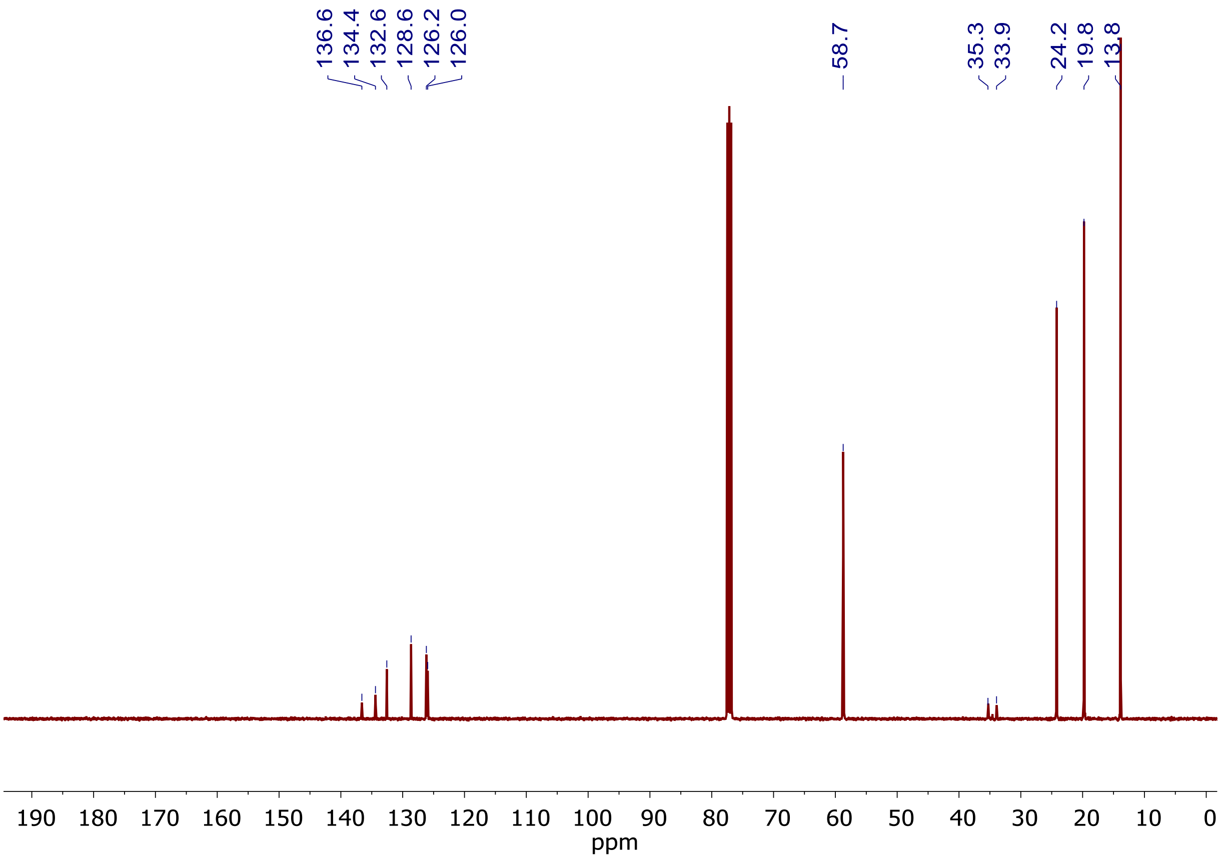

Figure S46: ^{13}C NMR (101 MHz, $CDCl_3$, 298 K) of *bis*-tetrabutylammonium 2-chlorobenzyl pyrophosphonate **4**.

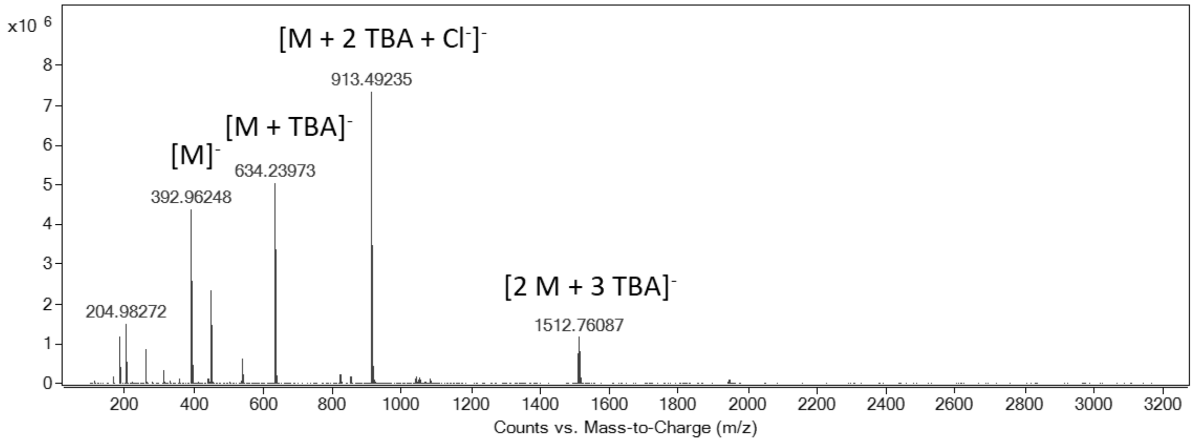

Figure S47: HRMS (ESI, negative mode) of *bis*-tetrabutylammonium 2-chlorobenzyl pyrophosphonate **4**.

Synthesis of tetrabutylammonium 2-methylbenzyl phosphonate S8

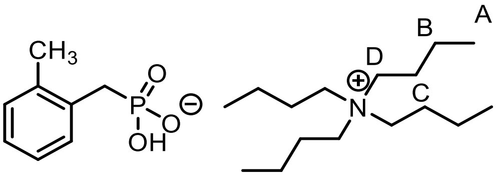

S8

300 mg (1.63 mmol) 2-methylbenzylphosphonic acid and 1.30 g (1.63 mmol, 1.0 equiv.) TBAOH · 30 H_2O were combined in a round bottom flask and dissolved in 20 mL MeOH. The mixture was stirring for 1 h at room temperature, before the solvent was removed and the product was dried in vacuo to afford 720 mg (1.63 mmol, quant.) of the desired TBA salt as a colourless solid.

^{1}H-NMR (400 MHz, $CDCl_3$): δ [ppm] = 7.42 (m, 1 H, **Ar**), 6.99 (m, 2 H, **Ar**), 6.92 (m, 1 H, **Ar**), 3.16-3.12 (m, 8 H, **D**), 2.96 (d, $^2J_{H\text{-}P}$ = 20.9 Hz, 2 H, **CH_2**), 2.44 (s, 3 H, **CH_3**), 1.64-1.46 (m, 8 H, **C**), 1.41-1.31 (m, 8 H, **B**), 0.94 (t, ^3J = 7.29 Hz, 12 H, **A**).

^{13}C-NMR (101 MHz, $CDCl_3$): δ [ppm] = 138.3 (d), 137.3 (d), 131.2 (d), 129.4 (d), 125.0 (d), 124.3 (d), 58.6, 36.2 – 34.9 (d), 24.2, 20.8, 19.8, 13.9.

^{31}P-NMR (162 MHz, $CDCl_3$, external standard: 85% H_3PO_4): δ [ppm] = 16.77.

HRMS(ESI (-)): m/z = 185.03761 (calc. 185.03730, δm/m = 1.68 ppm) $[M]^-$, 612.35852 $[2M+TBA]^-$.

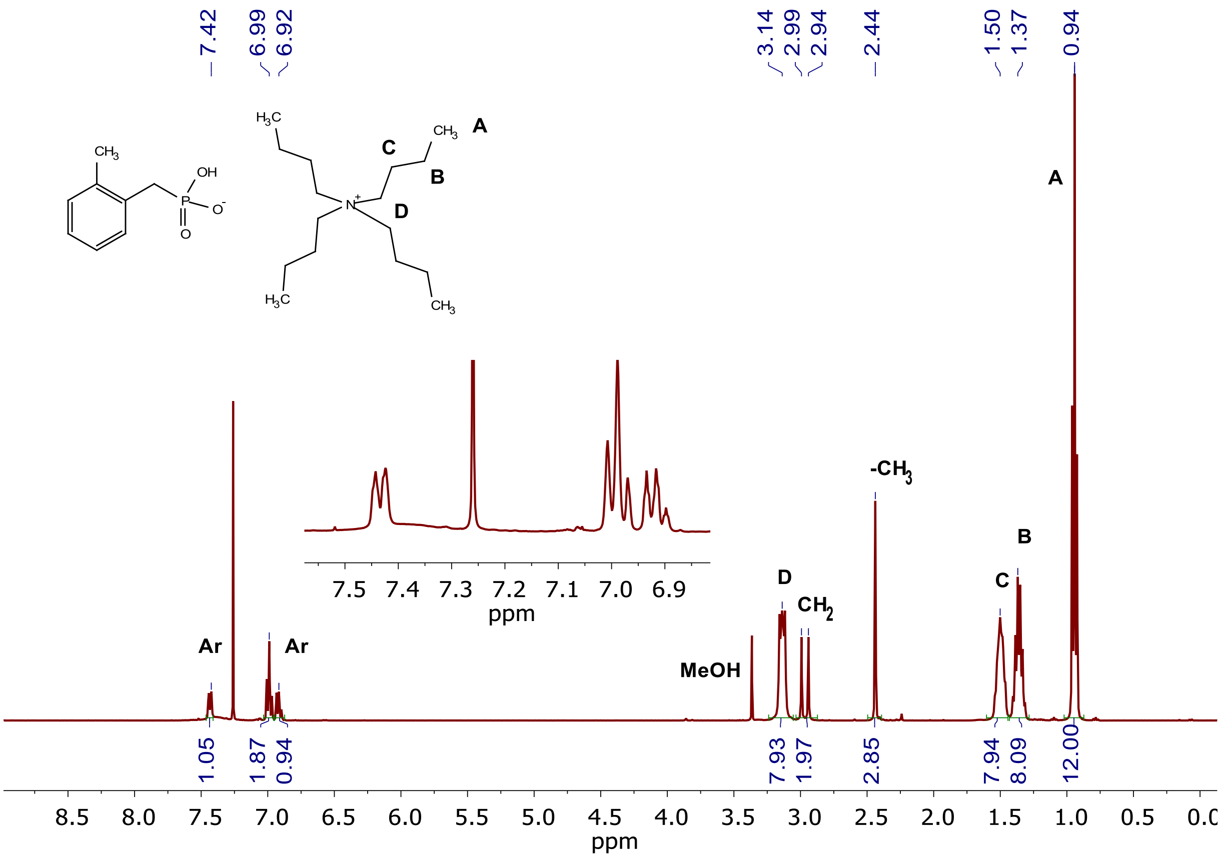

Figure S48: ^{1}H NMR (400 MHz, $CDCl_3$, 298 K) of tetrabutylammonium 2-methylbenzyl phosphonate **S8**.

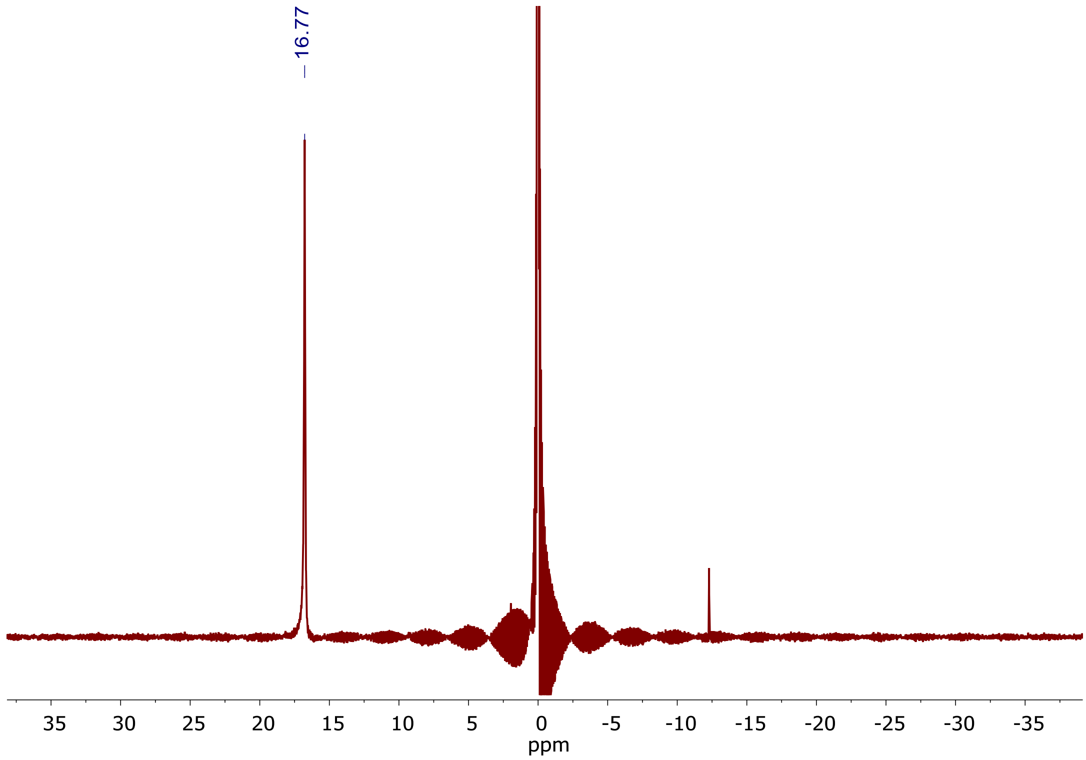

Figure S49: ^{31}P NMR (162 MHz, $CDCl_3$, 298 K, 85% H_3PO_4) of tetrabutylammonium 2-methylbenzyl phosphonate **S8**. Peak at -12.6 ppm, belongs to an impurity in the internal standard.

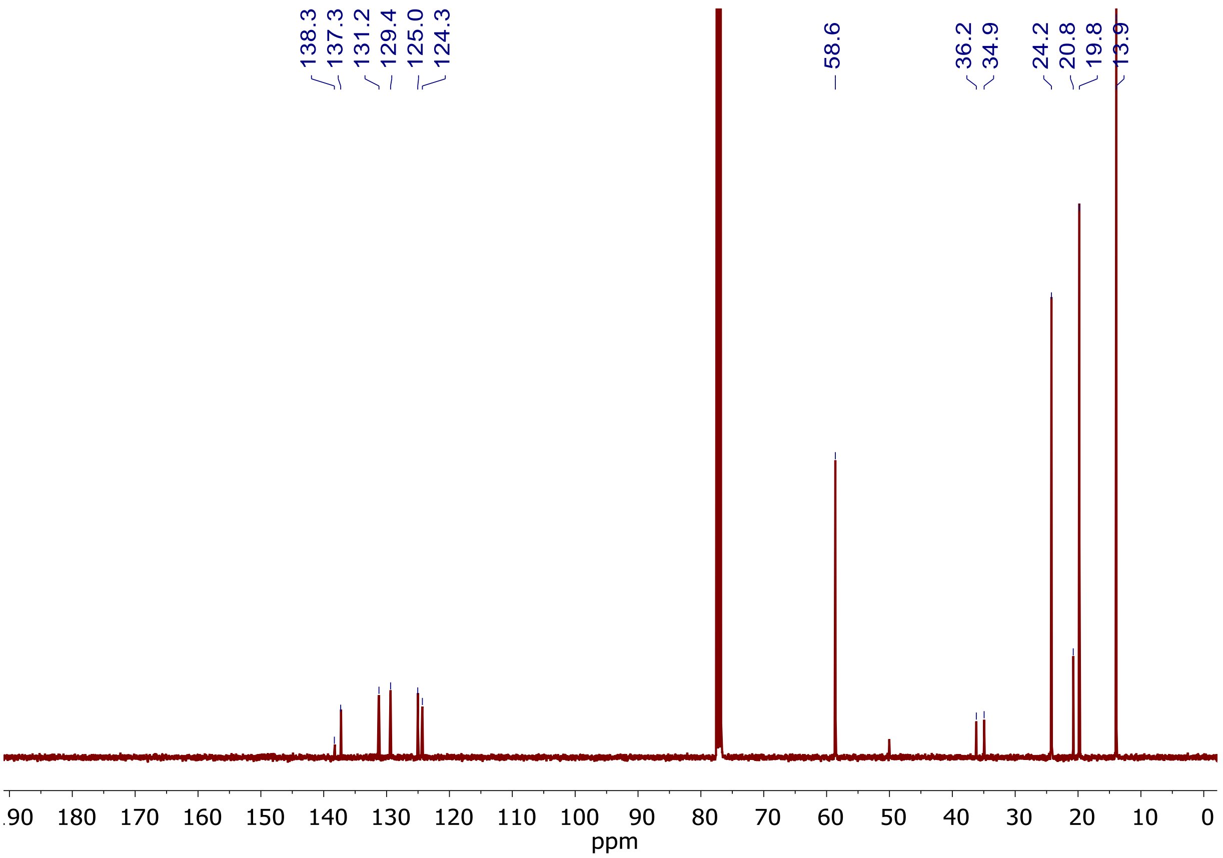

Figure S50: ^{13}C NMR (101 MHz, $CDCl_3$, 298 K) of tetrabutylammonium 2-methylbenzyl phosphonate **S8**.

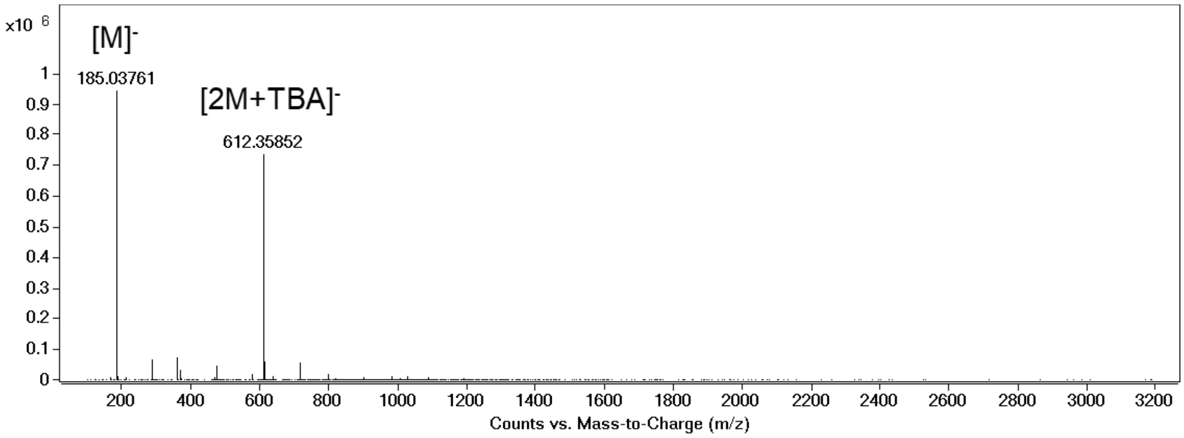

Figure S51: HRMS (ESI, negative mode) of tetrabutylammonium 2-methylbenzyl phosphonate **S8**.

Synthesis of *bis*-tetrabutylammonium 2-methylbenzyl pyrophosphonate (Me-benzyl-PP) 5

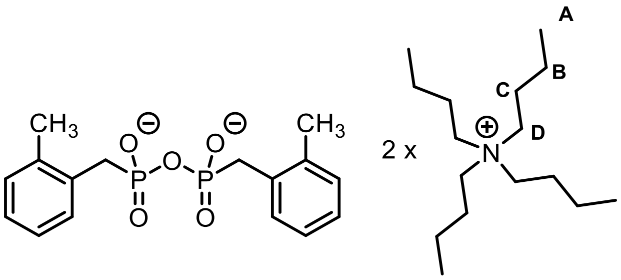

5

200 mg (0.61 mmol) 2-methylbenzyl phosphonate **S8** were dissolved in 20 mL DCM and 705 µL (4.53 mmol, 10 equiv.) *N,N'*-diisopropylcarbodiimide (DIC) were added. The mixture was stirred at room temperature for 48 h. DCM was evaporated and the residue was dissolved in 4 mL of a H_2O / MeCN (3:1) mixture. MPLC on an Interchim PF-15C18HP-F0025 C18 column with a mobile phase of MeCN and water (20-30% MeCN) and subsequent lyophilization yielded 119 mg (0.14 mmol, 46%) of the dimer as a colourless oil.

^{1}H-NMR (400 MHz, DMSO-d$_6$): δ [ppm] = 7.39 (m, 2 H, **Ar**), 6.98 – 6.87 (m, 6 H, **Ar**), 3.24 - 3.10 (m, 16 H, **D**), 2.95 – 2.89 (m, 4 H, **CH$_2$**), 2.34 (s, 3 H, **CH$_3$**) 1.59 – 1.52 (m, 16 H, **C**), 1.34 -1.26 (m, 16 H, **B**), 0.93 (t, ^3J = 7.34 Hz, 24 H, **A**).

^{13}C-NMR (101 MHz, DMSO-d$_6$): δ [ppm] = 138.8 (t), 136.3 (t), 130.6, 128.7, 124.4, 123.6, 57.5, 23.1, 20.3, 19.2, 13.5.

^{31}P-NMR (162 MHz, DMSO-d$_6$, external standard: 85% H_3PO_4): δ [ppm] = 6.45.

HRMS(ESI (-)): m/z = 353.07163 (calc. 353.07132, δm/m = 0.88 ppm) [M]$^-$, 594.34748 (calc. 594.34827, δm/m = 1.33 ppm) [2M+TBA]$^-$.

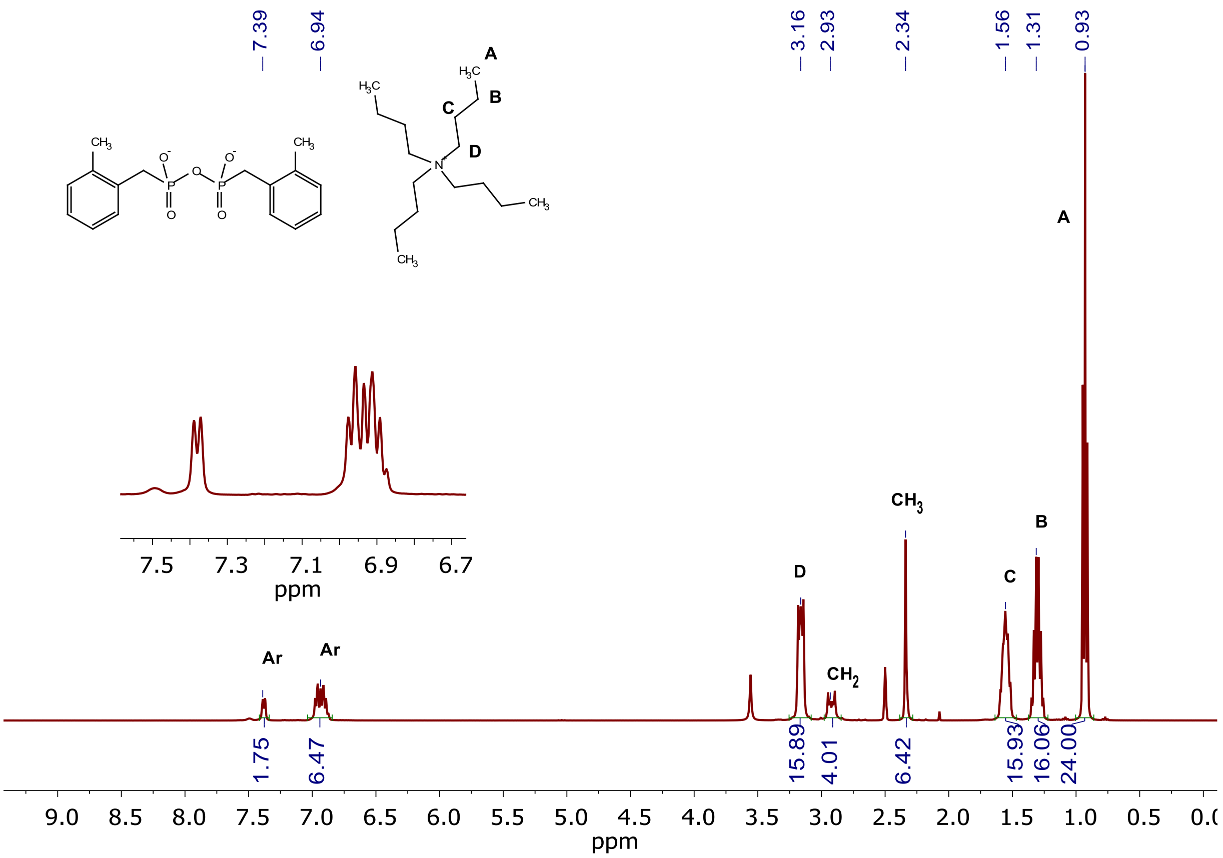

Figure S52: ^{1}H NMR (400 MHz, DMSO-d$_6$, 298 K) of *bis*-tetrabutylammonium 2-methylbenzyl pyrophosphonate **5**.

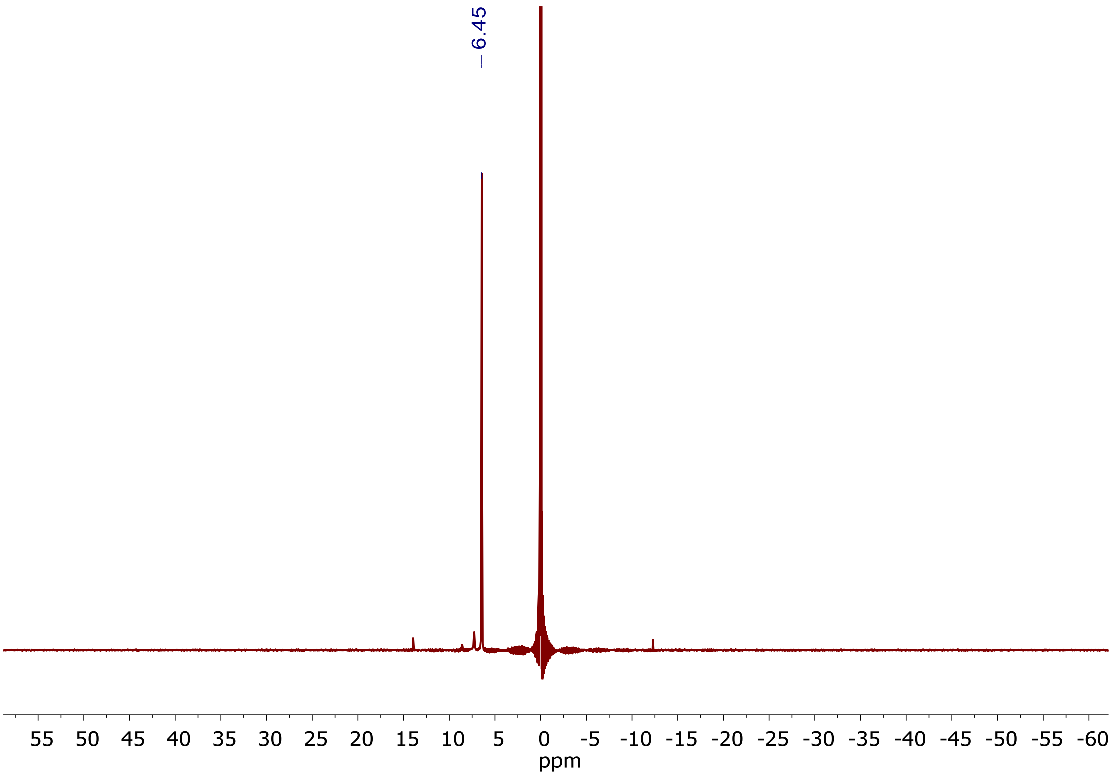

Figure S53: ^{31}P NMR (162 MHz, DMSO-d$_6$, 298 K, 85% H_3PO_4) of *bis*-tetrabutylammonium 2-methylbenzyl pyrophosphonate **5**.

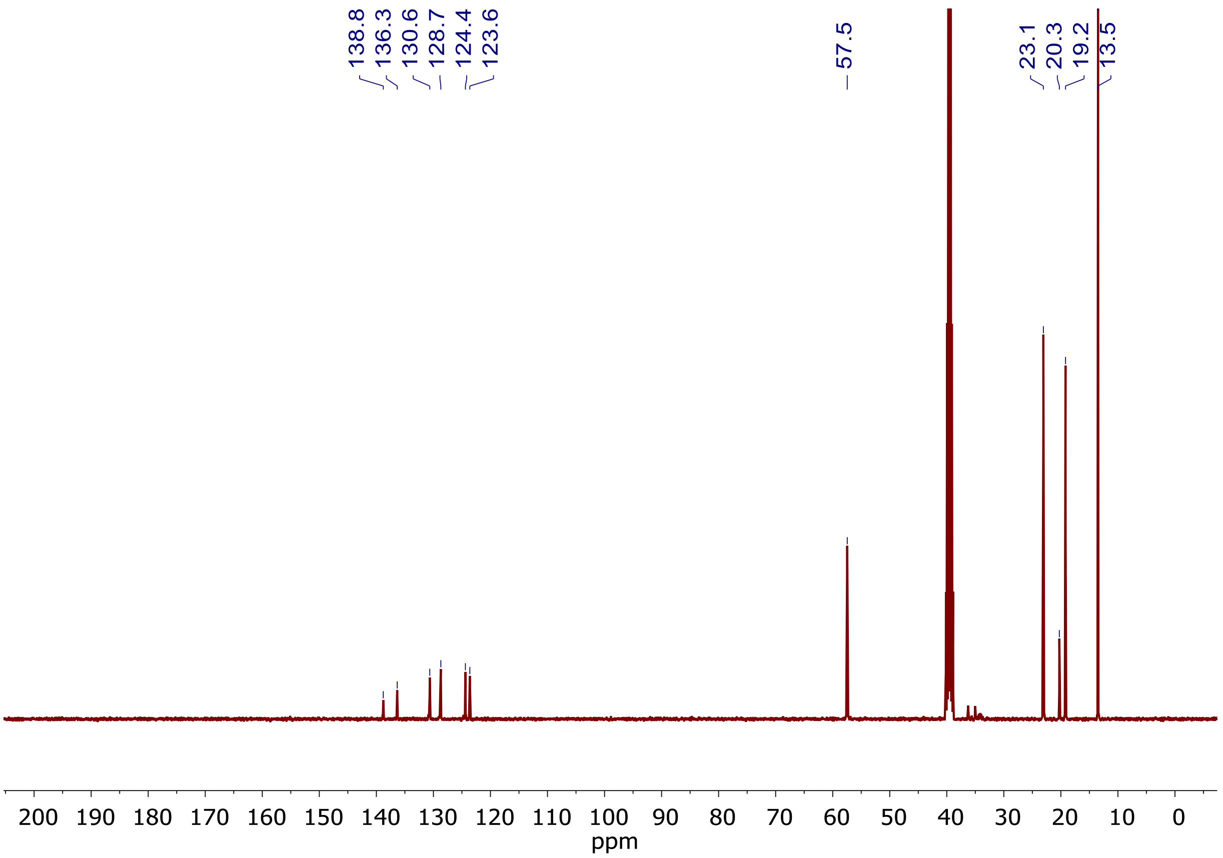

Figure S54: ^{13}C NMR (101 MHz, DMSO-d_6, 298 K) of *bis*-tetrabutylammonium 2-methylbenzyl pyrophosphonate **5**.

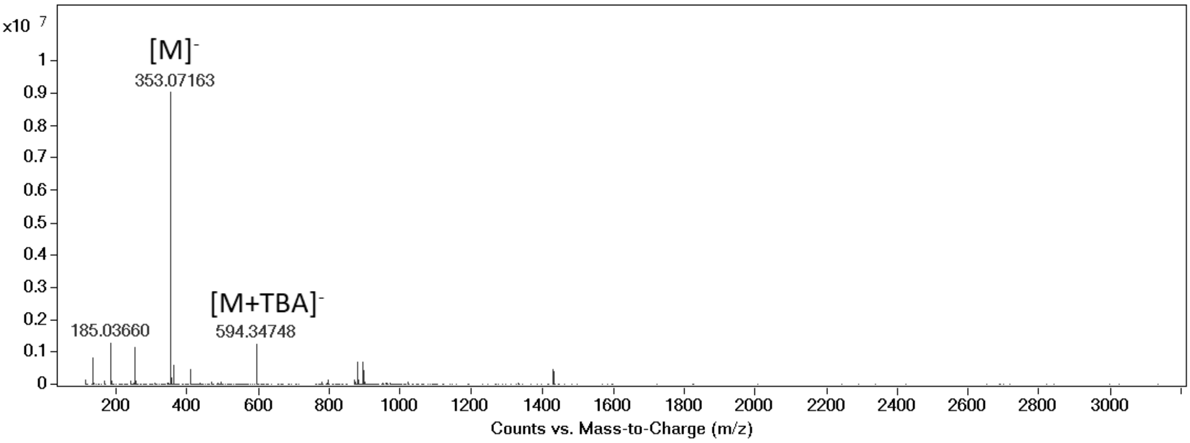

Figure S55: HRMS (ESI, negative mode) of *bis*-tetrabutylammonium 2-methylbenzyl pyrophosphonate **5**.

Synthesis of Cyanostar

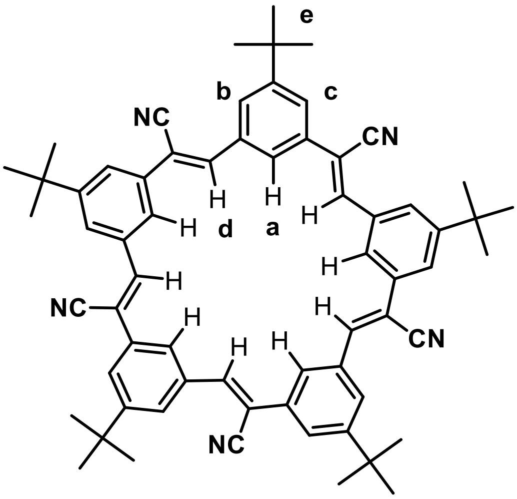

CS

Cyanostar (**CS**) was synthesized according to a published procedure.[3]

^{1}H NMR (400 MHz, CD_2Cl_2): δ [ppm] = 8.67 (s, 5 H, **b**), 7.88 (s, 5 H, **c**), 7.78 (s, 5 H, **d**), 7.70 (s, 5 H, **a**), 1.49 (s, 45 H, **e**).

^{13}C NMR (101 MHz, CD_2Cl_2): δ [ppm] = 154.3, 142.3, 135.1, 134.3, 127.2, 126.4, 124.6, 117.9, 111.3, 35.9, 31.4

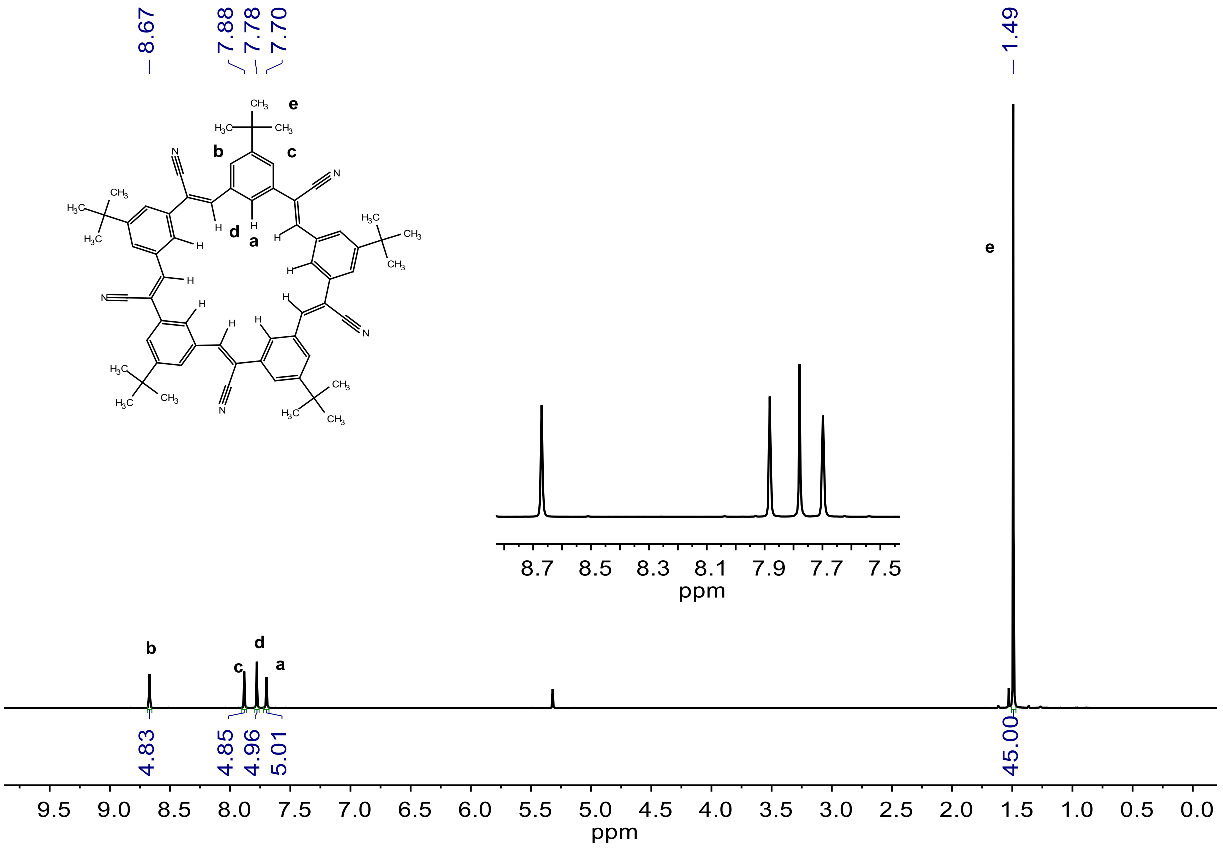

Figure S56: ^{1}H NMR (400 MHz, CD_2Cl_2, 298 K) of Cyanostar (**CS**).

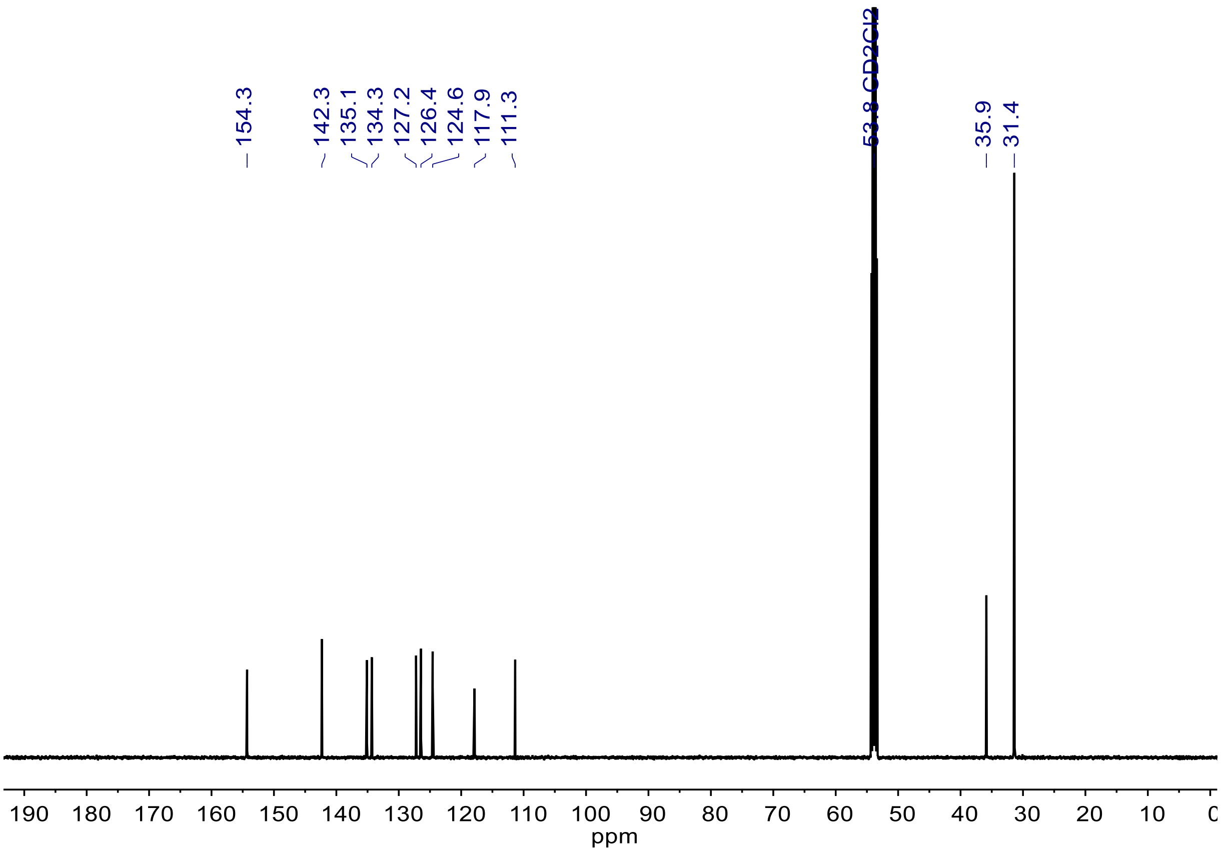

Figure S57: ^{13}C NMR (101 MHz, CD_2Cl_2, 298 K) of Cyanostar (**CS**).

NMR Kinetic Experiments

25 µL of a **CS** stock solution (20 mM in CD_2Cl_2) were diluted with 435 µL CD_2Cl_2 and an initial ^{1}H-NMR (number of scans = 16, delay = 1s) was measured. 40 µL of a stock solution (25 mM in CD_2Cl_2) of the corresponding pyrophosphonate (**PP**) were added to obtain a final concentration of 2 mM **PP** and 1 mM **CS**. A series of NMR spectra were recorded. The lack time of 120 s between addition of **PP** and the first measurement was taken into account. The experiments were performed at 298 K. In case of 2:1-binding (**1**), the peak at 7.61 ppm (corresponding to the c-proton of the meso **CS** in the [3]pseudorotaxane), and in case of 1:1-binding (**2** & **3**), the peak at 9.32 ppm (corresponding to the d-proton of **CS** in the [2]pseudorotaxane) was monitored over time.

Kinetics of the binding of CS to homoallyl-PP 1

The binding between homoallyl-PP **1** and 2 x **CS** leading to the formation of the [3]pseudorotaxane (2:1 binding) reaches its equilibrium fast (i.e. no delay time could be observed).

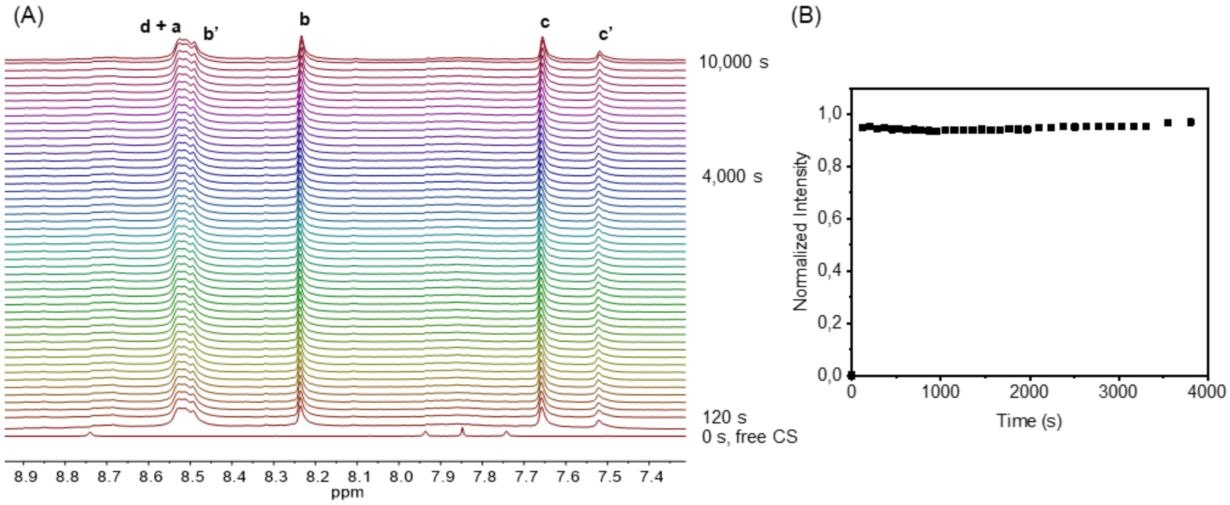

Figure S58: Binding kinetics. (A) Stacked ^{1}H NMR spectra at t=0 to t=3 h. (B) Normalized intensity of c-proton over the first 4000 s. No change can be observed, indicating that the equilibrium is reached fast (< 120 s). 298 K, DCM-d_2.

Binding Kinetics of CS on benzyl-PP 2

The binding between benzyl-PP **2** and **CS** leading to the formation of the [2]pseudorotaxane (1:1 binding) reaches its equilibrium fast (i.e. no delay time could be observed).

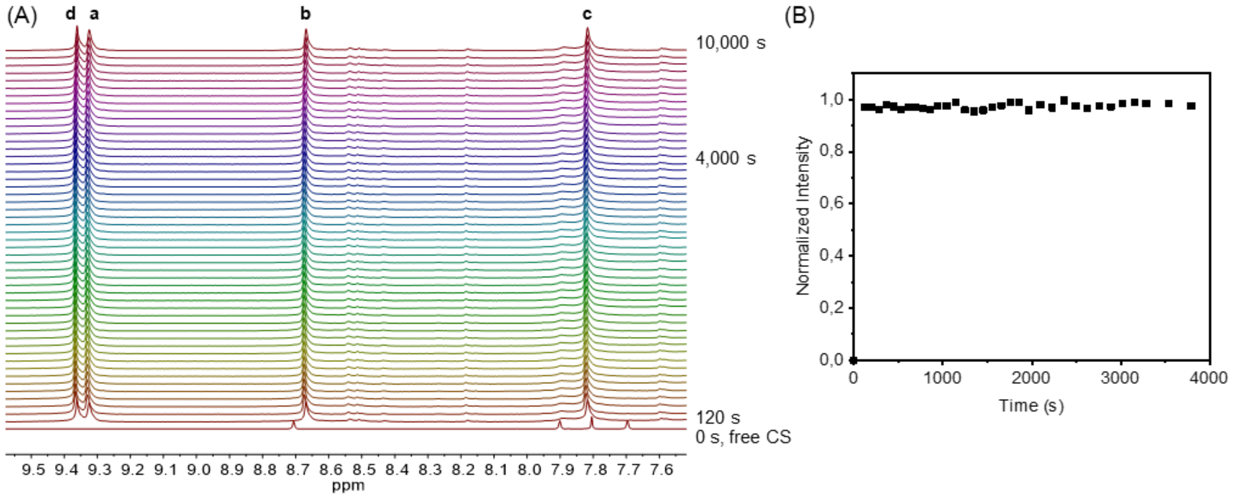

Figure S59: Binding kinetics. (A) Stacked NMR spectra at t=0 to t=3 h. (B) Normalized intensity of d-proton over the first 4000 s. No change can be observed, indicating that the equilibrium is reached fast (< 120 s). 298 K, DCM-d_2.

Binding Kinetics of CS on F-benzyl-PP 3

The binding between F-benzyl-PP **3** and **CS** leading to the formation of the [2]pseudorotaxane (1:1 binding) reaches its equilibrium within approx. 15 min.

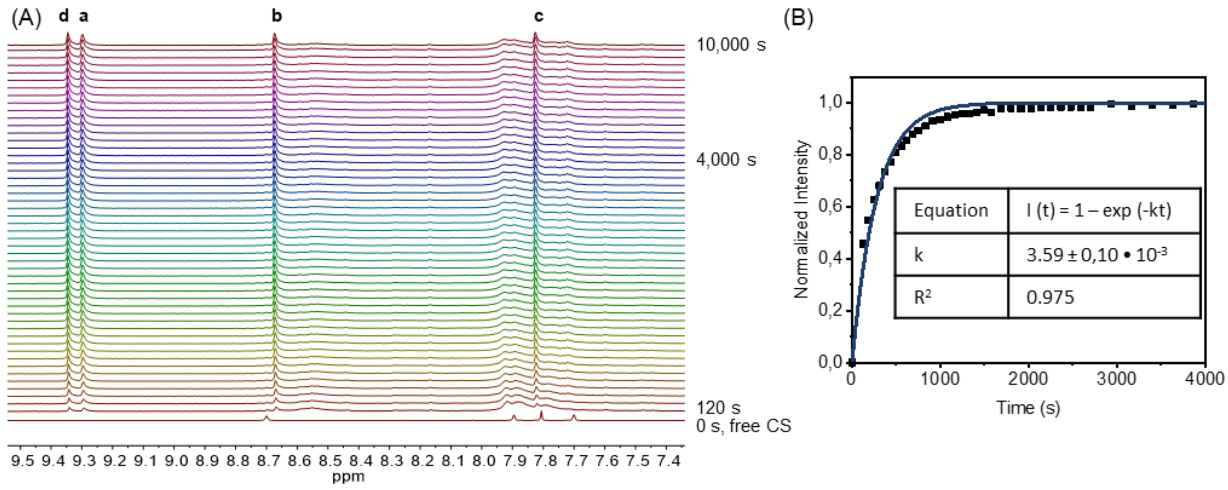

Figure S60: Binding kinetics. (A) Stacked NMR spectra at t=0 – t=3 h. (B) Normalized intensity of d-proton over the first 4000 s. The intensity increased and reached a plateau after approx. 15 min, indicating that the equilibrium is reached. Data points were fitted to a first order kinetics. 298 K, DCM-d_2.

The kinetic curve in Fig. S60B was fitted as follows: $I_t = 1 - e^{kt}$ (R^2= 0.975) and the rate constant was determined to be k_{obs} = 0.215 min^{-1} or k_{obs} = 3.59 • 10^{-3} s^{-1}. According to $t_{50} = \frac{\ln(2)}{k}$ the time where 50% of the final equilibrium of the [2]pseudorotaxane formation is reached is t_{50} = 3.22 min = 193 s.

In case of pyrophosphonates **1** and **2** (Fig. S58 and S59) the plateau was already reached after 120 s. This corresponds to five times a t_{50} of 24 s after which 97% of the plateau would be reached. This indicates that the t_{50} of formation is below 24 s.

De-threading Kinetics

Assuming that the kinetics of de-threading might be slower and therefore measurable, we prepared the 1:1 complex of benzyl phosphonate **2** and **CS** in $CDCl_3$ and triggered de-threading by addition of either $TBAPF_6$ (Fig. S61) or H_2O (Fig. S62). A mixture of 2 mM pyrophosphonate **2** and 1 mM **CS** was prepared in $CDCl_3$. After a first ^{1}H NMR measurement, 2 mM PF_6^- or 30 mM H_2O, respectively were added and further NMR spectra were recorded.

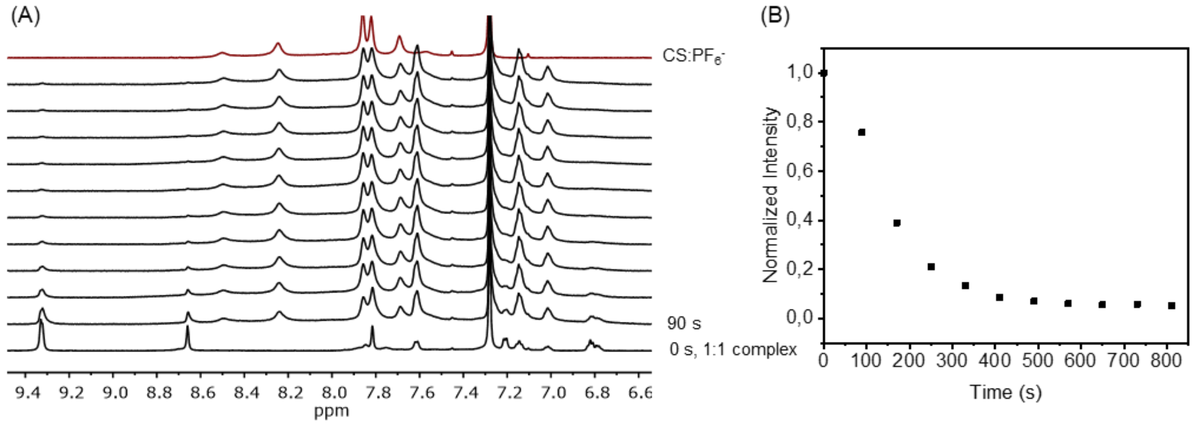

Figure S61: De-threading kinetics triggered by $TBAPF_6$ addition. (A) Stacked NMR spectra at t=0 – t= 800 s. (B) Normalized intensity of d&a-protons. The intensity increased and reached a plateau after approx. 6 min after addition of $TBAPF_6$, indicating that equilibrium is reached. A half life of ca. 120 s was determined (please note that PF_6^- is likely involved in the de-threading mechanism). 298 K, $CDCl_3$.

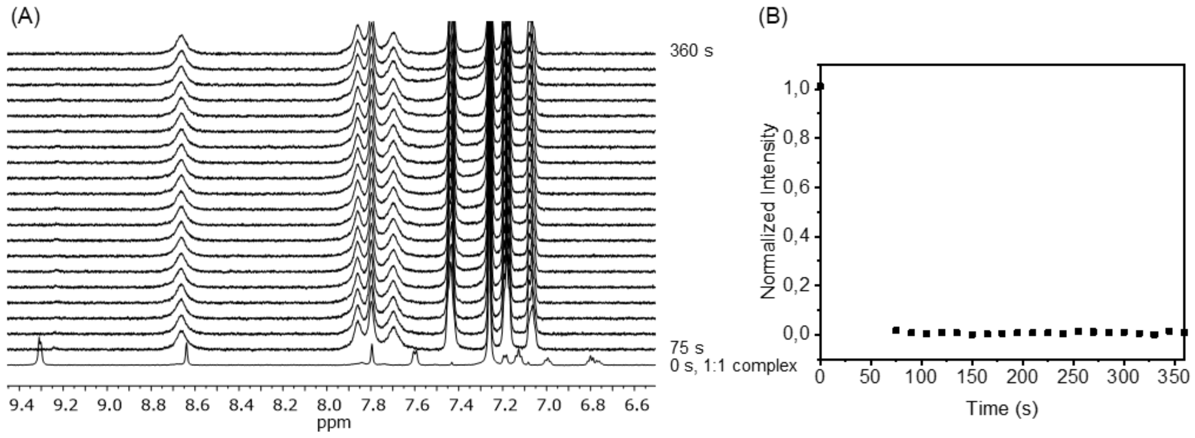

Figure S62: De-threading kinetics triggered by addition of H_2O. (A) Stacked NMR spectra at t=0 – t= 360 s. (B) Normalized intensity of d&a-proton. The signals of the 1:1 complex vanished after addition of H2O already at the first measurement after 75 s. 298 K, $CDCl_3$.

NMR Variable Temperature (VT) Experiments

A series of VT ^{1}H NMR spectra of free **CS** under the same conditions as below were measured for comparison. 25 µL of a 20 mM **CS** stock solution were diluted with 475 µL $CDCl_3$ to give a 1 mM **CS** solution. VT ^{1}H NMR spectra (number of scans = 16, delay = 1 s) were measured starting from the lower temperature.

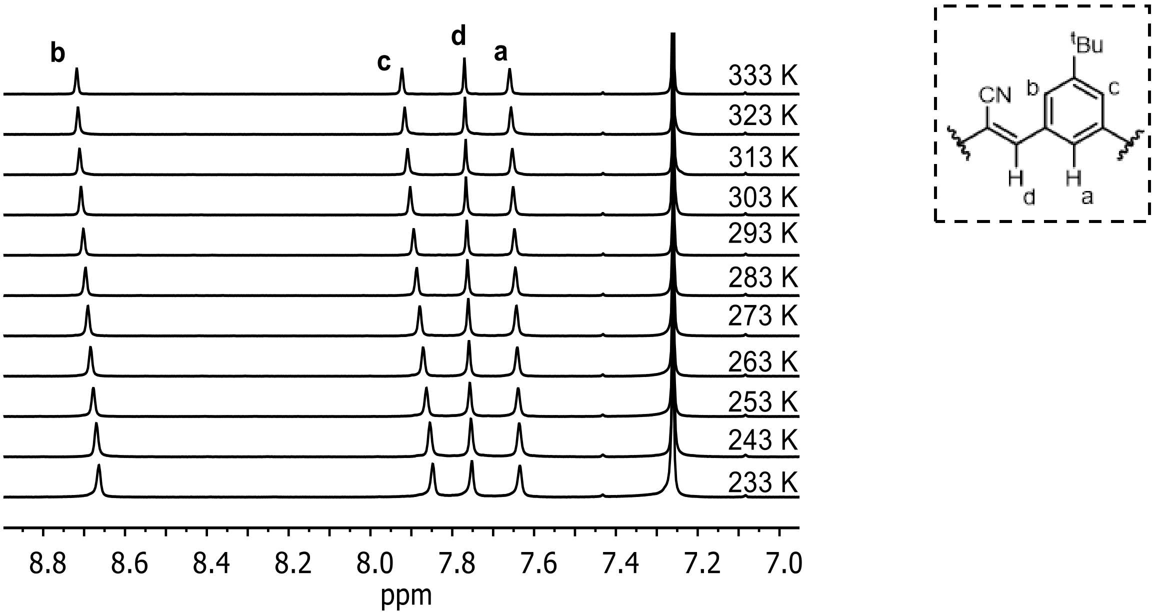

Figure S63: VT ^{1}H NMR spectra ($CDCl_3$) of **CS**.

VT ^{1}H NMR spectra of CS – PP complexes

General procedure. 25 µL of a 20 mM **CS** stock solution (in $CDCl_3$) and 40 µL of a 25 mM stock solution of the corresponding pyrophosphonate (in $CDCl_3$) were diluted to obtain a total volume of 500 µL (1 mM **CS** + 2 mM **PP**). VT ^{1}H NMR spectra (number of scans = 16, delay = 1s) were measured starting from the lower temperature. The temperature was equilibrated for 5 min before each measurement. Signals of the free and the perched **CS** are depicted in cyan and aromatic signals of the pyrophosphonates are grey. The signals of the [2]pseudorotaxane are blue and the meso and chiral signals (see Fig. S67) of [3]pseudorotaxane are depicted in green.

VT NMR spectra of the CS / homoallyl-PP 1 complex

Note: Study was performed in DCM-d_2 rather than $CDCl_3$.

At lower temperatures the d and a signal of [2]pseudorotaxane (blue) sharpen up (see magnification), indicating fast exchange on the NMR time scale at room temperature (where the signals are broad). Most of the **CS** is bound in the [3]pseudorotaxane (green), where only the d and a signal sharpen upon warming, while the b and c signals remain mostly unchanged.

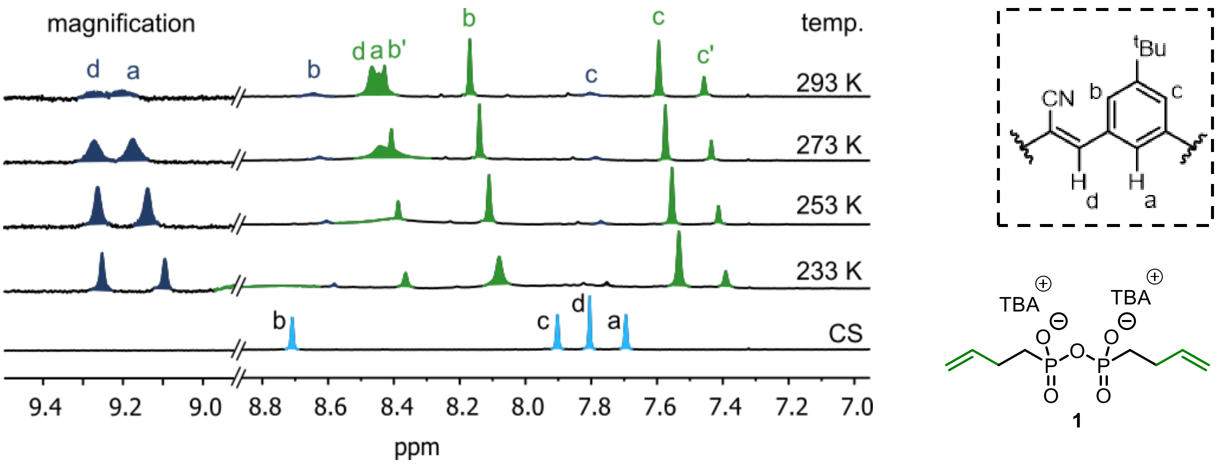

Figure S64: VT NMR spectra (DCM-d_2) of a mixture of **CS** and homoallyl-PP **1**.

VT NMR spectra of the CS / F-benzyl-PP 3 complex

The signals of the [2]pseudorotaxane (blue) shift upon temperature variation (especially signal a), but do not change otherwise. At higher temperatures (333 K) the perched interaction is (mostly) overcome and only four signals of the free **CS** remain (cyan). At 233 K, on the other hand, all these signals split into two, indicating that the perched interaction is slowed down with reference to the NMR time scale.

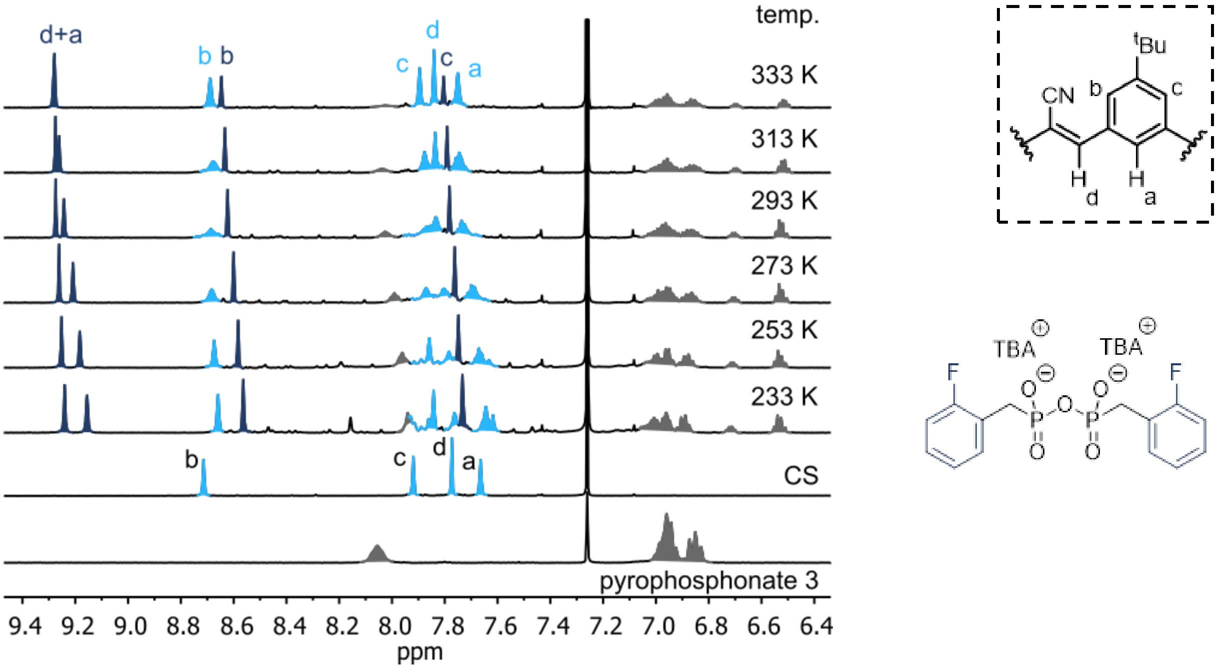

Figure S65: VT NMR spectra ($CDCl_3$) of a mixture of **CS** and F-benzyl-PP **3**.

VT NMR of CS with Cl-benzyl-PP 4

VT NMR was performed in $CDCl_3$ up to 333 K and in $C_2D_2Cl_4$ up to 403 K. Only the broad signals of the free and perched **CS** (cyan), that sharpen as the weak perched interaction is overcome, can be observed. This indicates that even at higher temperatures threading is not occurring.

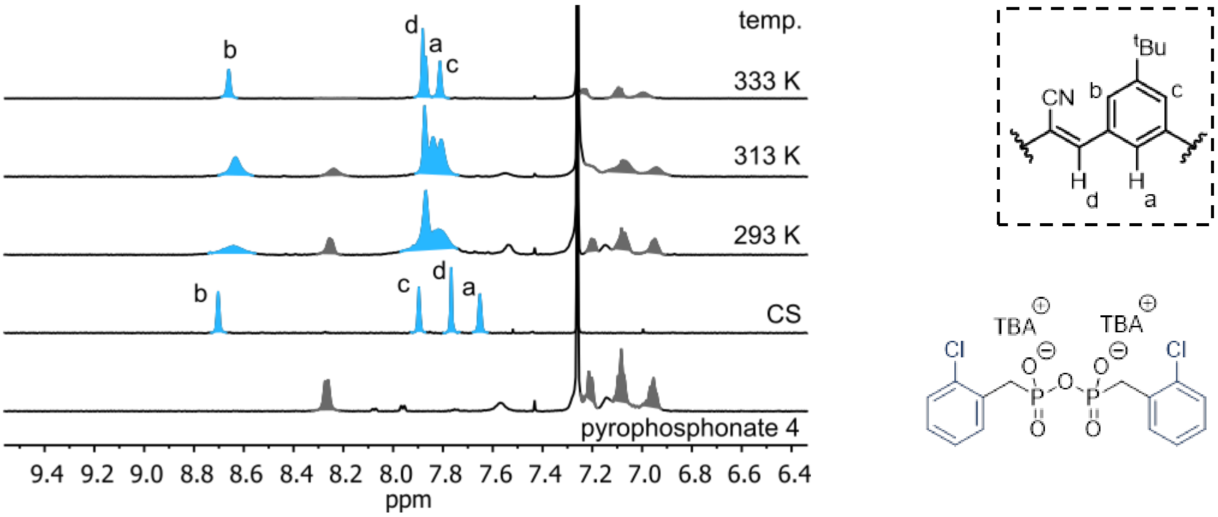

Figure S66: VT NMR spectra ($CDCl_3$) of a mixture of **CS** and Cl-benzyl-PP **4**. No threading was observed.

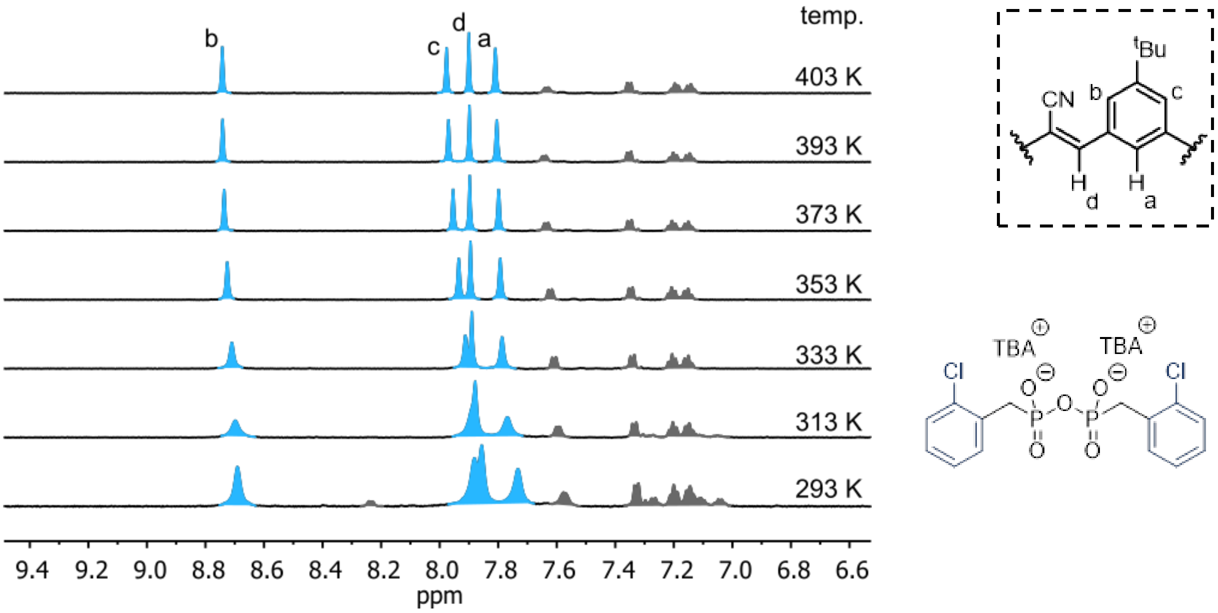

Figure S67: VT NMR spectra ($C_2D_2Cl_4$) of a mixture of **CS** and Cl-benzyl-PP **4**. No threading was observed.

VT NMR of CS with Me-benzyl-PP 5

Only the broad signals of the free and perched **CS** (cyan), that sharpen up as the perched interaction is reduced, can be observed. This indicates that even at higher temperatures threading is not occurring.

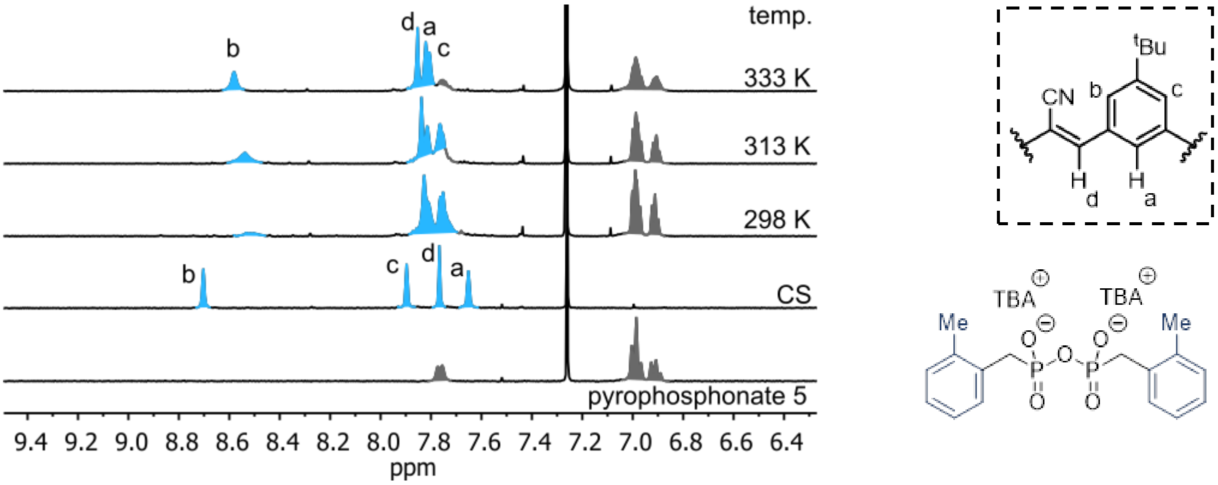

Figure S68: VT NMR ($CDCl_3$) of a mixture of **CS** and Me-benzyl-PP **5**. No threading was observed.

^{1}H NMR Titrations

12.5 µL of a 20 mM **CS** stock solution (in DCM-d$_2$) was diluted to 500 µL with DCM-d$_2$ to obtain a 0.5 mM solution (0.25 µmol **CS**). A freshly prepared stock solution of the corresponding pyrophosphonate (25 mM, DCM-d$_2$) was added in a stepwise titration (1 µL = 0.1 equiv.). The solutions were equilibrated according to their binding kinetics (see p. S47-49) before ^{1}H NMR spectra were measured (number of scans = 16, delay time = 1 s).

On one hand, the characteristic signals of the binding of **two CS** to the pyrophosphonate can be observed (depicted in green). They split into two sets of signals, because the **CS dimer** can arrange both in a chiral (‘) and in a *meso* isomer, as published previously[2,4] and depicted in Figure S67.

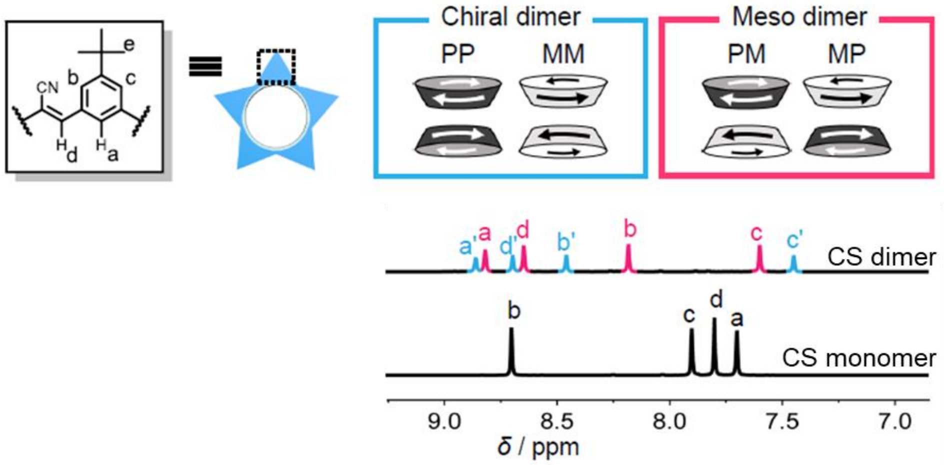

Figure S69: Visual representation of the formation of a chiral and a meso **CS** dimer and the resulting peaks in ^{1}H NMR spectra (DCM-d$_2$), compared to a free **CS** monomer. Adapted from Ref. [4].

On the other hand, a set of signals (depicted in blue) forms that corresponds to the binding of **one CS** on the pyrophosphonate (1:1 binding). The signals of free and perched cyanostar **CS** are depicted in cyan, aromatic signals of the pyrophosphonate are grey.

Additional data on the binding titration of CS to homoallyl-PP 1

To see, whether the equilibrium can be further pushed from the [3]pseudo- towards the [2]pseudorotaxane, more equivalents of homoallyl-PP **1** were added (as depicted in Fig. 2 of the manuscript). At 50 equiv. there is only little amount of the [3]pseudorotaxane left.

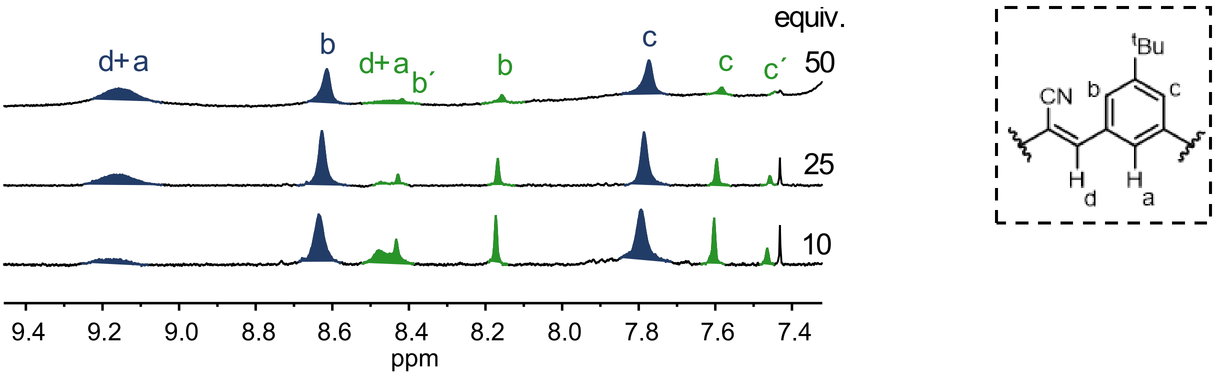

Figure S70: Additional data on the binding titration as depicted in Fig. 2 of the manuscript. Increasing the equivalents of homoallyl-PP **1**, further shifts the equilibrium from [3]pseudorotaxane (green) to [2]pseudorotaxane (blue).

While the addition **CS** to benzyl and F-benzyl PP **2** and **3** led to the formation of a 1:1 complex and a downfield shift of the corresponding ^{31}P NMR signal (see Fig. S77), an upfield shift of the ^{31}P NMR signal was observed upon formation of the 2:1 complex in case of homoallyl-PP **1**. The signal also broadened significantly, which is consistent with the broadening of the inner protons of the cyanostar macrocycle in the ^{1}H NMR spectrum (see Fig. 2B in the manuscript).

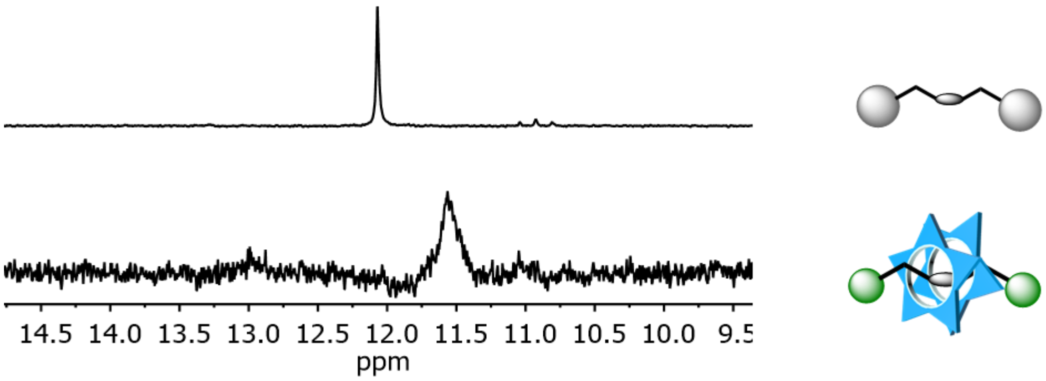

Figure S71: ^{31}P NMR (243 MHz, $CDCl_3$, 298 K) of free homoallyl-PP **1** and the 2:1 complex with **CS**.

Water-Sensitivity of binding

The free energy of hydration of phosphates is reported to be relatively high, compared to other anions.[5] It is likely that this is also true for pyrophosphonates. At the same time, binding of **CS** to pyrophosphonates is governed by hydrogen bonding. This experiment shows whether competitive hydrogen bonding of water interferes with the binding investigated in this study.

A 1 mM solution of **CS** and 2 equiv. F-benzyl-PP **3** (dried under vacuum for 48 h) was prepared in $CDCl_3$ (500 µL, stored over molecular sieves) and equilibrated for 15 min. A ^{1}H NMR spectrum was measured before and after addition of 10 µL (2% v.v.) of water (saturating chloroform).

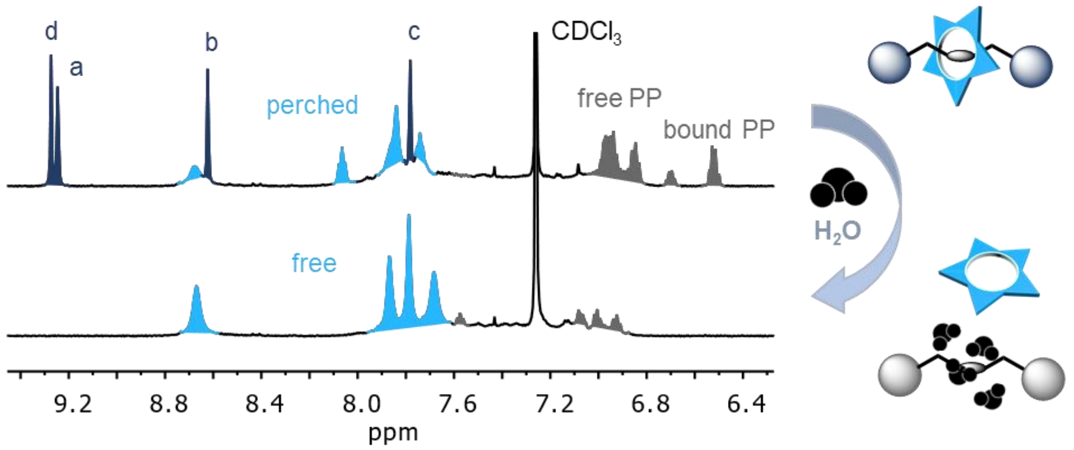

Figure S72: ^{1}H NMR spectra (in $CDCl_3$) of typical 1:1 binding (blue signals) under anhydrous conditions and after addition of 2% v.v. of water, indicating that water is shutting down the binding completely.

NOESY NMR spectrum of [2]pseudorotaxane

A 2:1 mixture of F-benzyl-PP **3** (10 mM) and **CS** (5 mM) in $CDCl_3$ was prepared and equilibrated for 15 minutes. A ^1H-^1H-NOESY NMR spectrum (number of scans = 32, mixing time = 0.3 s) was measured.

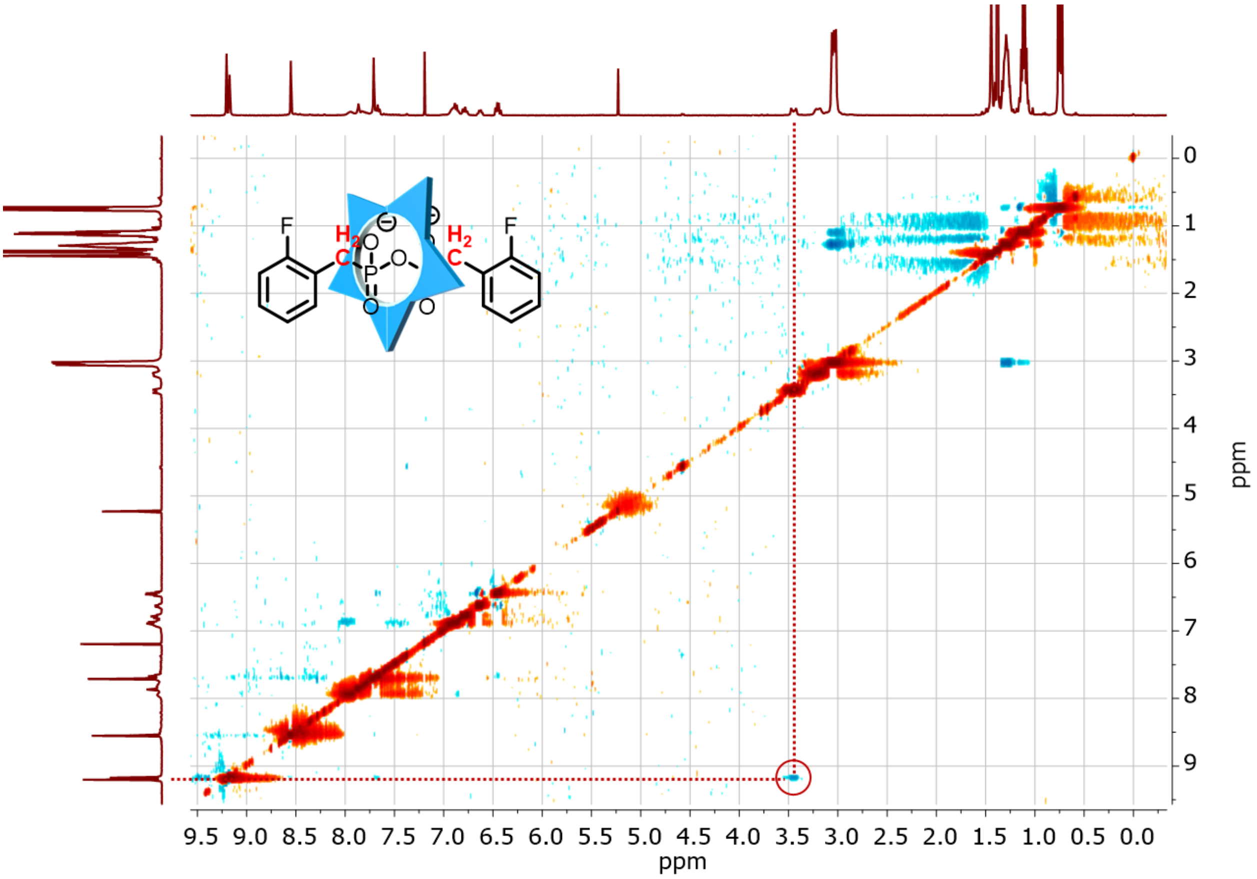

Figure S73: ^1H-^1H-NOESY spectrum of the [2]pseudorotaxane of F-benzyl-PP **3** and **CS**.

Conclusion: The cross peak between the d and a signals of the **CS** (~ 9.35 ppm) in the [2]pseudorotaxane and the benzyl signals of the bound pyrophosphonate (~ 3.45 ppm) corroborates the formation of the [2]pseudorotaxane.

Mass Spectrometry (MS) Experiments

A 200 µM solution of a 1:1 mixture of **CS** and the pyrophosphonate was prepared in HPLC-grade DCM and high-resolution mass spectrometry (HRMS) was measured with electrospray ionization (ESI) in the negative mode. Additional signals can be assigned as follows: 950.49 = CS + Cl^-; 1867.02 = 2 CS + Cl^-; 1877.05 = 2 CS + HCO_2^-; 1891.06 = 2 CS + OAc^-.

The measured isotopic patterns of the [3]pseudorotaxane (in case of homoallyl-PP **1**) and the [2]pseudorotaxane (in case of the F-benzyl-PP **3**) were compared to the calculation to corroborate their formation. mMass software[6] was used for the simulation of the isotopic patterns.

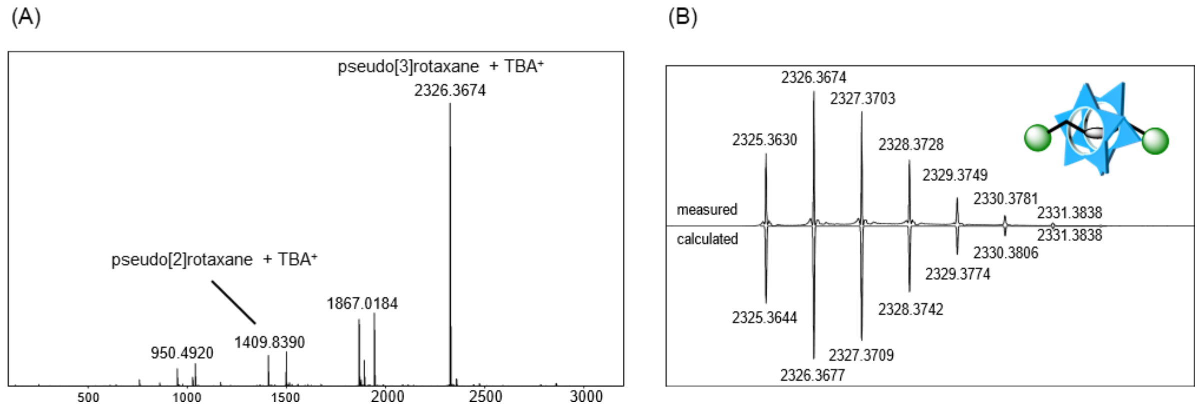

Figure S74: (A) Mass spectrum of a 1:1 mixture of homoallyl-PP **1** and **CS**. (B) Measured vs. calculated spectrum of the corresponding [3]pseudorotaxane (+TBA^+).

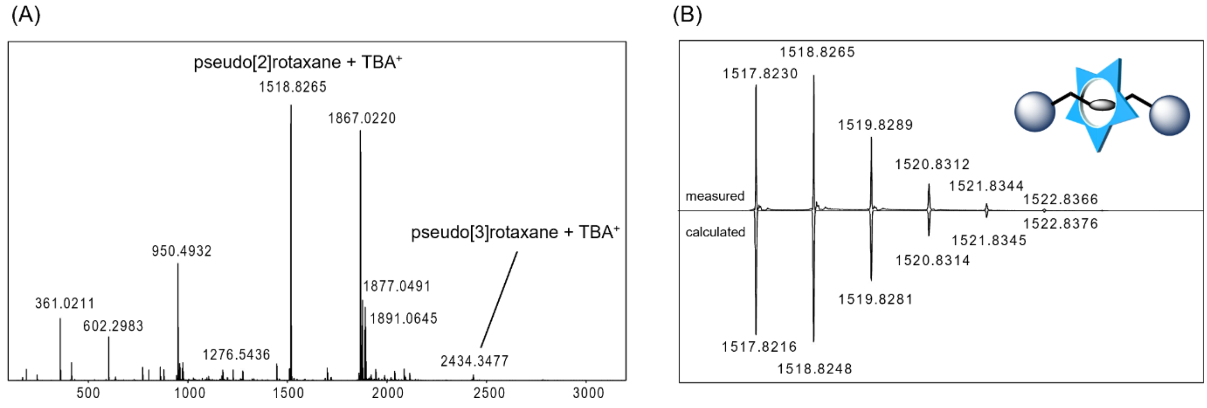

Figure S75: (A) Mass spectrum of a 1:1 mixture of F-benzyl-PP **3** and **CS**. (B) Measured vs. calculated spectrum of the corresponding [2]pseudorotaxane (+ TBA^+).

With [2]pseudorotaxane (H^+ instead of TBA^+) of **3** and **CS**, a 2D-MS experiment was performed. A collision voltage (0 – 30 V) was applied to the species in gas phase. With increasing collision voltage, the signal intensity (1276.54) reduces in favor of the free pyrophosphonate (361.02) as the **CS** is gradually slipped off. This behaviour is typical for a pseudorotaxane.

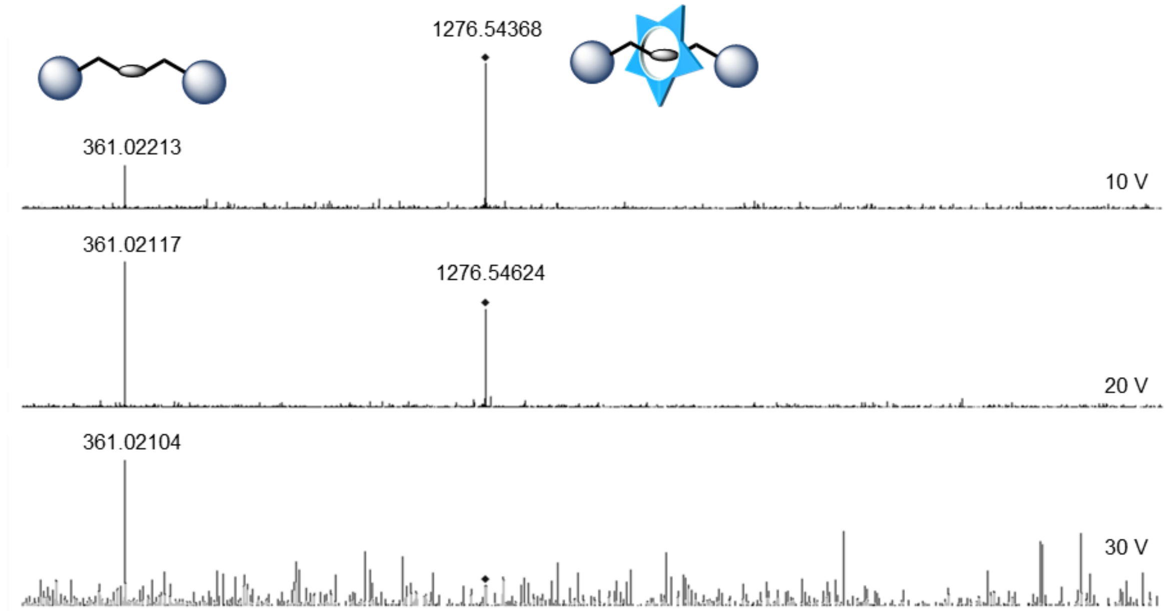

Figure S76: 2D-MS experiment with [2]pseudorotaxane of benzyl-PP **3** and **CS**. The signal of the [2]pseudorotaxane (1276.54) decreases with increasing collision voltage.

^{31}P NMR titrations and binding constants

Titrations of **CS** to the corresponding pyrophosphonate were performed. In all titration experiments, the peak of the free pyrophosphonate (G, grey) decreased upon addition of **CS** (H) in the ^{31}P NMR spectrum. It also broadend up, indicating the perched interaction which is fast on the NMR time scale. At the same time, a new peak (HG, blue) of the [2]pseudorotaxane increased. It is sharp, as the threading/dethreading is slow on the NMR time scale. The concentrations of free pyrophosphonate and [2]pseudorotaxane can be obtained by integration of the NMR peaks under the reasonable assumption that the **exchange is slow on the NMR time scale**. (We further assumed no further species, signal overlap or decomposition.) The concentration of the free **CS** can be calculated. According to the mass action law, the binding constants can be calculated as follows:

$$K_A = \frac{[HG]}{[G] * [H]} = \frac{[pseudorotaxane]}{[pyrophosphonate]*[CS]} .$$

Binding constant of CS to benzyl-PP 2

500 µL of a 2.5 mM solution of benzyl-PP **2** was prepared in $CDCl_3$. ^{31}P NMR spectra (number of scans = 64, delay time = 3 s) were measured. A 50 mM solution of **CS** in $CDCl_3$ was added in a stepwise titration (5 µL = 0.2 eq **CS**). In $CDCl_3$ no formation of the [3]pseudorotaxane was observed (see Fig. S78), as the interaction between the pyrophosphonate and the TBA counterion is stronger in the less polar solvent $CDCl_3$, favoring the 1:1 binding mode. Furthermore, in the more polar solvent CD_2Cl_2, the cyanostar macrocycle is more prone to dimerize due to the solvophobic effect.

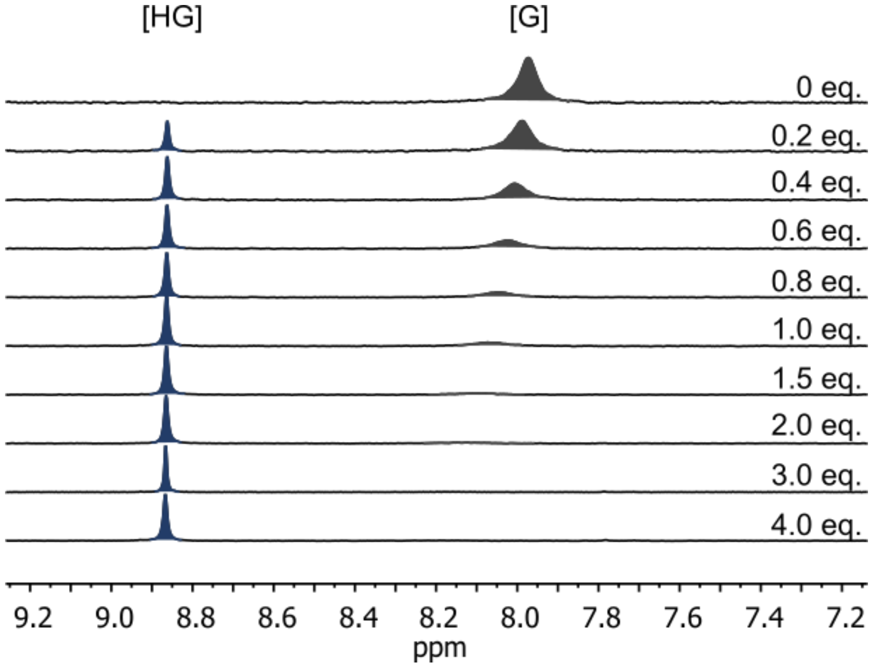

Figure S77: ^{31}P NMR titration of **CS** to pyrophosphonate **2** (2.5 mM, $CDCl_3$).

Table S1: ^{31}P NMR titration. Ratios of G and HG were determined by NMR integration, directly giving the amounts of G, HG and H and the corresponding concentrations by considering the total volume. K_A values were determined according to the mass action law.

**Equiv. CS**	**0.0**	**0.2**	**0.4**	**0.6**	**0.8**	**1.0**	**1.5**	**2.0**	**3.0**	**4.0**
Total volume	500	505	510	515	520	525	537,5	550	575	600
	**free pyrophosphonate (G)**									
Ratio	1.00	0.84	0.68	0.55	0.44	0.33	0.17	0.12	0.05	0.04
Amount [µmol]	1.25	1.05	0.85	0.68	0.55	0.41	0.21	0.14	0.07	0.05
Conc. [mM]	2.50	2.07	1.66	1.33	1.05	0.78	0.40	0.26	0.12	0.09
	**[2]pseudorotaxane (HG)**									
Ratio	0.00	0.16	0.32	0.45	0.56	0.67	0.83	0.89	0.95	0.96
Amount [µmol]	0.00	0.21	0.40	0.57	0.70	0.84	1.04	1.11	1.18	1.20
Conc. [mM]	0.00	0.41	0.79	1.10	1.35	1.60	1.93	2.01	2.06	1.99
	**free CS (H)**									
Amount [µmol]	0.00	0.05	0.10	0.18	0.30	0.41	0.84	1.39	2.57	3.81
Conc. [mM]	0.00	0.09	0.19	0.35	0.57	0.78	1.56	2.53	4.47	6.34
	**K_A [M^{-1}]**									
$\frac{[HG]}{[G] * [H]}$	-	2200	2440	2350	2260	2640	3110	3040	3920	3510

The last two data points were left out as the integration accuracy decreased. K_A was determined as the mean value of the first seven data points with the standard deviation as error to be:

$\boldsymbol{K_A = 2.58 \pm 0.34 \cdot 10^3\ M^{-1}}$

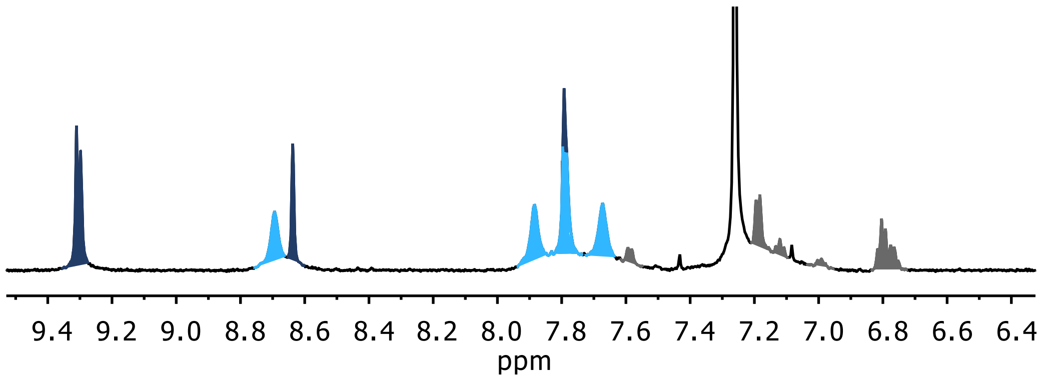

Figure S78: ^{1}H NMR spectrum of a mixture of 1 mM **CS** and 0.5 mM benzyl-PP **2** in $CDCl_3$ at 298 K. No signals of [3]pseudorotaxane are observed.

Binding constant of CS to F-benzyl-PP 3 (in $CDCl_3$)

500 µL of a 5.0 mM solution of F-benzyl-PP **3** was prepared in $CDCl_3$. ^{31}P NMR (number of scans = 64, delay time = 3 s) was measured. A 50 mM solution of **CS** in $CDCl_3$ was added in a stepwise titration (10 µL = 0.2 eq **CS**). The mixture was equilibrated for 15 min before measurement to allow the binding to equilibrate (compare Fig. S60).

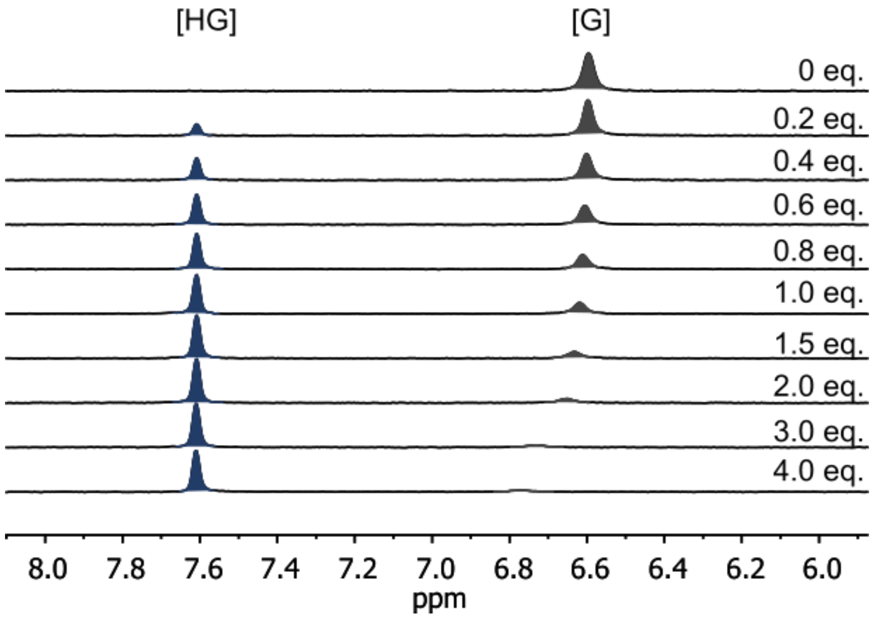

Figure S79: ^{31}P NMR titration of **CS** to F-benzyl-PP **3** (5.0 mM, $CDCl_3$).

Table S2: ^{31}P NMR titration in $CDCl_3$. Ratios of G and HG were determined by NMR integration, directly giving the amounts of G, HG and H and the corresponding concentrations by considering the total volume. K_A values were determined according to the mass action law.

**Equiv. CS**	**0.0**	**0.2**	**0.4**	**0.6**	**0.8**	**1.0**	**1.5**	**2.0**	**3.0**	**4.0**
Total volume	500	510	520	530	540	550	575	600	650	700
	**free pyrophosphonate (G)**									
Ratio	1.00	0.83	0.66	0.52	0.42	0.35	0.24	0.18	0.12	0.10
Moles [µmol]	2.50	2.08	1.65	1.31	1.05	0.87	0.59	0.46	0.30	0.24
Conc. [mM]	5.00	4.08	3.18	2.46	1.94	1.57	1.03	0.76	0.46	0.34
	**[2]pseudorotaxane (HG)**									
Ratio	0.00	0.17	0.34	0.48	0.58	0.65	0.76	0.82	0.88	0.91
Moles [µmol]	0.00	0.42	0.85	1.20	1.46	1.64	1.91	2.05	2.20	2.26
Conc. [mM]	0.00	0.82	1.63	2.25	2.69	2.97	3.32	3.41	3.39	3.23
	**free CS (H)**									
Moles [µmol]	0.00	0.08	0.15	0.31	0.55	0.87	1.84	2.96	5.30	7.74
Conc. [mM]	0.00	0.16	0.29	0.58	1.01	1.57	3.20	4.93	8.15	11.1
	**K_A [M^{-1}]**									
$\frac{[HG]}{[G] * [H]}$	-	1240	1750	1590	1380	1200	1010	913	908	862

The last two data points were left out as the integration accuracy decreased. K_A was determined as the mean value of the first seven data point with the standard deviation as error to be:

$K_A = 1.30 \pm 0.28 \cdot 10^3$ M^{-1}

Binding constant of CS to F-benzyl-PP 3 (in DCM-d_2)

500 µL of a 2.5 mM solution of F-benzyl-PP **3** was prepared in $CDCl_3$. ^{31}P NMR (number of scans = 64, delay time = 3 s) was measured. A 50 mM solution of **CS** in $CDCl_3$ was added in a stepwise titration (5 µL = 0.2 eq **CS**). The mixture was equilibrated for 15 min before measurement to allow the binding to equilibrate (compare Fig. S60).

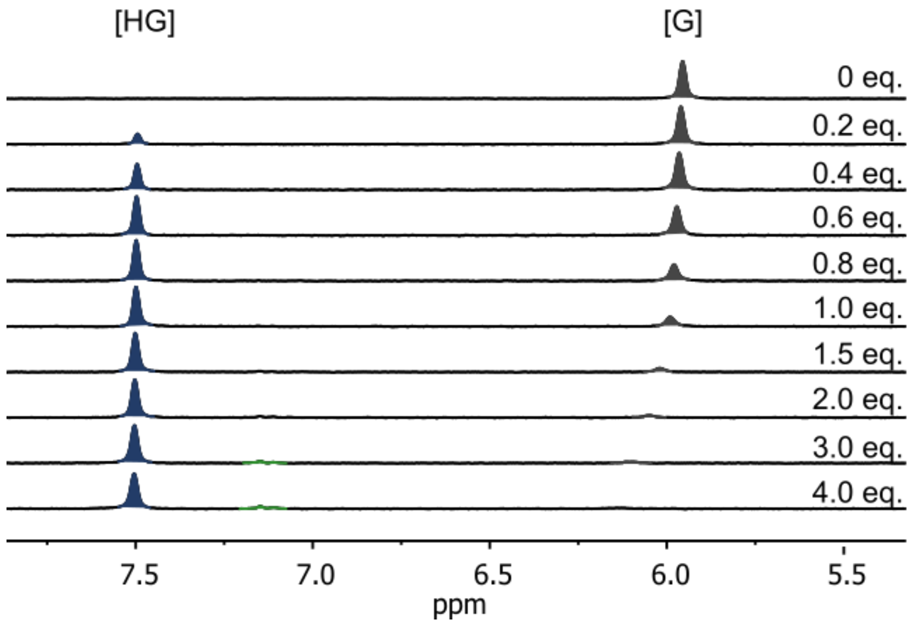

Figure S80: ^{31}P NMR titration of **CS** to F-benzyl-PP **3** (2.5 mM, DCM-d_2).

Table S3: ^{31}P NMR titration in DCM-d_2. Ratios of G and HG were determined by NMR integration, directly giving the amounts of G, HG and H and the corresponding concentrations by considering the total volume. K_A values were estimated according to the mass action law.

**Equiv. CS**	**0.0**	**0.2**	**0.4**	**0.6**	**0.8**	**1.0**	**1.5**	**2.0**	**3.0**	**4.0**
Total volume	500	505	510	515	520	525	537.5	550	575	600
	**free pyrophosphonate (G)**									
Ratio	1.00	0.83	0.65	0.49	0.36	0.26	0.12	0.08	-	-
Moles [µmol]	1.25	1.04	0.81	0.61	0.45	0.15	0.10	0.46	-	-
Conc. [mM]	2.50	2.05	1.59	1.19	0.87	0.62	0.28	0.18	-	-
	**[2]pseudorotaxane (HG)**									
Ratio	0.00	0.17	0.35	0.51	0.64	0.74	0.88	0.92	-	-
Moles [µmol]	0.00	0.21	0.44	0.64	0.80	0.93	1.10	1.15	-	-
Conc. [mM]	0.00	0.82	1.63	2.25	2.69	2.97	3.32	3.41	-	-
	**free CS (H)**									
Moles [µmol]	0.00	0.04	0.06	0.11	0.20	0.33	0.78	1.35	-	-
Conc. [mM]	0.00	0.07	0.12	0.22	0.38	0.62	1.44	2.45	-	-
	**K_A [M^{-1}]**									
$\frac{[HG]}{[G] * [H]}$	-	2760	4390	4760	4620	4600	5090	4690	-	-

The last two data points were left out as the integration accuracy decreased and small amounts of 2:1 binding were observed. K_A was determined as the mean value of the first seven data point with the standard deviation as error to be:

K_A = 4.42 ± 0.7 • 10^3 M^{-1}

DFT calculations

A restricted hybrid HF-DFT SCF calculation was performed using Pulay DIIS + Geometric Direct Minimization with a SPARTAN'20 Quantum Mechanics Driver Software on the RB3LYP function with a 6-31G(D) basis set for the optimized geometries.

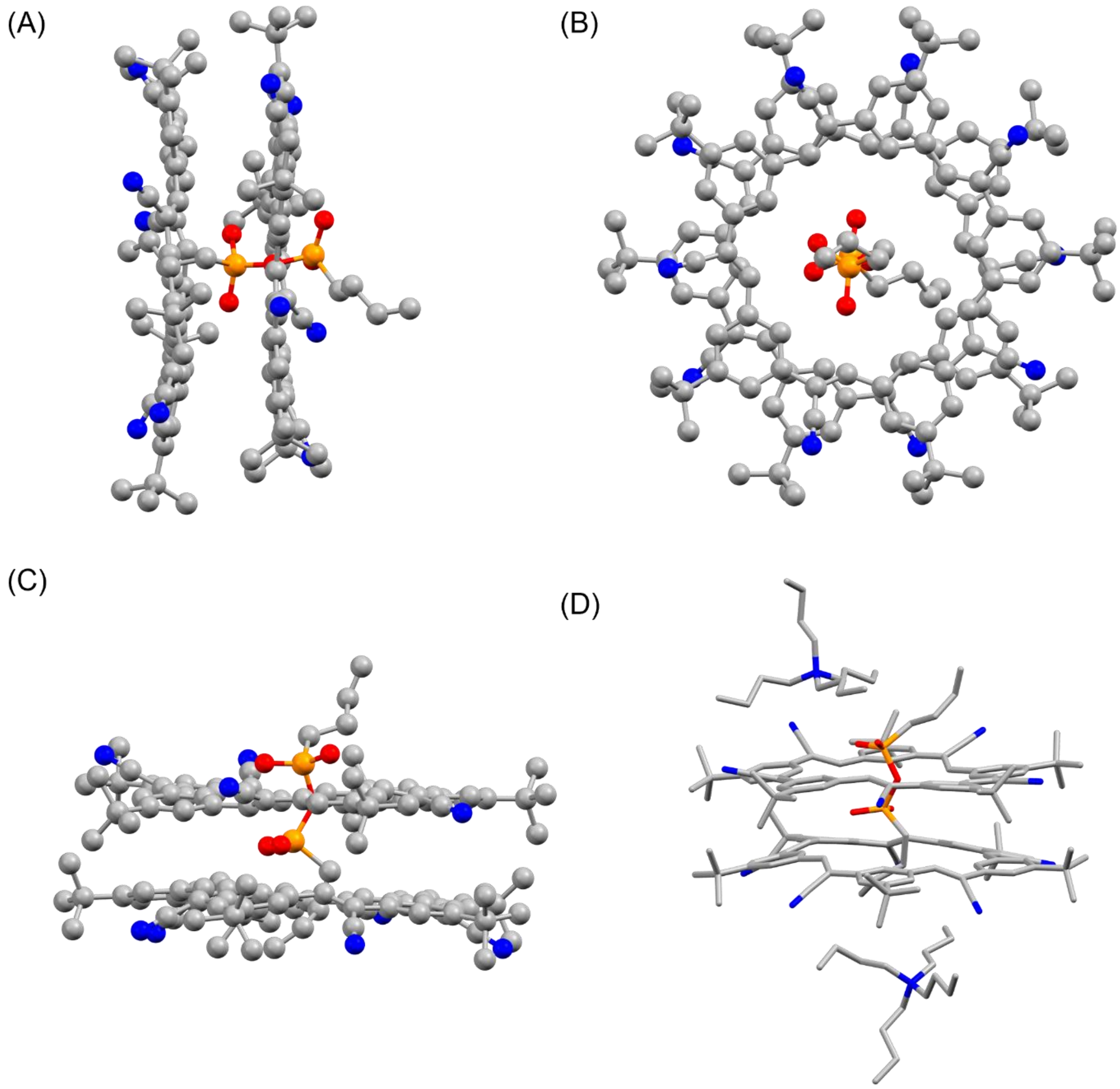

Figure S81: Additional images of [3]pseudorotaxane, assembled of 2x **CS** and homoallyl-PP **1**. Equilibrium geometries were optimized using density functional theory (RB3LYP/6-31G(D), gas phase). Hydrogen atoms and TBA counter ions were omitted for clarity. Color code: C (grey), N (blue), O (red), P (orange).

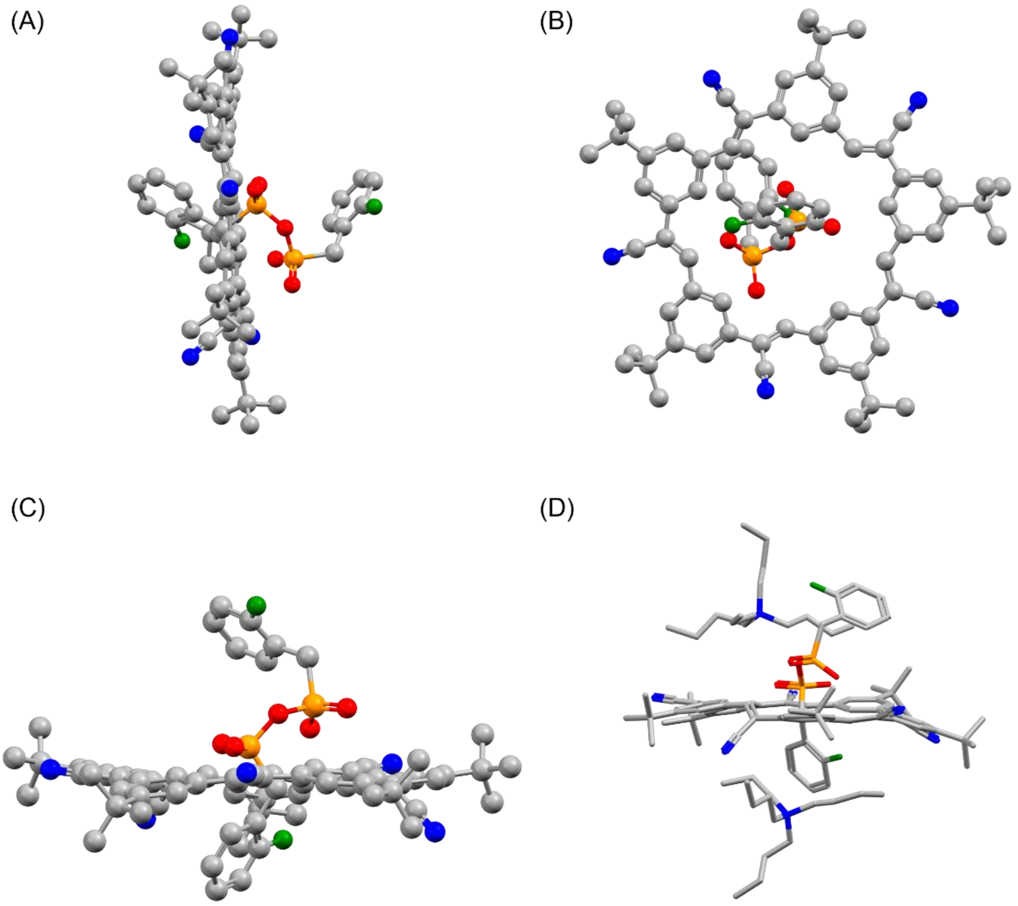

Figure S82: Additional images of [2]pseudorotaxane, assembled of **CS** and F-benzyl-PP **3**. Equilibrium geometries were optimized using density functional theory (RB3LYP/6-31G(D), gas phase). Hydrogen atoms and TBA counter ions were omitted for clarity. Color code: C (grey), N (blue), O (red), F (green), P (orange).

Table S4: Cartesian coordinates of the [3]pseudorotaxane (2x **CS** on homoallyl-PP **1**) in the gas phase, according to density functional theory (RB3LYP/6-31G(D), gas phase).

Atom	Cartesian Coordinates			Atom	Cartesian Coordinates		
H	5.59100	4.15000	-5.50900	H	6.35200	9.88300	3.17300
C	5.38200	3.60800	-4.59000	C	2.47500	6.94800	2.43900
C	4.16100	3.15300	-4.30400	H	1.70300	6.17400	2.43100
C	3.78700	2.39200	-3.06200	H	2.02800	7.85700	2.04200
C	2.66900	3.08800	-2.26500	C	3.01400	7.18200	3.86800
H	1.75000	3.16500	-2.85800	H	2.61500	8.14400	4.21400
H	2.97800	4.12000	-2.05800	H	4.10300	7.30900	3.86100
P	2.25100	2.37400	-0.61400	C	2.63300	6.10500	4.89500
O	1.44200	3.42200	0.13200	H	1.54100	6.00000	4.91200
O	3.49100	1.77800	0.02000	H	3.02100	5.12800	4.58300
O	1.24900	1.08900	-1.05100	C	3.14400	6.43000	6.30000
P	-0.21700	0.67400	-0.37400	H	2.86100	5.63900	7.00000
O	-0.16700	0.75400	1.14000	H	4.23800	6.51700	6.31700
O	-1.32200	1.36400	-1.15500	H	2.73100	7.37900	6.66700
C	-0.24400	-1.10600	-0.85400	N	-3.23500	-7.45600	0.77900
H	0.52100	-1.62400	-0.26400	C	-3.12300	-6.72500	-0.57100
H	0.05900	-1.18300	-1.90500	H	-3.49700	-7.44500	-1.30500
C	-1.62800	-1.76600	-0.67900	H	-2.05400	-6.59400	-0.75100
C	-2.11000	-1.93300	0.73700	C	-3.85800	-5.39700	-0.74700
H	-1.38400	-2.32300	1.44900	H	-3.42300	-4.60900	-0.12400
C	-3.34700	-1.67000	1.16700	H	-4.91300	-5.48700	-0.46500
H	-3.63100	-1.81500	2.20600	C	-3.80100	-4.96500	-2.22700
H	-4.10600	-1.24800	0.51300	H	-4.18000	-5.78200	-2.85700
H	3.34800	3.33400	-5.01100	H	-2.75800	-4.80500	-2.52700
H	6.22400	3.44300	-3.91900	C	-4.62200	-3.69900	-2.49200
H	-1.59000	-2.76800	-1.13400	H	-4.26600	-2.85500	-1.89100
H	-2.37300	-1.19600	-1.24600	H	-4.55600	-3.40600	-3.54400
H	4.66200	2.25600	-2.41800	H	-5.68000	-3.85600	-2.25400
H	3.45600	1.38800	-3.35000	C	-2.58200	-8.83800	0.60800
C	5.69100	-3.58000	-2.41200	H	-2.83000	-9.39300	1.51300
C	4.01000	-1.58400	-1.34600	H	-3.11000	-9.30700	-0.22600
C	5.75700	-3.22200	-1.06100	C	-1.07100	-8.90400	0.38900
C	4.76200	-2.92100	-3.22200	H	-0.75300	-8.35300	-0.50200
C	3.91900	-1.91400	-2.71400	H	-0.53700	-8.47200	1.24300
C	4.92400	-2.23300	-0.50800	C	-0.66400	-10.38500	0.23600
H	3.36100	-0.81000	-0.95000	H	-1.09000	-10.77700	-0.69800
C	4.97700	-1.94700	0.95600	H	-1.11300	-10.97100	1.05100
C	4.40600	-0.84700	1.52700	C	0.85200	-10.59300	0.23700
H	3.99200	-0.10500	0.84400	H	1.28800	-10.29300	1.19700
C	5.62900	-2.95700	1.73800	H	1.09300	-11.65100	0.08200
N	6.19200	-3.79500	2.32100	H	1.33800	-10.01000	-0.55000
C	4.50900	-0.74900	5.34500	C	-2.51100	-6.71000	1.91200
C	3.26500	0.53300	3.16500	H	-1.58800	-6.32300	1.47300
C	3.54900	0.25700	5.54100	H	-2.22900	-7.47900	2.63100

C	4.81800	-1.11700	4.03500	C	-3.26400	-5.59300	2.63700
C	4.19500	-0.49600	2.93200	H	-4.23400	-5.94700	3.00200
C	2.91300	0.90300	4.47200	H	-3.44800	-4.74400	1.97300
H	2.84200	1.04200	2.30500	C	-2.44000	-5.11000	3.85200
C	1.90900	1.97600	4.72800	H	-2.87800	-4.16700	4.19700
C	0.99100	2.37800	3.80500	H	-1.42100	-4.86600	3.52900
H	0.99100	1.85500	2.85000	C	-2.39200	-6.09500	5.02700
C	1.98500	2.58900	6.02300	H	-1.94800	-7.06000	4.75300
N	2.08200	3.05600	7.08700	H	-1.78100	-5.68700	5.83700
C	-1.41000	5.09500	5.01300	H	-3.39800	-6.28900	5.42000
C	-0.39100	3.98700	2.62400	C	-4.71400	-7.68300	1.09100
C	-1.75300	5.62700	3.75700	H	-5.11600	-8.18300	0.20500
C	-0.53900	4.00200	5.04700	H	-5.16500	-6.69600	1.14500
C	0.00600	3.46300	3.86300	C	-5.03700	-8.49300	2.36500
C	-1.28200	5.06900	2.56000	H	-5.77500	-7.92400	2.94200
H	0.04600	3.57000	1.72000	H	-4.16200	-8.56900	3.02000
C	-1.72500	5.60700	1.24100	C	-5.61000	-9.89300	2.09000
C	-1.73100	4.84400	0.11400	H	-6.50000	-9.79100	1.45500
H	-1.36500	3.82800	0.22500	H	-4.89100	-10.48900	1.51200
C	-2.11100	6.98800	1.22300	C	-5.98000	-10.63900	3.37400
N	-2.38600	8.12100	1.24900	H	-6.42000	-11.61500	3.14600
C	-3.35100	6.42200	-2.95800	H	-5.10200	-10.81000	4.00700
C	-1.57900	4.34800	-2.25600	H	-6.71100	-10.07400	3.96400
C	-2.75700	5.60700	-3.93600	C	-5.91200	3.98200	1.47000
C	-3.02200	6.19100	-1.62100	C	-4.09500	2.08000	0.45800
C	-2.12800	5.16400	-1.25700	C	-5.96400	3.57000	0.13300
C	-1.87700	4.56700	-3.60700	C	-4.95500	3.39000	2.30300
H	-0.92400	3.54200	-1.94900	C	-4.05600	2.42500	1.82100
C	-1.26900	3.71800	-4.67100	C	-5.06200	2.62700	-0.39200
C	-0.71400	2.49900	-4.41800	H	-3.36900	1.37100	0.08000
H	-0.77000	2.15700	-3.38600	C	-5.17800	2.18900	-1.81500
C	-1.26600	4.29100	-5.98700	C	-4.16900	1.57700	-2.49700
N	-1.29100	4.79500	-7.03700	H	-3.22300	1.47200	-1.96600
C	0.63000	0.68900	-7.49800	C	-6.47100	2.39500	-2.40600
C	0.87600	0.66600	-4.69100	N	-7.53400	2.58300	-2.84600
C	1.51700	-0.18100	-6.85400	C	-5.06300	0.63300	-6.09200
C	-0.12300	1.56200	-6.70600	C	-3.20400	-0.02200	-4.08300
C	0.00000	1.57800	-5.30700	C	-4.12800	-0.39000	-6.28000
C	1.66000	-0.21000	-5.45400	C	-5.07700	1.29400	-4.85900
H	0.94900	0.68900	-3.60900	C	-4.17300	0.96800	-3.83300
C	2.65600	-1.13100	-4.83100	C	-3.19000	-0.73700	-5.28900
C	2.93500	-1.16400	-3.49500	H	-2.46100	-0.20100	-3.31400
C	3.35800	-1.97700	-5.75400	C	-2.23800	-1.86000	-5.53900
N	3.89400	-2.65300	-6.53600	C	-1.37800	-2.35500	-4.60400
C	-1.98400	5.73500	6.29100	H	-1.42700	-1.87100	-3.63500
C	5.17800	-1.40400	6.56800	C	-2.31100	-2.46000	-6.84200
C	-4.34000	7.52000	-3.38900	N	-2.40100	-2.93700	-7.90100

C	0.47800	0.73600	-9.03100	C	0.87100	-5.26000	-5.68600
C	6.58900	-4.67400	-3.02100	C	0.04900	-3.89700	-3.37600
C	-0.99400	0.46200	-9.42000	C	1.29800	-5.67000	-4.41100
H	-1.30900	-0.54100	-9.11000	C	0.01000	-4.16200	-5.77500
H	-1.67800	1.18800	-8.96800	C	-0.43100	-3.47400	-4.62600
H	-1.11100	0.53000	-10.50900	C	0.91400	-4.98900	-3.24800
C	1.36300	-0.30400	-9.74300	H	-0.28000	-3.36600	-2.49200
H	1.11500	-1.32800	-9.44100	C	1.39700	-5.42800	-1.90400
H	1.21000	-0.23400	-10.82600	C	1.47100	-4.59800	-0.82700
H	2.42900	-0.13800	-9.55000	H	1.17900	-3.57100	-1.02000
C	0.88400	2.14500	-9.52700	C	1.73400	-6.81700	-1.80400
H	0.26600	2.93100	-9.08100	N	1.97300	-7.95700	-1.77300
H	1.93000	2.36000	-9.27900	C	2.96200	-6.09800	2.35000
H	0.77400	2.20900	-10.61700	C	1.32000	-3.96400	1.53200
H	-0.82400	2.23600	-7.18200	C	2.38600	-5.21900	3.28100
H	2.12000	-0.85700	-7.44800	C	2.67100	-5.90300	0.99700
C	-5.52400	6.87400	-4.14800	C	1.83900	-4.84800	0.57000
H	-6.24900	7.64000	-4.44700	C	1.57400	-4.14000	2.89900
H	-5.19300	6.35800	-5.05600	H	0.68700	-3.14800	1.20300
H	-6.04400	6.14200	-3.51800	C	0.94800	-3.26500	3.93800
C	-3.61900	8.52100	-4.32300	C	0.46200	-2.01100	3.70700
H	-2.79400	9.02000	-3.80000	H	0.61200	-1.60500	2.70900
H	-3.20500	8.03000	-5.20900	C	0.79100	-3.89700	5.21700
H	-4.31800	9.29300	-4.66600	N	0.67200	-4.47300	6.22300
C	-4.90600	8.30200	-2.18900	C	-1.19600	-0.39400	6.74100
H	-5.59000	9.08000	-2.54600	C	-1.20600	-0.24900	3.92300
H	-4.11900	8.79300	-1.60600	C	-2.09400	0.43600	6.05700
H	-5.47300	7.65200	-1.51200	C	-0.33000	-1.18900	5.98200
C	-1.46800	7.19100	6.39800	C	-0.33600	-1.14000	4.57700
H	-1.74300	7.78700	5.52100	C	-2.11700	0.52400	4.65300
H	-1.89300	7.68100	7.28300	H	-1.15700	-0.17500	2.84300
H	-0.37600	7.21200	6.49400	C	-3.12500	1.38700	3.96900
C	-1.56100	4.98000	7.56500	C	-3.07700	1.70400	2.64400
H	-0.47300	4.95800	7.69500	C	-4.21800	1.81500	4.79500
H	-1.99200	5.47500	8.44300	N	-5.08000	2.13600	5.51200
H	-1.92400	3.94500	7.55900	C	1.33600	-6.03800	-6.93100
C	-3.53000	5.74700	6.23200	C	-6.07200	1.04500	-7.18000
H	-3.93800	6.19800	7.14500	C	3.84800	-7.25600	2.84700
H	-3.90100	6.33200	5.38400	C	-1.15300	-0.47900	8.27900
H	-3.93200	4.73200	6.14800	C	-6.87300	5.03800	2.04900
C	5.89100	-0.31900	7.40900	C	-1.49800	-1.92300	8.71600
H	6.36500	-0.77100	8.28900	H	-2.51100	-2.19700	8.39800
H	6.67200	0.18200	6.82500	H	-0.80400	-2.65600	8.29200
H	5.19600	0.44900	7.76600	H	-1.45200	-2.00800	9.80800
C	4.09800	-2.09100	7.43700	C	-2.15200	0.48100	8.95000
H	3.34700	-1.37800	7.79600	H	-3.18800	0.26100	8.66900
H	4.55700	-2.55700	8.31800	H	-2.07800	0.38400	10.03900

H	3.57300	-2.87500	6.87800
C	6.22000	-2.46400	6.16800
H	7.03700	-2.03400	5.57800
H	5.78000	-3.27900	5.58400
H	6.66100	-2.90400	7.07000
C	5.70100	-5.83400	-3.53200
H	5.00000	-5.50200	-4.30400
H	5.11900	-6.27300	-2.71300
H	6.32500	-6.62500	-3.96400
C	7.58900	-5.24900	-2.00100
H	7.08500	-5.71600	-1.14700
H	8.27000	-4.48100	-1.61600
H	8.19900	-6.02000	-2.48400
C	7.39100	-4.08400	-4.20500
H	8.02000	-3.24900	-3.87600
H	8.04500	-4.85100	-4.63800
H	6.73800	-3.71500	-5.00300
H	4.69100	-3.19900	-4.26300
H	6.46400	-3.71900	-0.40800
H	5.56100	-1.88200	3.86100
H	3.29100	0.55000	6.55300
H	-0.28300	3.55200	5.99400
H	-2.42300	6.48000	3.70500
H	-3.47000	6.79600	-0.84500
H	-2.99200	5.77700	-4.98200
H	2.34900	-0.48600	-2.88000
N	3.50500	6.50600	1.40300
C	2.80200	6.26300	0.05400
H	2.17200	5.37600	0.20000
H	3.60800	5.97400	-0.62500
C	1.99700	7.40700	-0.55900
H	2.62700	8.27900	-0.77100
H	1.19400	7.73600	0.11000
C	1.36000	6.93300	-1.87900
H	0.75100	6.04400	-1.68300
H	2.15200	6.62100	-2.57400
C	0.49900	8.01800	-2.53000
H	1.09100	8.91000	-2.77400
H	0.05000	7.64900	-3.45700
H	-0.31700	8.32400	-1.86600
C	4.07500	5.14800	1.85500
H	4.46600	5.32100	2.86000
H	3.21700	4.47400	1.91200
C	5.15900	4.52000	0.97800
H	4.72500	4.13800	0.05100
H	5.94500	5.24100	0.72000
C	5.79300	3.33300	1.72600

H	-1.94400	1.52700	8.69800
C	0.26700	-0.12000	8.77800
H	1.02200	-0.80500	8.37700
H	0.54900	0.89900	8.48700
H	0.30900	-0.18500	9.87200
H	0.37100	-1.84100	6.48700
H	-2.79700	1.03800	6.61900
C	2.94000	-8.30500	3.53300
H	3.54300	-9.14000	3.91000
H	2.39600	-7.87200	4.38000
H	2.20200	-8.71100	2.82900
C	4.89300	-6.74400	3.86500
H	5.50800	-5.95100	3.43200
H	4.42800	-6.35700	4.77800
H	5.54800	-7.57100	4.16400
C	4.60900	-7.93800	1.69200
H	5.24100	-8.73800	2.09400
H	5.26200	-7.22800	1.17400
H	3.93700	-8.39300	0.95600
C	2.87700	-6.17700	-6.93000
H	3.24100	-6.73600	-6.06200
H	3.19900	-6.72200	-7.82600
H	3.36100	-5.19500	-6.93500
C	0.92500	-5.33900	-8.24100
H	1.35800	-4.33600	-8.31200
H	1.29400	-5.91900	-9.09400
H	-0.16200	-5.25300	-8.35000
C	0.69300	-7.44500	-6.89900
H	1.01400	-8.03000	-7.76900
H	0.98000	-8.00100	-5.99900
H	-0.40100	-7.37700	-6.92000
C	-5.92600	0.20600	-8.46400
H	-6.66600	0.53700	-9.20200
H	-4.93500	0.31700	-8.91800
H	-6.09900	-0.86000	-8.28000
C	-7.51000	0.85600	-6.63900
H	-7.70000	-0.19600	-6.39600
H	-8.24100	1.16500	-7.39700
H	-7.69500	1.44600	-5.73600
C	-5.85100	2.53200	-7.54500
H	-4.83400	2.70100	-7.92000
H	-6.00300	3.19200	-6.68300
H	-6.55700	2.83800	-8.32700
C	-7.70400	4.39800	3.18700
H	-7.07200	4.02500	4.00000
H	-8.29400	3.55300	2.81200
H	-8.39600	5.13500	3.61000

H	4.99600	2.65000	2.04000	C	-7.84700	5.59100	0.99100
H	6.29400	3.70000	2.63400	H	-8.49200	4.80900	0.57600
C	6.78300	2.56300	0.85000	H	-7.31800	6.07400	0.16100
H	7.58300	3.21500	0.47400	H	-8.49500	6.34400	1.45200
H	7.25200	1.74800	1.41200	C	-6.05800	6.22500	2.61500
H	6.25800	2.12400	-0.00300	H	-5.45500	6.70400	1.83600
C	4.65400	7.50700	1.27300	H	-6.73500	6.98100	3.03200
H	5.10700	7.31200	0.29800	H	-5.38100	5.91200	3.41700
H	5.38700	7.22600	2.03100	H	-4.89600	3.69500	3.34000
C	4.32600	8.99600	1.41000	H	-6.71100	3.99200	-0.53000
H	3.95800	9.21400	2.41900	H	-5.79400	2.08700	-4.69600
H	3.53700	9.29000	0.71400	H	-4.11800	-0.93200	-7.21700
C	5.57100	9.86500	1.13400	H	-0.32400	-3.82900	-6.74500
H	5.24500	10.91200	1.12800	H	1.95800	-6.52700	-4.32100
H	5.94300	9.65800	0.12100	H	3.11200	-6.55400	0.25500
C	6.70800	9.70500	2.15100	H	2.58200	-5.36700	4.33700
H	7.16000	8.70600	2.12400	H	-2.21900	1.33400	2.08500
H	7.50900	10.42300	1.94900				

Table S5: Cartesian coordinates of the [2]pseudorotaxane (1 x **CS** on F-benzyl-PP **3**) in the gas phase, according to density functional theory (RB3LYP/6-31G(D), gas phase).

Atom	Cartesian Coordinates			Atom	Cartesian Coordinates		
H	3.78700	6.03800	-3.26400	H	1.85000	-2.82500	7.89800
C	3.44900	5.06100	-3.59400	C	2.49000	-4.85600	6.14600
C	2.65100	2.50200	-4.37200	H	1.40700	-4.77000	6.29800
C	2.65400	4.89800	-4.72900	H	2.88700	-5.53700	6.91000
C	3.81800	3.93100	-2.87600	H	2.65400	-5.31500	5.16600
C	3.46200	2.63000	-3.23200	C	4.69500	-3.63200	6.06600
C	2.24800	3.61600	-5.10800	H	5.20700	-2.66500	6.12600
H	2.32500	1.50600	-4.65300	H	4.93900	-4.08000	5.09700
H	2.35200	5.76700	-5.30700	H	5.10800	-4.28400	6.84500
H	1.61600	3.48100	-5.98000	C	3.18500	-7.35400	-3.95600
F	4.57500	4.12600	-1.74500	H	2.57100	-6.89900	-4.74000
C	3.96800	1.43400	-2.47000	H	2.51300	-7.84000	-3.23700
H	4.63800	1.74100	-1.66500	H	3.80200	-8.13300	-4.42200
H	4.56300	0.80100	-3.14100	C	4.97500	-7.03400	-2.23300
P	2.72900	0.24600	-1.77000	H	4.38500	-7.55900	-1.47300
O	3.50500	-0.75800	-0.94800	H	5.65300	-6.34100	-1.72300
O	1.75300	-0.18700	-2.83700	H	5.58900	-7.77900	-2.75100
O	1.90800	1.20900	-0.65900	C	5.00000	-5.63700	-4.30500
P	0.29300	1.41800	-0.39700	H	5.65300	-4.89500	-3.83200
O	0.24200	1.94300	1.04000	H	5.63400	-6.39300	-4.78700
O	-0.37300	2.20200	-1.49600	H	4.42900	-5.13100	-5.09000
C	-0.38800	-0.30700	-0.40200	H	2.34500	-4.62600	-4.47300
H	0.13700	-0.87500	0.37600	H	3.84300	-5.57100	-0.56100
H	-0.09600	-0.72000	-1.36500	H	3.32300	-3.78400	3.56300
C	-1.88000	-0.41200	-0.19600	H	1.74100	-1.09300	6.49000
C	-4.69100	-0.64900	0.18100	H	-1.30200	2.45900	6.36300
C	-2.52900	0.15500	0.91500	H	-3.68600	5.41500	4.36900
C	-2.70700	-1.10400	-1.08500	H	-4.20600	6.41600	-0.37300
C	-4.08200	-1.23300	-0.92900	H	-4.45300	5.06700	-4.43100
C	-3.90700	0.04300	1.10700	H	-0.01800	-1.99000	-2.98000
H	-5.76600	-0.72800	0.31400	N	2.72000	4.81200	1.69200
H	-1.92700	0.70300	1.62900	C	1.90800	4.61000	0.39600
H	-4.65100	-1.77200	-1.68200	H	1.10500	3.92300	0.67100
H	-4.36700	0.50700	1.97500	H	2.57500	4.07100	-0.27400
F	-2.14900	-1.72700	-2.16700	C	1.36800	5.85700	-0.30500
C	3.19000	-5.23500	-2.59300	H	2.18800	6.44200	-0.74000
C	1.55300	-3.25300	-1.47500	H	0.83100	6.51600	0.38900
C	3.19400	-4.99700	-1.21200	C	0.39700	5.44700	-1.42700
C	2.35000	-4.45500	-3.40400	H	-0.41900	4.85500	-1.00500
C	1.52600	-3.45300	-2.86900	H	0.90200	4.77700	-2.13000
C	2.39500	-3.98900	-0.64400	C	-0.16800	6.65700	-2.17400
H	0.92700	-2.48600	-1.04200	H	0.62400	7.23400	-2.67000
C	2.40300	-3.71400	0.83100	H	-0.87700	6.33300	-2.94300
C	2.41000	-2.45600	1.36100	H	-0.70300	7.33400	-1.49600
H	2.52300	-1.64600	0.63200	C	3.17500	3.42000	2.16600
C	2.28700	-4.89200	1.64000	H	3.57600	3.57900	3.16900
N	2.14300	-5.89300	2.22400	H	2.26000	2.82500	2.22800
C	2.60700	-2.54500	5.16500	C	4.20600	2.68400	1.31100
C	1.63300	-0.86800	3.10900	H	3.72900	2.29800	0.40900

C	1.90700	-1.37000	5.45700
C	2.77700	-2.88400	3.81800
C	2.28400	-2.07600	2.78000
C	1.40100	-0.52300	4.45200
H	1.30900	-0.20900	2.30800
C	0.60400	0.67900	4.86100
C	-0.19800	1.38000	4.00700
H	-0.13300	1.09000	2.96300
C	0.71600	1.06300	6.23900
N	0.84800	1.37700	7.35400
C	-2.53500	4.00600	5.52500
C	-1.58700	3.12000	3.03700
C	-2.97300	4.59800	4.32600
C	-1.62700	2.94600	5.45500
C	-1.13200	2.49500	4.21200
C	-2.52700	4.15800	3.07200
H	-1.14900	2.79600	2.10000
C	-3.04700	4.75700	1.80200
C	-2.93300	4.12500	0.59500
H	-2.43000	3.16500	0.62900
C	-3.69500	6.02700	1.94500
N	-4.20100	7.06200	2.12200
C	-4.40200	5.90300	-2.44700
C	-3.10300	3.50200	-1.75000
C	-4.14100	4.90700	-3.40300
C	-4.00900	5.67000	-1.12900
C	-3.36200	4.47000	-0.76100
C	-3.48600	3.71000	-3.08100
H	-2.57300	2.59900	-1.47700
C	-3.24800	2.67300	-4.13800
C	-2.18100	1.82000	-4.14200
H	-1.47000	1.94900	-3.32500
C	-4.27100	2.63700	-5.14300
N	-5.13100	2.65500	-5.93100
C	-2.14200	-0.58200	-7.12700
C	-0.84300	-0.18100	-4.65100
C	-1.17200	-1.47900	-6.66800
C	-2.47400	0.50200	-6.30300
C	-1.85000	0.71800	-5.06100
C	-0.53000	-1.30700	-5.42900
H	-0.27400	0.02600	-3.74900
C	0.44400	-2.33500	-4.94900
C	0.62400	-2.57400	-3.62100
C	1.16100	-3.06400	-5.95200
N	1.71000	-3.67300	-6.78000
C	-3.06600	4.54700	6.86700
C	3.16900	-3.47100	6.25900
C	-5.10100	7.20500	-2.88400
C	-2.86300	-0.74800	-8.47800
C	4.08100	-6.30600	-3.25400
C	-4.38300	-0.90200	-8.23000
H	-4.59000	-1.79000	-7.62000

H	5.03300	3.33700	1.00900
C	4.77900	1.50300	2.11900
H	3.95400	0.87400	2.47600
H	5.28400	1.89400	3.01600
C	5.74900	0.64500	1.30100
H	6.56900	1.25300	0.89700
H	6.19400	-0.13500	1.92900
H	5.22900	0.15400	0.47000
C	3.94300	5.69400	1.45000
H	4.29700	5.43500	0.45000
H	4.70500	5.36900	2.16100
C	3.75000	7.21000	1.56900
H	3.51700	7.48300	2.60400
H	2.91200	7.54300	0.95300
C	5.01700	7.96800	1.12500
H	4.78100	9.03900	1.13300
H	5.24200	7.71500	0.08000
C	6.25700	7.72200	1.99300
H	6.60300	6.68300	1.94200
H	7.08800	8.35400	1.66500
H	6.05700	7.95800	3.04600
C	1.79400	5.37700	2.76600
H	0.95400	4.68000	2.81500
C	1.41200	6.31500	2.36700
C	2.41300	5.59400	4.16400
H	2.13000	6.60100	4.49700
H	3.50900	5.60800	4.11300
C	1.96000	4.58800	5.23400
H	0.86600	4.61200	5.30500
H	2.21700	3.56700	4.92600
C	2.56900	4.86900	6.61000
H	2.21500	4.13500	7.34200
H	3.66400	4.81400	6.58400
H	2.29500	5.86800	6.97200
N	-1.73500	-6.69300	-0.17000
C	-1.49100	-6.38200	-1.65800
H	-1.67500	-7.33000	-2.17400
H	-0.42800	-6.15300	-1.74500
C	-2.32400	-5.27900	-2.31100
H	-2.07500	-4.29300	-1.90800
H	-3.39400	-5.44400	-2.14500
C	-2.07800	-5.26100	-3.83200
H	-2.22900	-6.27000	-4.24200
H	-1.03500	-4.99100	-4.02900
C	-3.01100	-4.27100	-4.53700
H	-2.93000	-3.27400	-4.09500
H	-2.76200	-4.18600	-5.59900
H	-4.05700	-4.59400	-4.46200
C	-0.99400	-7.99500	0.15400
H	-1.23600	-8.21900	1.19400
H	-1.46400	-8.76000	-0.47200
C	0.52200	-8.01100	-0.02200

H	-4.80200	-0.03400	-7.71200
H	-4.91500	-1.01400	-9.18300
C	-2.37100	-1.98400	-9.25600
H	-2.56400	-2.91500	-8.70900
H	-2.89700	-2.05100	-10.21500
H	-1.29700	-1.93200	-9.47100
C	-2.61600	0.50400	-9.35300
H	-2.98000	1.41500	-8.86800
H	-1.54700	0.63500	-9.55800
H	-3.13900	0.40500	-10.31200
H	-3.22000	1.20400	-6.64700
H	-0.89500	-2.33600	-7.27200
C	-6.48600	6.87000	-3.48400
H	-6.99100	7.78900	-3.80700
H	-6.41000	6.21000	-4.35400
H	-7.12400	6.37500	-2.74300
C	-4.23500	7.91200	-3.95300
H	-3.24300	8.15400	-3.55200
H	-4.09400	7.29100	-4.84300
H	-4.71100	8.84800	-4.27100
C	-5.30800	8.18200	-1.71200
H	-5.81000	9.08700	-2.07400
H	-4.35900	8.49100	-1.25900
H	-5.93300	7.75100	-0.92100
C	-2.62400	6.02100	7.02400
H	-3.00000	6.64900	6.20900
H	-3.00000	6.43400	7.96700
H	-1.53100	6.10000	7.03200
C	-2.53300	3.75100	8.07300
H	-1.44000	3.78100	8.14100
H	-2.93400	4.18000	8.99900
H	-2.84200	2.70000	8.03600
C	-4.61200	4.46900	6.88700
H	-4.99800	4.83400	7.84600
H	-5.06600	5.07500	6.09600
H	-4.95200	3.43500	6.75700
C	2.91900	-2.92300	7.67800
H	3.34500	-3.61200	8.41600
H	3.38800	-1.94500	7.82800

H	0.80500	-7.89300	-1.07400
H	0.99100	-7.19900	0.54200
C	1.07600	-9.34800	0.50900
H	0.62000	-10.18500	-0.04000
H	0.77800	-9.46300	1.56000
C	2.60100	-9.42900	0.40700
H	3.07000	-8.61800	0.97500
H	2.96900	-10.38000	0.80700
H	2.93500	-9.35400	-0.63500
C	-1.19700	-5.57000	0.74400
H	-0.30200	-5.18800	0.24900
H	-0.87300	-6.06200	1.66100
C	-2.15100	-4.42300	1.08000
H	-3.06300	-4.80400	1.55400
H	-2.45400	-3.88400	0.17800
C	-1.48900	-3.42400	2.05300
H	-2.15800	-2.56000	2.12400
H	-0.55700	-3.04800	1.61600
C	-1.21700	-3.97700	3.45800
H	-0.41400	-4.72300	3.47100
H	-0.90400	-3.16900	4.12600
H	-2.12000	-4.43000	3.88800
C	-3.22500	-6.94300	0.04700
H	-3.50800	-7.68100	-0.71000
H	-3.72200	-6.01000	-0.20400
C	-3.64600	-7.41700	1.45300
H	-4.53300	-6.83700	1.73500
H	-2.88700	-7.15300	2.19900
C	-3.99600	-8.91100	1.56200
H	-4.76900	-9.14800	0.81800
H	-3.12700	-9.53100	1.30700
C	-4.49800	-9.28700	2.95900
H	-4.76000	-10.34900	3.01000
H	-3.73500	-9.09500	3.72300
H	-5.39000	-8.70900	3.22800
H	0.98779	6.14463	1.39957
H	2.21238	7.02054	2.28628
H	0.66035	6.70183	3.02295

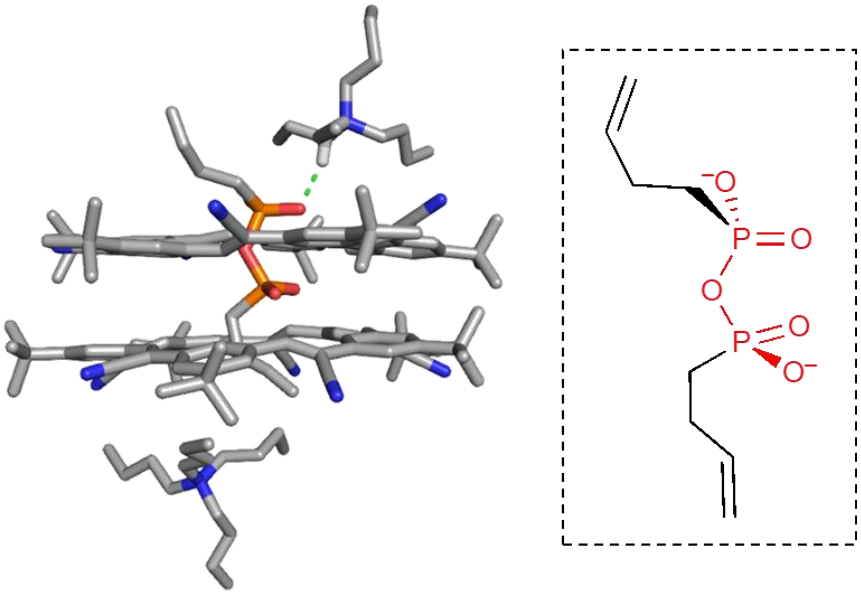

Figure S83: Geometry optimization showing the hydrogen bonding between the α-proton of TBA^+ and the oxygen of the pyrophosphonates moiety in the [3]pseudorotaxane of homoallyl-PP **1** and cyanostar.

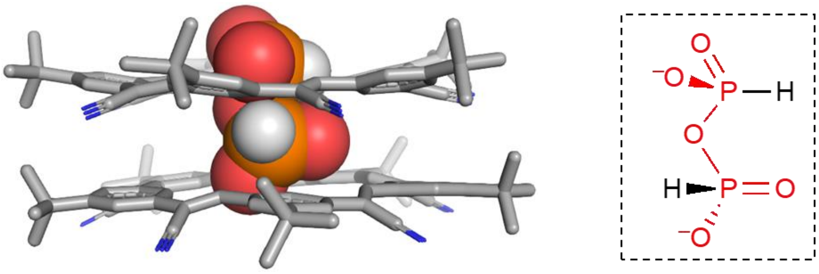

Figure S84: Geometry optimization of a 2:1 complex between cyanostar and $H_2P_2O_5^{2-}$ (RB3LYP/6-31G(D), gas phase).

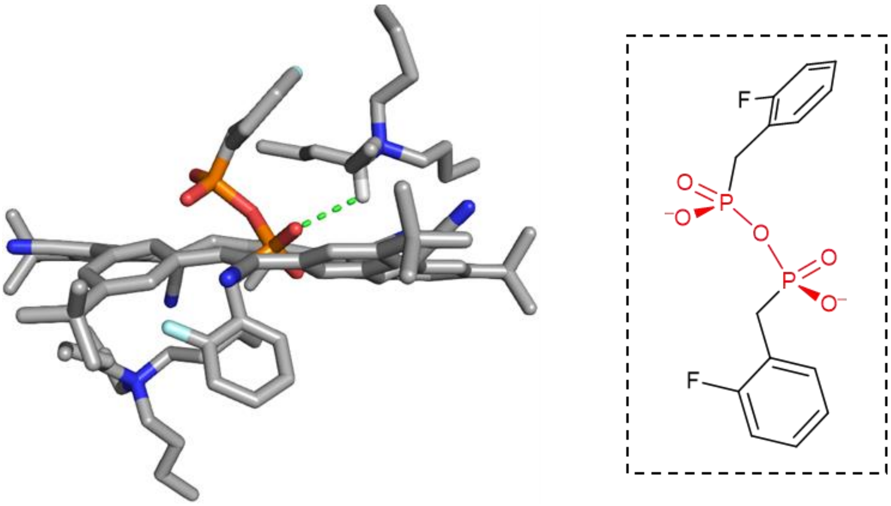

Figure S85: Geometry optimization showing the hydrogen bonding between the α-proton of TBA^+ and the oxygen of the pyrophosphonates moiety in the [2]pseudorotaxane of F-benzyl-PP **3** and cyanostar.

References

[1] L. Du, L. Ma, F. Qi, X. Zheng, C. Jiang, A. Li, X. Wan, S.-J. Liu, S. Li, *J. Bio. Chem.* **2016**, 291, 6583-6594

[2] W. Zhoa, B. Qiao, J. Tropp, M. Pink, J. D. Azoulay, A. H. Flood, *J. Am. Chem. Soc.* **2019**, 141, 4980-4989

[3] S. Lee, C.-H. Chen, A. H. Flood, *Nat. Chem.* **2013**, 5, 704-710

[4] W. Zhao, B. Qiao, C.-H. Chen, A. H. Flood, *Angew. Chem. Int. Ed*. **2017**, 56, 13083-13087

[5] Y. Marcus, *J. Chem. Soc. Faraday Trans.* **1991**, 87, 2995-2999

[6] http://www.mmass.org/, Jan.2023
